# An Isogenic Human Myoblast Cell Model for Cystinosis Myopathy Reveals Alteration of Key Myogenic Regulatory Proteins

**DOI:** 10.1002/jcsm.70116

**Published:** 2025-11-10

**Authors:** Louise Medaer, Roger Mora, Zhuoheng Zhou, Nefele Giarratana, Laura Yedigaryan, Rita La Rovere, Elena Levtchenko, Vincent Mouly, Els Verhoeyen, Sebastiaan Eeltink, Achim Treumann, Tim Vervliet, Maurilio Sampaolesi, Rik Gijsbers

**Affiliations:** ^1^ Advanced Disease Modelling, Targeted Drug Discovery and Gene Therapy (ADVANTAGE), Department of Pharmacological and Pharmaceutical Sciences, Faculty of Medicine KU Leuven Leuven Belgium; ^2^ Department of Chemical Engineering Vrije Universiteit Brussel (VUB) Brussels Belgium; ^3^ Stem Cell and Developmental Biology Unit, Department of Development and Regeneration, Faculty of Medicine KU Leuven Leuven Belgium; ^4^ Laboratory for Molecular and Cellular Signaling, Department of Cellular and Molecular Medicine, Faculty of Medicine KU Leuven Leuven Belgium; ^5^ Emma Children Hospital Amsterdam UMC Amsterdam AZ the Netherlands; ^6^ Myoline Platform, Sorbonne Université, Inserm Institut de Myologie, Centre de Recherche en Myologie Paris France; ^7^ Université Côte D'azur, Institute for health and medical research (INSERM), Mediterranean Centre for Molecular Medicine (C3M) Nice France; ^8^ International Center for Infectiology Research (CIRI) Université de Lyon Lyon France; ^9^ INSERM U1111; Ecole normale supérieure (ENS) de Lyon Université de Lyon Lyon France; ^10^ Centre national de la recherche scientifique (CNRS), UMR5308 Lyon France; ^11^ Cystinosis Ireland Dublin 2 Ireland; ^12^ Histology and Medical Embryology Unit, Department of Anatomy, Histology, Forensic Medicine and Orthopedics Sapienza University of Rome Rome Italy; ^13^ Leuven Viral Vector Core, Group Biomedical Sciences, Faculty of Medicine KU Leuven Leuven Belgium

**Keywords:** CTNS, cystinosis, gene therapy, multiomics, myopathy, viral vectors

## Abstract

**Background:**

Cystinosis is a rare multisystem, autosomal recessive disease caused by dysfunction or loss of cystinosin (CTNS), which results in lysosomal cystine accumulation, primarily affecting the kidneys. Advances in renal transplantation, cysteamine treatment and improved medical care have increased life expectancy, revealing additional systemic phenotypes like myopathy later in life. Muscle weakness is a major concern leading to life‐threatening events in patients, and yet the aetiology of cystinosis myopathy remains to be elucidated.

**Methods:**

We generated human muscle cell‐based models using CRISPR technology to explore the pathophysiology of cystinosis myopathy with the potential to develop new therapies. We used a 4‐day differentiation protocol of myoblasts into myotubes to study the effect of CTNS loss in key regulators of myogenic differentiation using western blot analysis. Afterwards, we used lentiviral (LV)‐mediated *CTNS* ^*WT*^ cDNA addition in *CTNS* ^*−/−*^ cells to corroborate the CTNS‐specific effect. As a next step, we performed multiomic analysis (proteomics, transcriptomics and metabolomics) to gain in‐depth knowledge of affected mechanisms.

**Results:**

The polyclonal, isogenic human *CTNS* knock‐out (KO; *CTNS* ^*−/−*^) myoblasts exhibited unaltered growth characteristics and accumulated cystine. Early‐stage differentiation of myoblasts into myotubes showed a mild reduction in the fusion index of *CTNS* ^*−/−*^ myotubes. Upon examination of several key regulators of myogenic differentiation, we observed significantly decreased myosin heavy chain (MyHC) and ryanodine receptor (RyR) protein levels in *CTNS* ^*−/−*^ myotubes compared to WT cells. Complementation with *CTNS* ^*WT*^ cDNA addition in *CTNS* ^*−/−*^ cells rescued the fusion index, cystine and altered protein levels to WT. In addition, proteomic analysis showed no differences at myoblast level upon the loss of CTNS, but following myotube differentiation, *CTNS* deletion led to an increase of five protein groups mainly involved in oxidative stress pathways, and a decrease of 18 protein groups biologically connected in myofibril assembly and muscle cell differentiation processes. Importantly, LV‐mediated *CTNS* addback reverted protein levels to WT levels. Moreover, metabolomics revealed a distinct clustering resulting from CTNS loss.

**Conclusions:**

Muscle‐specific complications are often overlooked in systemic cystinosis treatment. We show that defective CTNS function impairs effective cystine mobilization from lysosomes, thereby affecting the protein levels of myogenic regulators. A deeper understanding of the molecular mechanisms underlying cystinosis myopathy holds promise for the development of targeted, personalized therapies to improve the quality of life for patients living with cystinosis.

## 1 Introduction

Cystinosis is an autosomal recessive lysosomal storage disease, caused by mutations in the cystinosin (*CTNS*) gene, encoding the lysosomal H^+^‐cystine symporter cystinosin [1, 2]. This leads to cystine accumulation in lysosomes in all body cells. The disease manifests in three clinical forms: infantile nephropathic cystinosis, juvenile nephropathic cystinosis and ocular nonnephropathic cystinosis. The infantile form is the most severe, characterized by the rapid onset of the renal Fanconi syndrome, a proximal tubule dysfunction leading to end‐stage kidney disease [3].

Despite renal transplantation, cysteamine treatment, and improved medical care, prolonged survival revealed systemic complications such as hypothyroidism and muscular complications [4, 5]. The latter include distal myopathy, swallowing difficulties and respiratory insufficiency, resulting in substantial loss of quality of life for people living with cystinosis. The distal myopathy presents around the second decade of life and mainly affects the muscles of the hands and progresses towards proximal muscle and lower extremities over time [6]. The incidence of distal myopathy is between 24% and 69% in people living with nephropathic cystinosis [4], with electromyography (EMG) findings showing motor unit action potentials with decreased amplitude, reduced duration and polyphasic potentials. Furthermore, spontaneous activity like fibrillations and positive sharp waves was measured [7, 8]. These EMG abnormalities were also observed in people living with cystinosis who did not show overt clinical muscle weakness [8, 9]. A recent study has identified below‐average physical performance in a full cohort of 55 individuals with cystinosis [10].

Muscle biopsies showed fibre size variability and acid‐phosphatase positive vacuoles suggesting lysosomal origin [7, 8, 9]. Cystine crystals were not observed in the muscle cells, but rather in the peri‐ and endomysium, with more than 100‐fold increased cystine content compared to healthy controls [7, 11]. Next to distal myopathy, swallowing difficulties due to pharyngeal and oesophageal muscle weakness were observed in 20%–75% of people with cystinosis [12, 13]. Symptoms associated with this complication were vomiting, gagging, slow eating and impaired speech [13]. As cystinosis patients require a large amount of oral medication, swallowing difficulties may hinder the daily intake of cysteamine, as well as supportive treatment and post renal transplantation treatment, greatly affecting their quality of life and contributing to an increased risk of morbidity and mortality [6, 14]. Restrictive respiratory insufficiency occurred in 69% of the posttransplant patients who did not receive cysteamine treatment [12, 15]. Anikster et al. showed a direct correlation between the severity of pulmonary insufficiency and the severity of the myopathy observed [15].

There have been discrepancies in the literature regarding whether cysteamine delays or prevents cystinosis myopathy. Initial studies showed that the frequency of cystinosis myopathy decreased with time on cysteamine. Indeed, the drug was first reported to lower cystine levels in the muscle and stabilize muscle weakness, and thus patients diagnosed with myopathy were considered not to be adhering to the therapy [7, 12, 16]. However, recent work showed only a limited effect of cysteamine treatment on the muscular phenotype [10]. An online survey conducted by the Adult Care Excellence Initiative (2016), including individuals with cystinosis, their parents, clinicians and researchers, highlighted swallowing difficulties and muscle weakness as pressing concerns significantly impacting the adult patient population [17]. Recent clinical cohort studies are increasingly focusing on the extrarenal phenotypes, particularly cystinosis myopathy, with the aim of improving clinical outcome measures [6, 8, 14].

Still, the underlying causes of cystinosis myopathy remain elusive. Research on cystinosis has predominantly focused on the renal phenotype, utilizing engineered kidney cell lines, patient‐derived kidney cells and CTNS knockout animal models. In these systems, CTNS deficiency has been linked to several cellular disturbances. These include increased apoptosis, disrupted energy homeostasis, lysosomal dysfunction, accumulation of autophagosomes and hyperactivation of the mTORC1 signalling pathway, evidenced by elevated phosphorylation of downstream targets [18, 19, 20, 21]. However, whether these molecular mechanisms are similarly affected in skeletal muscle due to *CTNS* deletion remains largely unexplored, representing a significant gap in our understanding of the disease's systemic impact.

In this study, we developed an isogenic cell model to study cystinosis myopathy, employing human myoblasts that can be differentiated into myotubes, mimicking the early steps in myogenesis. By combining CRISPR/Cas9 technology with a multiomics approach, we studied the effect of *CTNS* deletion in the early steps of myogenesis.

## 2 Materials and Methods

### 2.1 Ethical Approval

LHCN‐M2 immortalized human myoblasts (*pectoralis major* origin, from a 41‐year‐old male) were received from Prof. Mouly (Institut de Myologie, Paris, France) [22]. Biobank approval for this project using human bodily material was granted by UZLeuven BioBank (Approval S64794).

### 2.2 Cell Culture

LHCN‐M2 immortalized human myoblasts were cultured in one volume of medium 199 (41 150 020; Invitrogen, Merelbeke, Belgium) and four volumes of Dulbecco's modified Eagle Medium (DMEM; 61 965‐026; Invitrogen, Merelbeke, Belgium) supplemented with 20% foetal bovine serum (GibcoTM, Thermo Scientific, Dilbeek, Belgium), 25 μg/mL Fétuin (Thermo Scientific), 5 ng/mL human epidermal growth factor (PeproTech, Hamburg, Germany), 0.5 ng/mL bovine fibroblast growth factor (PeproTech), 5 μg/mL insulin (Sigma Aldrich, Overijse, Belgium), 0.2 μg/mL dexamethasone (Sigma Aldrich) and 50 μg/mL gentamycin (GibcoTM, Thermo Scientific).

For differentiation into myotubes (mt), myoblasts (mb) were seeded on well‐plates coated with 0.1% collagen I (Merck; Hoeilaart, Belgium). At confluence, cells were washed with DMEM (Invitrogen) and differentiated using DMEM with 50 μg/mL gentamicin (GibcoTM, Thermo Scientific) and 10 μg/mL insulin (Sigma Aldrich) added. Cells were grown at 37 °C and 5% (v/v) CO_2_.

### 2.3 Nanoblade Treatment

LHCN‐M2 myoblasts were seeded in a 24‐well plate (25 000 cells/well). The next day, the cells were treated with a serial dilution of Nbs. The myoblasts were further propagated to T175 culture flasks.

### 2.4 Generation of Lentiviral Vector Transfer Plasmids for CTNS^WT^

The human CTNS^WT^ and dATP13A2 encoding cDNAs were cloned in the self‐inactivating lentiviral (SIN‐LV) backbone plasmid pCH‐promoter‐X‐IRES‐PuroR‐WPRE (Didier Trono) using Gblocks at the BcuI and Bsp119I4 restriction sites. The cDNA construct encoding dATP13A2 (D508N; a catalytically dead version of ATP13A2) was used as transduction controls and to control for overexpression [23]. The *CTNS* ^*WT*^ cDNA was tagged with a triple hemagglutinin tag sequence (*3HA* tag) at its 3′‐end.

### 2.5 Protein Analysis by PAGE and Western Blot

Myoblasts (50 000 cells/cm^2^) were differentiated over a 4‐day period in collagen I‐coated 6‐well plates. For the BafA1 condition, 100 nM Bafilomycine A1 (BafA1) (Santa Cruz) was added to the differentiation medium for 4 h prior to sample collection. PAGE and Western blot analysis were performed as previously described [24]. The specification of all antibodies is described in *Supplementary Table* *S2*. Images were acquired using Amersham ImageQuant 800 Fluor (GE Healthcare, Diegem, Belgium) and subsequently quantified with ImageQuantTM TL software.

### 2.6 Statistical Analysis

Unless otherwise indicated, data are plotted in figures as the mean ± standard deviation (SD) with individual data points shown in each group (replicates of multiple independent experiments). Confidence intervals (CIs) of either means or medians were calculated using the *t* test. GraphPad 10.4.2 was used to plot all graphs and perform statistical analysis. Unless otherwise indicated, multiple hypothesis testing was executed using the Benjamini–Hochberg procedure for false discovery rate (FDR, *q* value).

## 3 Results

### 3.1 A Polyclonal, Isogenic Human Myoblast Model Through CRISPR/Cas9 Editing of CTNS

**FIGURE 1:**
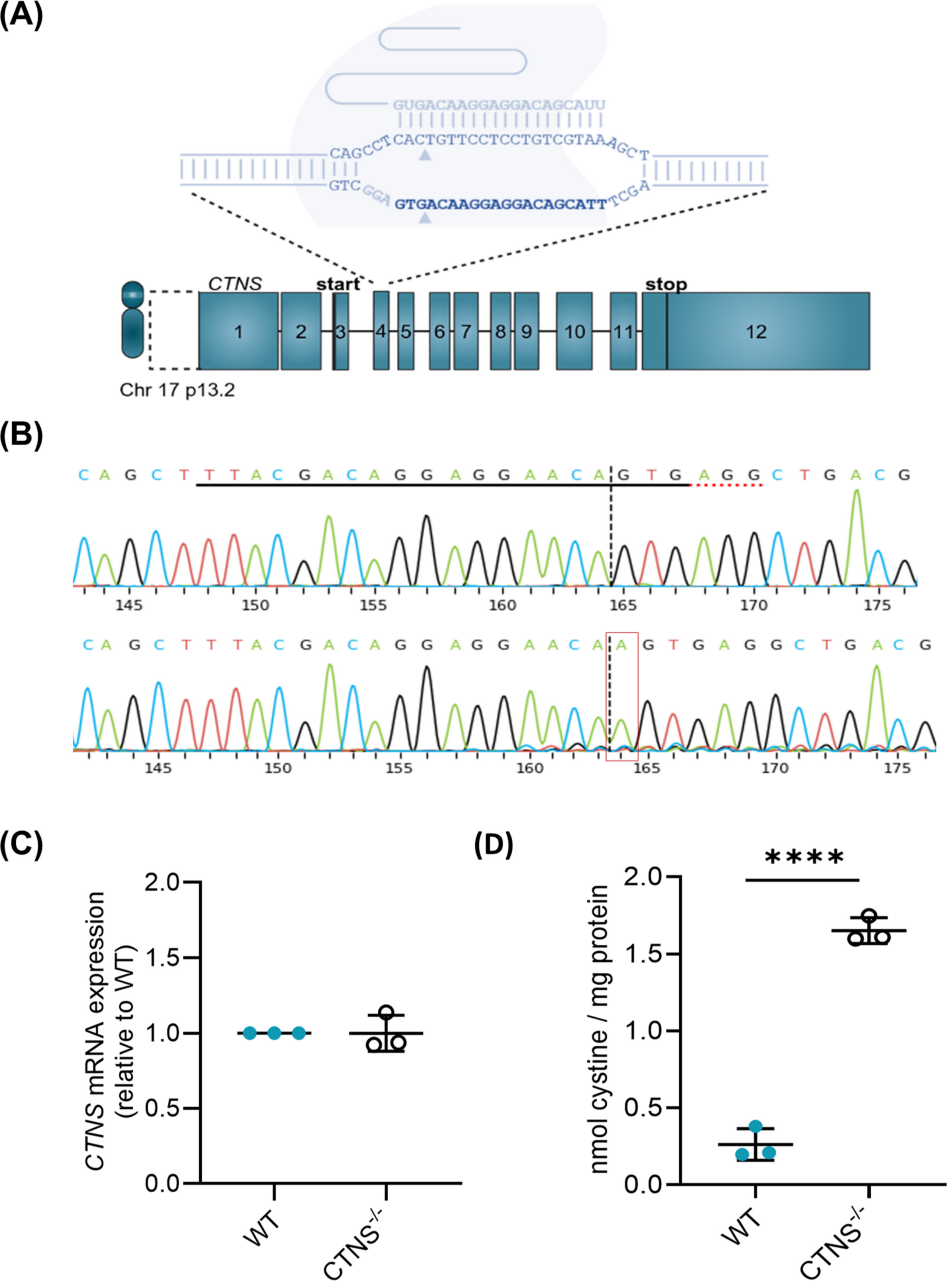
**Development of a polyclonal isogenic human muscle cell model for cystinosis myopathy. (A)** Set‐up of the use of CRISPR/Cas9 to knock‐out the CTNS gene at exon 4 in human immortalized LHCN‐M2 myoblasts. **(B)** Quantification of indel events in CRISPR‐edited CTNS^−/−^ myoblasts at gDNA level using ICE analysis. gRNA and PAM site are indicated with a solid black and dashed red line, respectively. Analysis is shown on reverse strand 5′➔ 3′. **(C)** CTNS mRNA expression analysis (RT‐qPCR) on WT and CTNS^−/−^ myoblasts. The data are normalized for total mRNA levels of ACTG and are presented as the mean ± SD (*n* = 3). **(D)** Cystine measurement by mass spectrometry in WT and CTNS^−/−^ myoblasts. The graph shows mean ± SD (*n* = 3 independent metabolite extracts). Statistics was performed by unpaired *t* test (*p* ≤ 0.0001; ****). Chr, chromosome; WT, wild type.

To understand the effect of CTNS loss on muscle development, we created an isogenic CTNS knockout cell line (*CTNS* ^*−/−*^) by knocking out the *CTNS* gene in the human LHCN‐M2 myoblast cell line [22]. A gRNA targeting exon 4, the second coding exon of *CTNS*, was used to introduce indels by CRISPR/Cas9 using nanoblade technology (Figure 1A) [25]. After nanoblade treatment, a 280‐bp gDNA fragment flanking the cut site was amplified and subsequently DECODR analysis was performed to determine the editing efficiency. The polyclonal KO cell line showed a > 95% frame shift, resulting in an early stop codon and suggesting loss of CTNS protein (Figure 1B *Supplementary Figure* *S1*A). The transient delivery of the sgRNA/Cas9 complex in the myoblasts decreases the chance of off‐target edits [25]. As an additional control, three computationally predicted off‐target sites with the highest scores were selected and assessed, but no indels were detected (*Supplementary Table* *S4*).

To further corroborate our KO, the *CTNS* mRNA levels were measured. No significant alterations in *CTNS* mRNA levels were shown compared to control‐treated wild‐type cells (WT) (Figure 1C). Sequencing of a 467‐bp cDNA fragment corroborated the full translational frameshift (stop codon in exon 5, predicted length protein 58 amino acids [AAs]; *Supplementary Figure* *S1* *B*). Functional *CTNS* KO was corroborated by a 6‐fold increase in cystine concentration in *CTNS* ^*−/−*^ myoblasts compared to WT myoblasts (Figure 1D).

### 3.2 Study of the Effect of *CTNS* Deletion on Myogenesis

**FIGURE 2:**
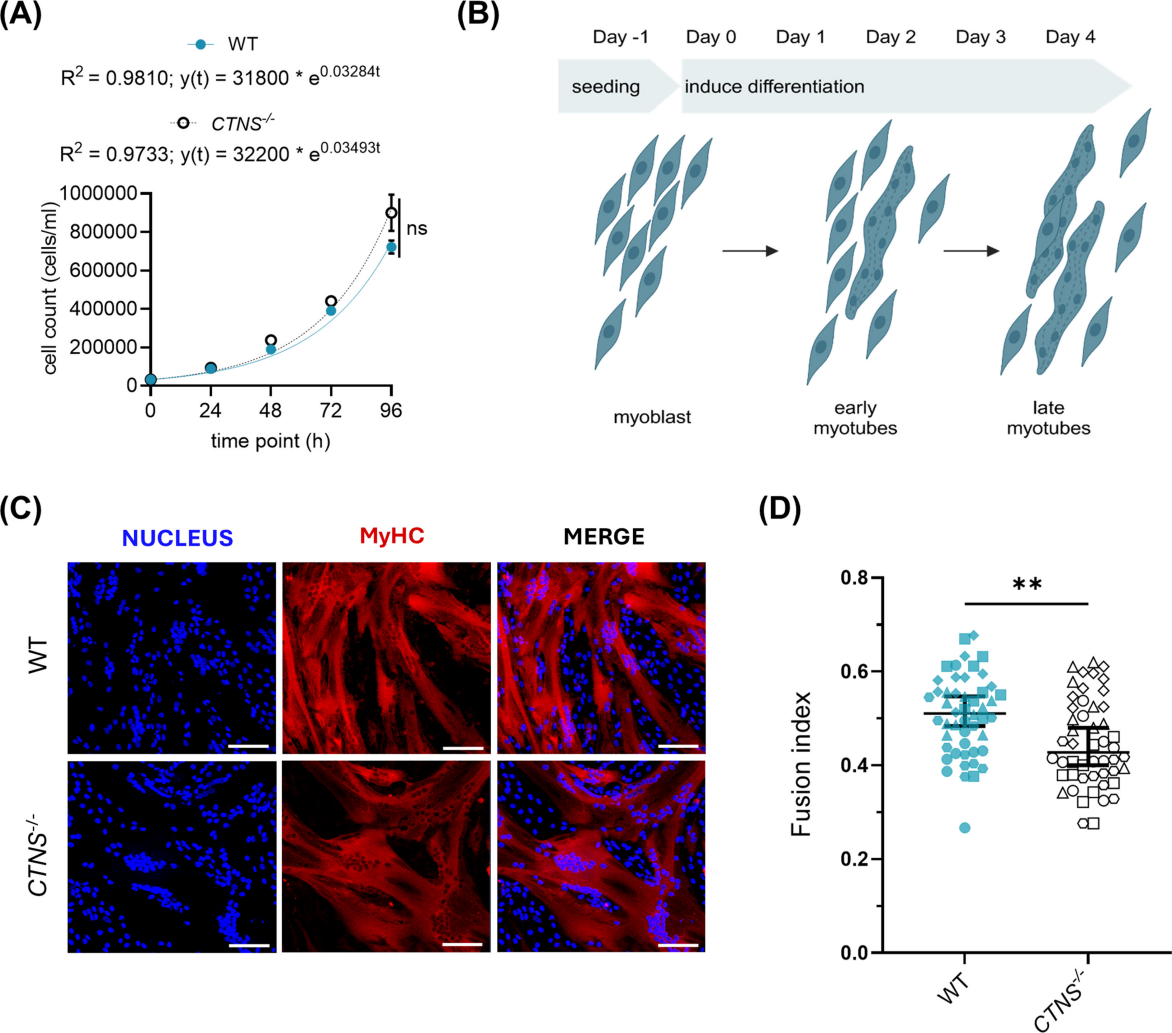
**Functional validation of the growth and fusion potential of CTNS** ^**−/−**^ **myoblasts. (A)** Growth was quantified as the number of myoblasts at 0, 24, 48, 72 and 96 h post seeding. All data are expressed as mean ± SD (*n* = 3), *R* ^2^ represents R‐squared value. The growth rate was calculated by fitting the number of cells (y‐axis) at different times (x‐axis) to an exponential growth curve using *y*(*t*) = *a* × *e* ^Δ*t*^ where ‘a’ is the number of cells at 0 h. Ns, nonsignificant, *p* > 0.05. Statistical testing was performed with a two‐way ANOVA, Bonferroni's multiple comparison test. **(B)** Schematic representation of differentiation set‐up from myoblast to multinucleated myotubes. **(C)** Representative immunofluorescence images from WT and CTNS^−/−^ myotubes after 4 days of myogenic differentiation. Staining by nucleus (DAPI, blue), MyHC (red) and merged. Scale bar: 100 μm. **(D)** Fusion index is represented by scatter dot plots with each dot representing an individual image field. This graph shows the median with 95% CI of *n* = 5 time‐independent myogenic differentiations (10 ROIs per replicate, plotted in different shapes); **p < 0.01; statistical testing was performed with an unpaired *t* test with Welch's correction.

To identify the effects of *CTNS* KO on the early steps in muscle development, myoblast proliferation and differentiation capacity were determined. Cell proliferation was determined by setting up a growth curve and calculating the doubling time of *CTNS* ^*−/−*^ myoblasts, which was in line with the WT profile (Figure 2A). The myogenic differentiation capacity was determined over a 4‐day period following serum starvation (Figure 2B). Different parameters of myogenic differentiation were quantified using the Myotube Analyzer tool [26]. The fusion index was mildly but significantly altered because of CTNS loss, being 51 ± 3% for WT and 43 ± 4% for CTNS^−/−^ myotubes (95% CI of median, *p* < 0.01; Figure 2C,D *, Supplementary Figure* *S2* *A,B*). In line, the fraction of covered area by myotubes was also significantly altered, but no differences were observed in branching or number of myotubes (*Supplementary Figure* *S2* *C,D*).

In the next step, we explored the levels of a selection of key proteins involved in muscle differentiation and function, such as MyHC, dystrophin and RyR via Western blot analysis. MyHC is a structural protein critical for sarcomere formation, muscle contraction and muscle fibre differentiation [27]. Conventional myosins are constituted by light chains and different MyHC isoforms (recognized by the antibody MF20 used), which grant them specialized muscle functions [28]. On the other hand, RyR is intracellular Ca^2+^ channels essential for initiating muscle contraction, and although the antibody used here recognizes all three RyR isoforms, in skeletal muscle we expect mainly RyR1 and RyR3 [29].

**FIGURE 3:**
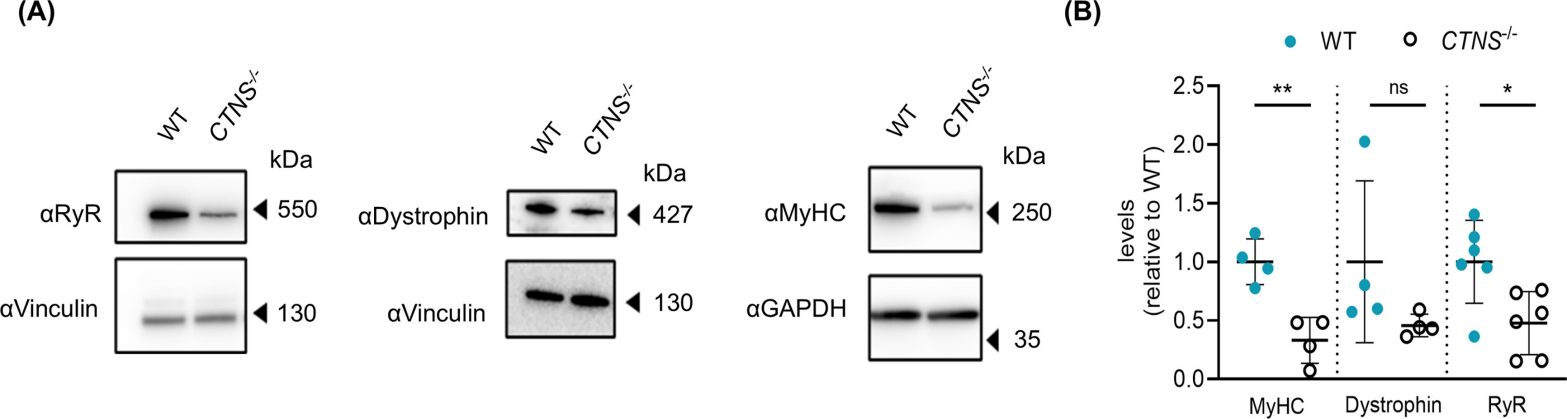
**Reduced levels of MyHC and RyRs isoforms upon CTNS deletion in myotubes. (A)** Representative western blot analysis of RyRs, MyHC and dystrophin protein signals in WT and CTNS^−/−^ 4‐day differentiated myotubes. Samples normalized for total proteins of vinculin or GAPDH. **(B)** Quantification of MyHC, dystrophin and RyR protein expression levels in WT and CTNS^−/−^ 4‐day differentiated myotubes. Samples were normalized for total proteins of vinculin or GAPDH. The data are presented as the mean ± SD (*n* = 4–6 independent experiments). Statistical testing was performed with an unpaired *t* test. *p < 0.05; **p < 0.01; ns, nonsignificant, *p* > 0.05.

*CTNS* ^*−/−*^ myotubes had significantly lower levels of MyHC and RyR compared to WT myotubes (Figure 3), whereas dystrophin levels, a cytoskeletal protein crucial for sarcolemmal integrity, were nonsignificantly altered [30]. Housekeeping proteins vinculin and GAPDH, on the other hand, remained unaltered. In addition, no statistical differences were found for downstream targets of the mTOR pathway (p‐S6 and p70S6 kinase) at basal conditions and after starvation (*Supplementary Figure* *S3* *A,B*). These findings suggested that the loss of *CTNS* depleted the levels of key proteins of muscle differentiation and mildly lowered the fusion capacity of myoblasts to myotubes.

### 3.3 Cytosolic Ca^2+^ Handling Remains Unaffected in *CTNS* ^*−/−*^ Myotubes

Ca^2+^ plays a crucial role in muscle function as it initiates muscle contraction and relaxation. With RyR being affected in *CTNS* ^*−/−*^ myotubes, and with RyR1 and RyR3 playing a pivotal role in Ca^2+^ concentration in skeletal muscle cells, we set out to assess the impact of decreased RyR levels on cytosolic Ca^2+^ signalling. Ca^2+^ imaging was performed in single myotubes using Cal520 as a Ca^2+^ indicator (*Supplementary Figure* *S4*). Upon the addition of different acetylcholine (Ach) concentrations, an endogenously relevant agonist for skeletal muscle activation, similar Ca^2+^ transients were observed in *CTNS* ^*−/−*^ and WT myotubes, indicating that the Ca^2+^ signalling in Day 4 *CTNS* ^*−/−*^ myotubes was not significantly altered.

### 3.4 Lysosomal Function of *CTNS* ^*−/−*^ Myotubes Remains Unaffected

**FIGURE 4:**
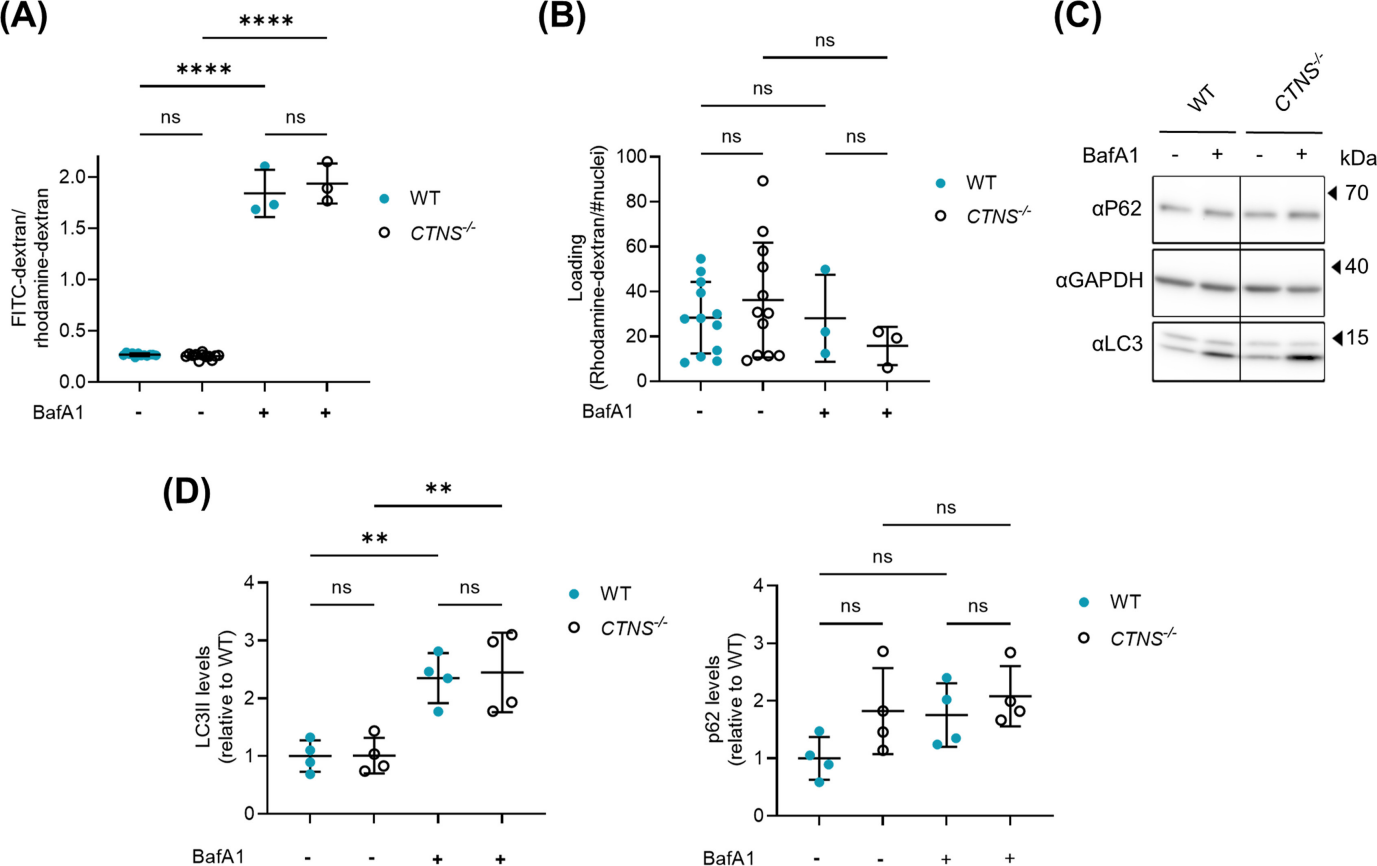
Validation of lysosomal function of CTNS^−/−^ myotubes. **(A)** Lysosomal pH measurement in WT and CTNS^−/−^ 4‐day differentiated myotubes. Intracellular levels of pH‐sensitive fluorescently labelled dextran were determined 24 h after uptake. BafA1 (100 nM) was used as a control. Data are presented as the mean ± SD (*n* = 3 independent experiments, with 5 replicates for which one was used as BafA1 control). Statistical testing was performed with a one‐way ANOVA, Sidak's multiple comparison test. **(B)** Loading control for pH measurement WT and CTNS^−/−^ 4‐day differentiated myotubes. Loading was quantified as intracellular levels of rhodamine labelled dextran over total number of nuclei 24 h after uptake. BafA1 (100 nM) was used as a control. Data are presented as the mean ± SD (*n* = 3). **(C)** Western blot analysis of LC3 and p62 protein expression in WT and CTNS^−/−^ 4‐day differentiated myotubes. Samples normalized for total proteins of GAPDH. BafA1 (4 h—100 nM) was used to measure autophagic flux. **(D)** Quantification of LC3II and p62 protein expression in WT and CTNS^−/−^ 4‐day differentiated myotubes (*n* = 4 independent experiments). Samples were normalized for total proteins of vinculin or GAPDH. Statistical testing was performed with a one‐way ANOVA, Sidak's multiple comparison test. BafA1, bafilomycin A1; FITC, fluorescein isothiocyanate; ****p < 0.0001; **p < 0.01; ns, nonsignificant, *p* > 0.05.

Since CTNS is a lysosomal membrane protein, its deletion could disrupt lysosomal function. Therefore, we examined lysosomal pH and autophagic flux to assess lysosomal functionality. Lysosomal pH was estimated by the ratio of lysosomal fluorescence intensity of endocytosed fluorescein (FITC)‐dextran and rhodamine‐dextran. FITC fluorescence increases at higher pH, whereas rhodamine fluorescence is pH independent, thus rendering the fluorescein/rhodamine ratio pH‐dependent, allowing normalization for dye uptake. BafA1 was used as a positive control as it inhibits v‐ATPase, thereby promoting alkalinisation of the lysosomal lumen and blocking lysosomal fusion. The lysosomal pH (FITC/rhodamine fluorescence intensity ratio) remained unaltered in *CTNS* ^*−/−*^ myotubes compared to WT, while BafA1 increased the lysosomal pH in both conditions (Figure 4A). Additionally, we found no statistical difference in the ratio of rhodamine fluorescence/# nuclei, indicating that there were no significant alterations in lysosomal uptake via endocytosis in *CTNS* ^*−/−*^ myotubes (Figure 4B).

Lysosomes regulate cellular processes, including autophagy, and LC3 and p62/SQSTM1 have been widely used to monitor autophagic flux [31]. It has previously been reported that the loss of CTNS is associated with disrupted autophagy dynamics in cystinosis PTECs and fibroblasts [18]. In our model, LC3II levels were similar at baseline and upon addition of BafA1 for WT and *CTNS* ^*−/−*^, indicating comparable autophagic flux (Figure 4C,D). For p62, the results were more variable, but no significant alterations were observed in *CTNS* ^*−/−*^ compared to WT (*p* = 0.23 without BafA1 and *p* = 0.89 upon addition of BafA1; Figure 4C,D). Taken together, these findings revealed no overt changes in autophagy in this *CTNS* ^*−/−*^ myoblast cell line during early steps of myogenic differentiation to myotubes.

### 3.5 CTNS^WT^‐3HA cDNA Addition Restores Cystine Levels, Fusion Index and RyR and MyHC Protein Levels in CTNS^−/−^ Myotubes

**FIGURE 5:**
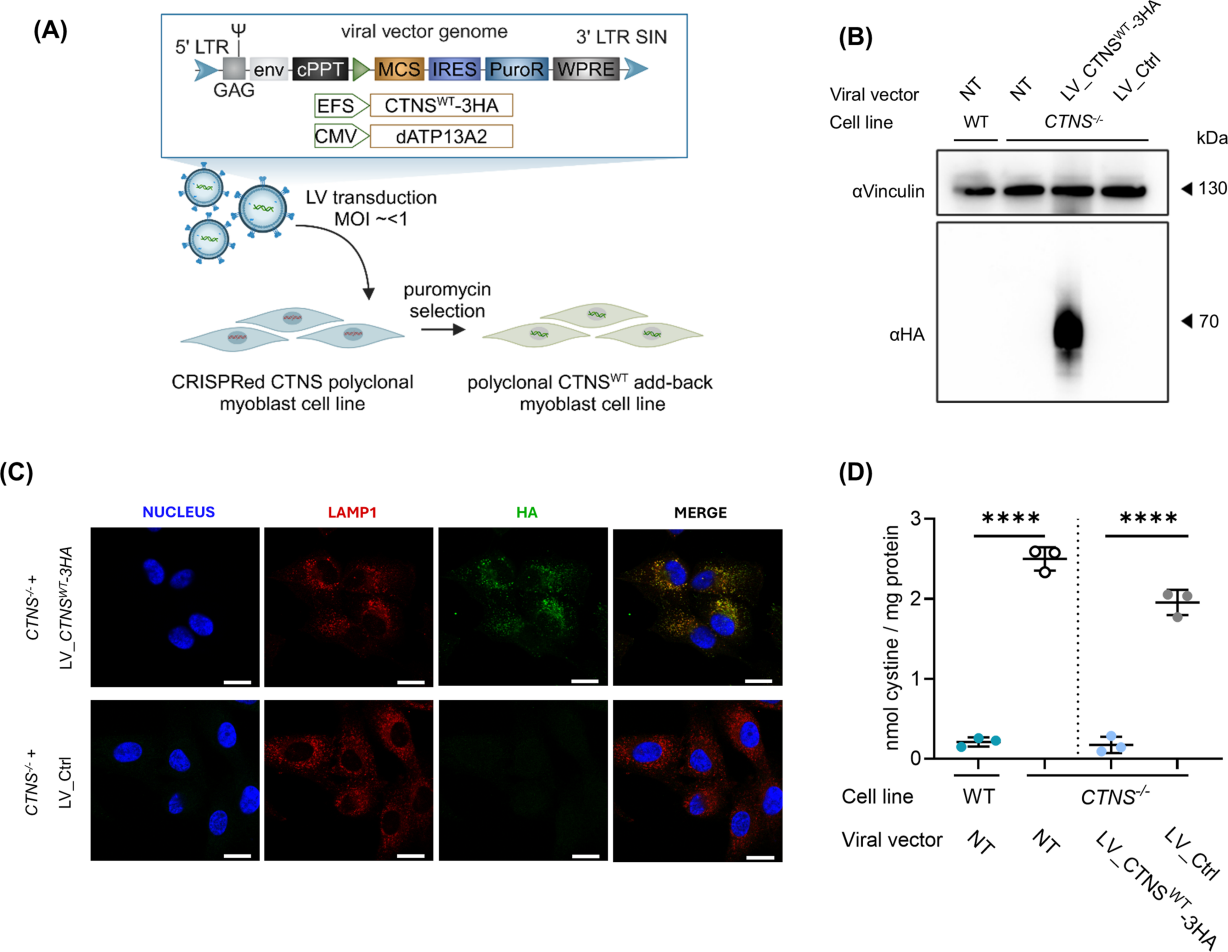
**Cystine accumulation decreases upon CTNS** ^**WT**^ **cDNA addition in CTNS** ^**−/−**^ **myotubes. (A)** Schematic representation of lentiviral transfer plasmid set‐up encoding CTNS^WT^‐3HA cDNA used to produce lentiviral vectors, and their addition to CTNS^−/−^ myoblasts. **(B)** Western blot analysis of CTNS‐3HA protein expression in CTNS^−/−^ myoblasts transduced with lentiviral vectors LV_CTNS^WT^‐3HA or LV_Ctrl. Samples normalized for total proteins of vinculin. **(C)** Representative confocal microscopy images of the immunofluorescence signal for nucleus (DAPI, blue signal), CTNS‐3HA (HA, green signal) and LAMP1 (red signal) in CTNS^−/−^ myoblasts transduced with either LV_ CTNS^WT^‐3HA or LV_Ctrl. Scale bar: 20 μm. **(D)** Cystine measurement (mass spectrometry) of Day 4 myotubes (WT, CTNS^−/−^ and CTNS^−/−^ complemented with either LV_CTNS^WT^‐3HA or LV_Ctrl). The data are presented as the mean ± SD (*n* = 3 independent metabolite extracts). Statistical testing was performed with a one‐way ANOVA, Sidak's multiple comparison test. cPPT, central polypurine tract; LTR, long terminal repeats; LV, lentiviral vector; LV_Ctrl, LV_pCMV‐dATP13A2; IRES, internal ribosomal entry site; MCS, multiple cloning site; PuroR, puromycine resistance cassette; SIN, self‐inactivating; WPRE, woodchuck hepatitis virus posttranscriptional regulatory element; WT, wild‐type; ****p < 0.0001.

To corroborate that the observed phenotypes of lower fusion index and lower RyR and MyHC levels in myotubes were specific to *CTNS* deletion, we set out to assess the effect of *CTNS* ^*WT*^ cDNA addition. *CTNS* ^*−/−*^ myoblasts were complemented with *CTNS* ^*WT*^ *‐3HA* cDNA using LVs (LV_*CTNS* ^*WT*^ *‐3HA*; Figure 5A). As a control for lysosomal expression, *CTNS* ^*−/−*^ myoblasts were complemented with a LV encoding the catalytically dead ATP13A2 (dATP13A2) protein (LV_Ctrl), a lysosomal transmembrane protein [32].

CTNS^WT^‐3HA protein expression was confirmed by western blot analysis and immunocytochemistry, showing subcellular co‐localisation with lysosomal associated membrane protein 1 (LAMP1; Figure 5B,C). Additionally, *CTNS* ^*WT*^ cDNA addition significantly reduced cystine levels in CTNS^−/−^ Day 4 myotubes (0.17 ± 0.24 nmol cystine/mg protein; 95% CI of mean) compared to transduction control (1.95 ± 0.40 nmol cystine/mg protein; 95% CI of mean), rescuing cystine accumulation to a similar concentration of WT myotubes (0.21 ± 0.14 nmol cystine/mg protein; 95% CI of mean; Figure 5D).

**FIGURE 6:**
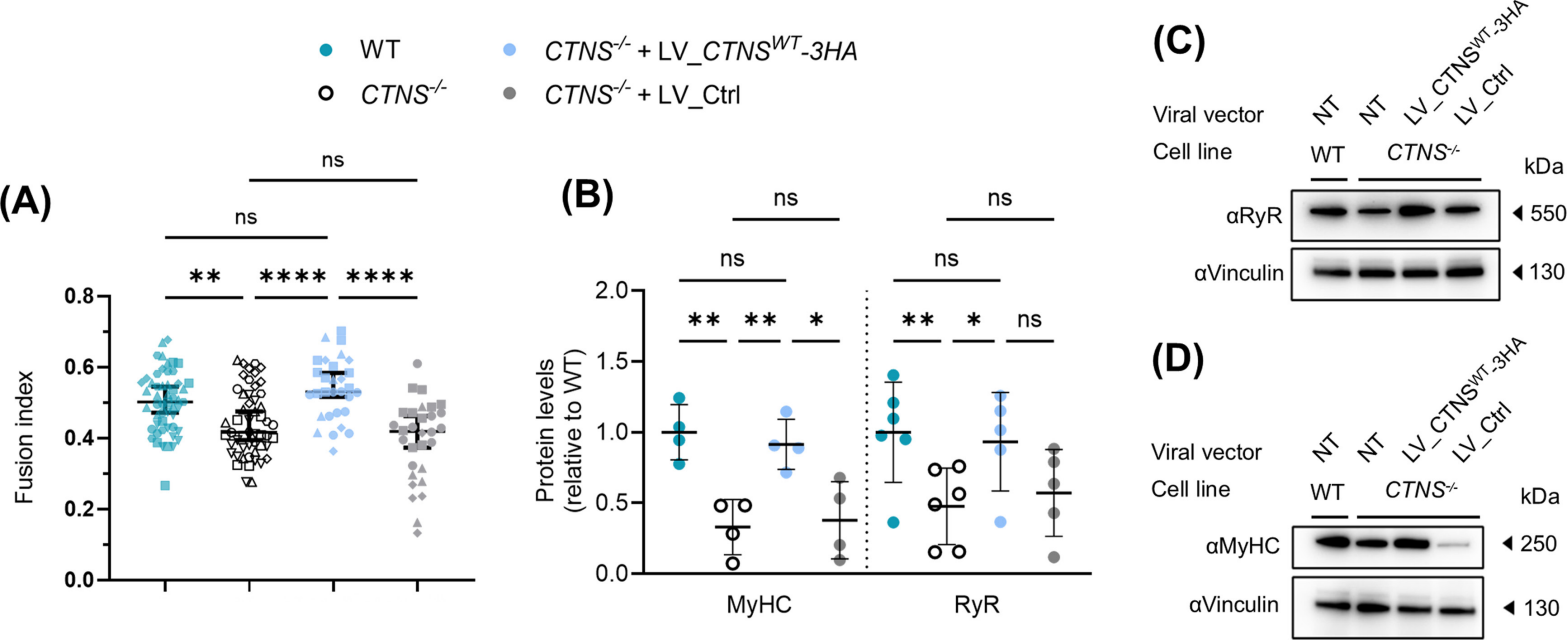
**Rescue of fusion index and MyHC and RyR levels upon CTNS** ^**WT**^ **cDNA addition to CTNS** ^**−/−**^ **myoblasts. (A)** Fusion index of WT, CTNS^−/−^ and CTNS^−/−^ complemented with either LV_CTNS^WT^‐3HA or LV_Ctrl represented by scatter dot plots with each dot representing an individual image field. The data for WT and CTNS^−/−^ are the same datapoints as in Figure 2D. Data are presented as median with 95% CI of *n* = 5 (for WT and CTNS^−/−^) and *n* = 3 (for CTNS^−/−^ + LV_CTNS^WT^‐3HA/LV_Ctrl) independent myogenic differentiations (10 ROIs per replicate, plotted in different shapes). **(B)** Quantification of the RyR and MyHC protein levels in WT, CTNS^−/−^ and CTNS^−/−^ complemented with either LV_CTNS^WT^‐3HA or LV_Ctrl, in 4‐day differentiated myotubes. Samples were normalized to vinculin or GAPDH levels. Data shown are depicted relatively to the average of the WT levels. The data for WT and CTNS^−/−^ are the same datapoints as in Figure 3. The data are presented as the mean ± SD (*n* = 4–5). **(C)** Western blots of RyR and **(D)** MyHC, protein expression in WT, CTNS^−/−^ and CTNS^−/−^ complemented with either LV_CTNS^WT^‐3HA or LV_Ctrl, 4‐day differentiated myotubes. All statistical testing was performed with one‐way ANOVA, Sidak's multiple comparison test. LV, lentiviral vector; WT, wild‐type; *p < 0.05; **p < 0.01; ***p < 0.001; ns, nonsignificant, *p* > 0.05.

In a next step, we corroborated that *CTNS* ^*WT*^ cDNA reintroduction rescued the mildly affected fusion index of Day 4 CTNS^−/−^ myotubes (from 43 ± 4% to 53 ± 3%; 95% CI of median, *p* < 0.001), whereas in the control addback (LV_Ctrl), it stayed at 42 ± 4% (95% CI of median; Figure 6A). In line, the fraction of covered area by myotubes was also significantly rescued (*Supplementary Figure* *S5* *A,B*). In addition, the decreased RyR and MyHC protein levels in Day 4 *CTNS* ^*−/−*^ myotubes were also reverted upon *CTNS* ^*WT*^ cDNA reintroduction (Figure 6B–D), whereas the other proteins remained constant throughout (vinculin, GAPDH, dystrophin; not shown).

### 3.6 *CTNS* Deletion Causes the Depletion of a Protein Network Involved in Myofibril Assembly

Considering the western blot results indicating that *CTNS* KO caused a significant decrease in key proteins involved in muscle cell differentiation (MyHC, RyR), we set out to explore the *CTNS* deletion effect using unbiased quantitative proteomic profiling of myoblasts and Day 4 differentiated myotubes.

Using diaPASEF, more than 8000 protein groups were quantified. Firstly, we compared Day 0 with Day 4 of the differentiation process for both WT and *CTNS* ^*−/−*^ cells separately, observing a statistically significant change upon differentiation (FDR adjusted *p* < 0.05) in more than 4000 protein groups, 77% of them being shared between WT and *CTNS* ^*−/−*^ cells (*Supplementary Figure* *S5* *C,D*). In accordance, in both the WT and the *CTNS* ^*−/−*^ conditions, there was a decrease of myoblast specific markers and an increase in myotube specific proteins, underscoring the differentiation of myoblasts into myotubes (*Supplementary Figure* *S5* *E*).

**FIGURE 7:**
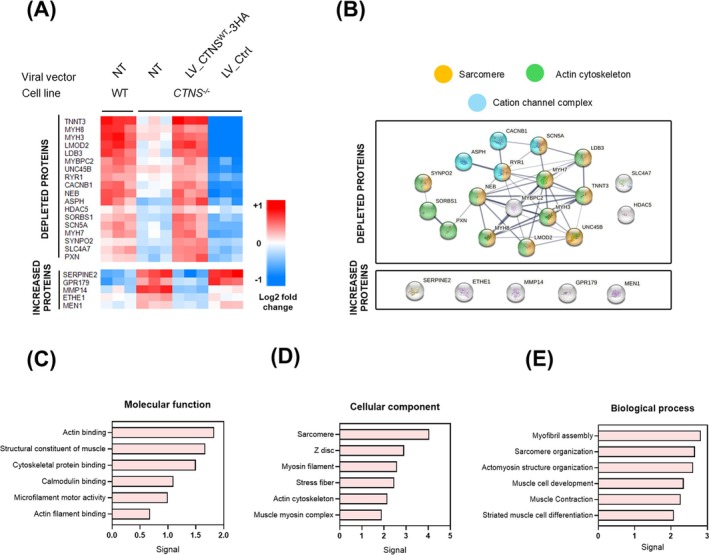
**Selected protein groups modified in CTNS** ^**−/−**^ **myotubes and are rescued to WT levels upon CTNS‐3HA cDNA addback. (A)** Heatmap of protein group levels altered in CTNS KO conditions and restored upon CTNS^WT^ cDNA addback, divided into 18 depleted protein groups (on top) and 5 increased protein groups (bottom), n = 3. **(B)** STRING protein–protein interaction network of identified depleted and increased protein networks. Colours represent clusters of cellular components, in white, protein groups that did not cluster. Line thickness indicates the strength of data support. **(C)** Proteomic analysis of depleted protein network related to Gene Ontology Molecular Function, **(D)** to Gene Ontology Cellular Component and **(E)** to Gene Ontology Biological Process. Signal corresponds to the weighted harmonic mean between the observed/expected ratio and ‐log (FDR), obtained from https://string‐db.org/.

Differential analysis of WT and *CTNS* ^*−/−*^ myoblasts or Day 4 myotubes learned that in myoblasts no protein groups were identified to be specifically influenced by *CTNS* deletion (*p* < 0.01). On the other hand, in Day 4 myotubes, we identified a total of 23 affected (*p* < 0.01) protein groups that were restored to WT levels upon *CTNS* addback (LV_*CTNS* ^*WT*^ *‐3HA*) to *CTNS* ^*−/−*^ and not by LV_Ctrl, from which 18 were depleted and five were increased in CTNS deletion conditions (Figure 7A).

After applying a multiple hypothesis correction using the Benjamin–Hochberg test, none of these differential protein groups showed a *q* value below 0.05 (*Supplementary Figure* *S6* *A,B*). The lack of statistical significance after multiple hypothesis correction is a common challenge in high‐dimensional omics data, where biologically meaningful changes may not always reach statistical thresholds due to sample size or effect size limitations (Type II error). On the other hand, the use of unadjusted *p* values can lead to false positives (Type I error) [33].

Consequently, we selected the affected protein groups using unadjusted *p* values taking only into account protein groups that were not only affected by *CTNS* KO (*p* < 0.01) but that additionally were rescued to WT levels after *CTNS* ^*WT*^ *‐3HA* cDNA addback (*p* < 0.01) and not by the control addback (LV_Ctrl, *p* < 0.01). The fact that in myoblasts no protein groups were identified to be specifically altered underscores the solidity of our strategy.

Although the five increased protein groups were not biologically related (STRING protein–protein interaction (PPI) enrichment *p* = 0.559; Figure 7B), these protein groups (except for GPR179) are linked to oxidative stress pathways [34, 35, 36, 37]. Relevantly, in the depleted proteins, RyR1 as well as three different isoforms of MyHC (MYH3, MYH7, MYH8) were identified, with profiles of abundance changes that resembled the results seen in Figure 6C,D western blots (*Supplementary Figure* *S7*). Moreover, the 18 depleted protein groups were biologically connected with a PPI enrichment *p* < 1.0e‐16 (FDR adjusted; Figure 7B), sharing molecular functions related to actin binding and structural constituent of muscle (Figure 7C), as well as sharing a cellular component annotation related to myofibrils (Figure 7D). Concurrently, the depleted groups of proteins due to *CTNS* KO clustered in the biological processes of myofibril assembly, sarcomere organization, muscle cell development and differentiation (Figure 7E). Moreover, alterations in this network of proteins are related to abnormalities in the musculoskeletal system (*Supplementary Figure* *S8*).

Interestingly, all 18 depleted proteins were myotube specific, being underrepresented or absent in myoblasts and increased during myotube development at Day 4 myotubes more abundantly in WT compared to *CTNS* ^*−/−*^ myotubes (*Supplementary Figure* *S9*).

### 3.7 Depleted Protein Transcripts Show Alteration Trend due to CTNS Loss

**FIGURE 8:**
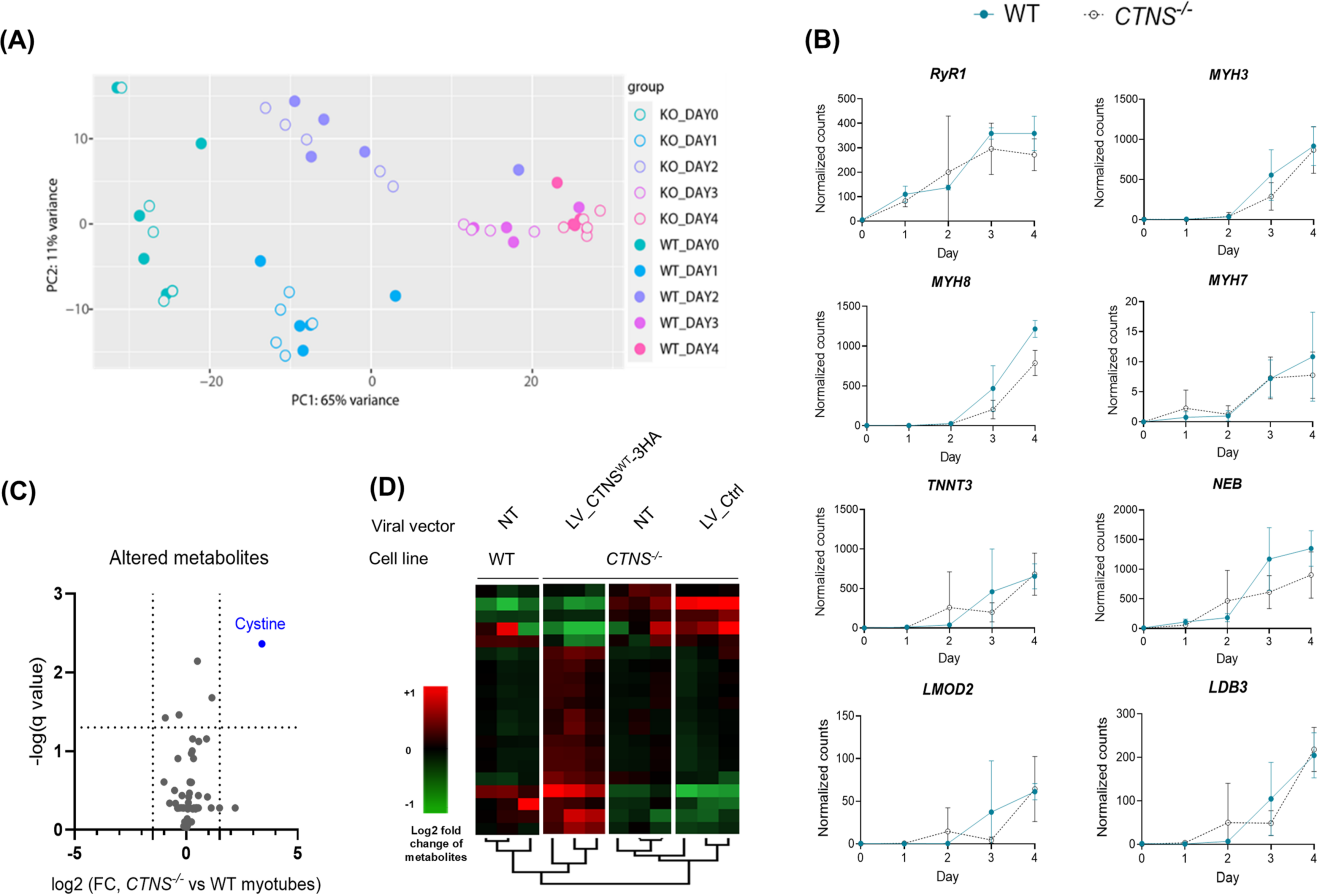
**Metabolomics analysis and temporal transcriptome analysis of modified protein groups. (A)** PCA summarizing temporal dynamics of global transcriptome changes comparing WT and CTNS^−/−^ (KO) myoblasts during 4 days of differentiation. **(B)** Normalized counts of mRNA expression levels of WT and CTNS^−/−^ myoblasts during the 4 days of differentiation for the depleted proteins functionally enriched in the biological processes of muscle cell differentiation and/or skeletal muscle contraction (RyR1, MYH3, MYH7, MYH8, TNNT3. NEB, LDB3, LMOD2). The mRNA expression plots of the rest of the affected protein groups can be found in Supplementary Figure 10D, E. Data are presented as the mean ± SD *n* = 4. **(C)** Volcano plot of metabolome differences and statistical relevance between WT and CTNS^−/−^ Day 4 myotubes. Thresholds correspond to a *q* value < 0.05 and a |log2FC| > 1.5; *n* = 3 independent metabolite extracts. **(D)** Heat map analysis of top 20 metabolites distinctively expressed in WT and CTNS^−/−^ myotubes without including cystine. Colour scale represents log2 fold change from average metabolite levels. Complete metabolome clustering can be found in Supplementary Figure 10F. *n* = 3 independent metabolite extracts.

We conducted bulk RNAseq transcriptome profiling to investigate temporal expression of the protein genes between WT and *CTNS* ^*−/−*^ conditions at every day of the differentiation protocol, from proliferating myoblasts (Day 0) to 4‐day differentiated myotubes. In total, 24 631 different transcribed loci were identified across the conditions. PCA showed that both WT and *CTNS* ^*−/−*^ samples clustered within temporal dynamics during myogenic differentiation (Figure 8A). Of note, downregulation of myoblast‐specific marker *MYF5* confirmed the differentiation of myoblasts (*Supplementary Figure* *S10* *A*).

Although the gene expression between WT and *CTNS* ^*−/−*^ for the 23 selected proteins was statistically nonsignificant (*q* > 0.05), for most of the depleted protein groups, the mRNA level showed a trend of being downregulated in *CTNS* ^*−/−*^ (Figure 8B,C *, Supplementary Figure* *S10* *B–E*). Notably, in accordance with protein level results, the genes of the depleted proteins were not expressed in myoblasts and were significantly upregulated upon differentiation. This profile was observed for all the genes encoding the depleted proteins, except in *SLC4A7*, *HDAC5*, *PXN* and *ASPH*, which encoded proteins biologically less related in the depleted protein network (Figure 7B).

### 3.8 CTNS^WT^ Addback Restores Metabolome Signature of *CTNS* ^−/−^ Myotubes

One of the possible explanations for the affected protein groups in *CTNS* ^*−/−*^ myotubes could be metabolic imbalance, which has been characterized in proximal tubule cells due to *CTNS* deletion [20, 21]. We performed metabolic analysis in Day 4 myotubes (WT, *CTNS* ^*−/−*^ and LV‐gene addback conditions), identifying 53 different metabolites besides cystine.

Cystine was the only metabolite significantly altered (*q* < 0.05; |log2FC| > 1.5; Figure 8C). Nevertheless, unsupervised hierarchical clustering showed that the metabolome, even without including cystine, of CTNS^−/−^ myotubes complemented with LV_CTNS^WT^‐3HA was more similar to WT than to CTNS^−/−^ myotubes (Figure 8D, *Supplementary Figure* *S10* *F*). In line, the conditions with lack of functional CTNS also clustered together (*CTNS* ^*−/−*^ with *CTNS* ^*−/−*^ complemented with LV_Ctrl).

## 4 Discussion

Cystinosis myopathy typically emerges in adolescence or adulthood, affecting 25 to 70% of adults living with cystinosis. It usually begins with hand weakness and progresses to limb muscle impairments, swallowing and speech difficulties and pulmonary insufficiency. Little is known about the aetiology of cystinosis myopathy, with limited and outdated studies available [7, 38]. Cysteine crystal deposits have been identified in the perimysium, the connective tissue surrounding muscle fibres, as well as in the cytoplasm of resident macrophages [7, 38]. However, it is unclear why cystine crystals fail to form in muscle fibres as seen in other tissues. It is also unknown whether cystinosis myopathy results from intrinsic defects at the myoblast or myotube level, dysfunctions at the neuromuscular junction, neuronal impairments or a combination of these factors. Similarly, it is not known whether it is the lack of CTNS protein, the accumulation of cystine or their combination that derails muscular function. With most of the cystinosis research being focused on kidney disease, there is a contemporary knowledge gap in the molecular mechanisms underlying cystinosis myopathy, as well as a lack of cellular models that can provide further insight into the muscle phenotype.

We generated an isogenic human myoblast‐based model to advance the understanding of the underlying molecular mechanism of cystinosis myopathy. The resulting isogenic model was polyclonal following transient treatment with CRISPR/Cas containing virus‐like particles (nanoblades) targeting the *CTNS* gene. This resulted in a reading frameshift and a premature stop codon after 58 AA, and the absence of functional CTNS protein led to significantly elevated cystine levels, consistent with findings from muscle biopsies and patient‐derived myoblasts [7, 11].

CTNS deletion and consequent cystine accumulation did not impact myoblast proliferation but mildly affected the capacity of myotube formation. The only report of cystinosis impact on myotube development is an old study by Harper et al., which observed that myoblasts from people with cystinosis could fuse in a qualitatively normal fashion, but failed to provide any quantitative measurement [11]. The mildly affected fusion index we observed in CTNS^−/−^ myotubes is consistent with the mild myopathy and the below‐average muscular performance of individuals with cystinosis [10].

Loss of CTNS function prevented cystine mobilization from the lysosomes, yet autophagy and lysosomal function appeared unaffected. In addition, we did not detect significant alterations in mTORC1 activity in *CTNS* ^*−/−*^ myotubes. Defects in this pathway have been reported to be altered in cystinotic human proximal tubule cells, but not in *CTNS* ^*−/−*^ iPSCs or mouse cystinosis fibroblasts. These discrepancies have been suggested to be attributed to metabolic differences between cell types [20].

Western blot analysis revealed that *CTNS* KO in myotubes affected the levels of selective proteins: whilst RyR and MyHC were depleted because of *CTNS* loss, the levels of other proteins remained unchanged (housekeeping proteins, dystrophin). RyR and MyHC proteins are essential for muscle development and differentiation: RyR regulates Ca^+2^ signalling, which controls MyHC transcription and sarcomere assembly [39]. Nevertheless, despite a significant reduction in RyR levels, Ca^2+^ signalling remained unaffected, possibly because of compensatory mechanisms maintaining Ca^2+^ homeostasis [40]. Importantly, LV‐mediated *CTNS* ^*WT*^ *‐3HA* cDNA addition in *CTNS* ^*−/−*^ myoblasts reverted cystine levels to WT, rescued the decreased fusion index of *CTNS* ^*−/−*^ Day 4 myotubes and, in line, restored RyR and MyHC protein levels, confirming a *CTNS* deletion‐specific effect.

Consequently, we performed diaPASEF to get in‐depth comprehension of the proteome levels affected by *CTNS* loss, as well as *CTNS* ^*WT*^ *‐3HA* cDNA addback. From the more than 8000 quantified protein groups, none were affected by *CTNS* deletion in myoblast level. Differently, in Day 4 differentiated myotubes, five protein groups were increased, and a network of 18 biologically connected proteins was depleted in *CTNS* ^*−/−*^ myotubes. In line with western blot results, this network included RyR1 (the predominant form of RyR in skeletal muscle) and three MyHC isoforms (MYH3, MYH7 and MYH8, which are crucial for muscle regeneration) [28, 29]. In addition, the RYR1 regulators ASPH and CACNB1 were also found in this network of proteins, as well as several myosin‐interacting proteins critical for sarcomere function, including myosin binding protein‐C (MYBPC2), unc‐45 myosin chaperone B (UNC45B), troponin T3 (TNNT3), leiomodin 2 (LMOD2) and nebulin (NEB).

The addback of the lysosomal catalytic dead ATP13A2 (dATP13A2, LV_Ctrl) protein aggravated the phenotype, further reducing the level of proteins found to be depleted in CTNS‐deficient cells. The latter could be attributed to the residual scaffolding role of the catalytically inactive dATP13A2 [32]. The proteomics findings indicate disruptions in sarcomere organization and muscle contraction upon *CTNS* loss, which suggests an impairment in muscle homeostasis and repair. These pathways are frequently implicated in myopathies, and loss‐of‐function mutations in many of these proteins are associated with musculoskeletal disorders (*Supplementary Table* *S5*).

To further characterize these molecular changes, we performed transcriptomic and metabolomic analyses. Temporal transcriptome analysis of the depleted proteins confirmed the myotube‐specific gene expression of the protein groups and that the affected protein‐coding genes were primarily downregulated in CTNS^−/−^ myotubes. Although in Day 4 differentiated myotubes the only metabolite that was statistically significantly altered was cystine, unsupervised clustering showed a similitude of metabolomes of conditions with a functional CTNS (WT and CTNS^−/−^ + LV_*CTNS* ^*WT*^ *‐3HA*), in line with studies using other cell lines reporting a metabolic imbalance caused by *CTNS* deletion [21]. These data suggest that *CTNS* loss may slow or mildly impair myotube formation through metabolic dysregulation, which could compromise muscle regeneration in people with cystinosis.

The current model only captures early stages in myogenesis and lacks factors such as the extracellular matrix (ECM) interactions, 3D culture conditions and interactions with other cell types. For example, macrophages have been identified with cystine crystals in the perimysium of muscle biopsies from patients. Additionally, recent studies indicate abnormal neurophysiological features in people living with cystinosis, suggesting a neuromuscular involvement, including potential contributions from motor neurons or neuromuscular junction defects [7, 38].

The LHCN‐M2 cell line, which is immortalized human skeletal myoblasts from a healthy male donor, does not recapitulate the genetic context of cystinosis [22]. This line is used in muscle research for its well‐characterized myogenic properties, reproducibility and suitability for controlled genetic manipulation. By introducing CTNS knockout in this background, we aimed to isolate the direct effects of CTNS loss on muscle cell biology, minimizing confounding variables. Future studies using patient‐derived myoblasts or induced pluripotent stem cell (iPSC)‐derived muscle cells from individuals living with cystinosis would provide valuable insights into the broader genetic and epigenetic contributions to disease.

In conclusion, despite its limitations, this first human myoblast‐based model highlights early muscle defects caused by *CTNS* deletion. For the first time, we identified that *CTNS* loss in myotubes leads to a depletion of an 18‐protein network involved in myofibril assembly and implicated in other myopathies. Moreover, we demonstrated that the observed phenotype was reverted upon LV‐mediated *CTNS* ^*WT*^ *‐3HA* cDNA addition, underscoring the potential of gene therapeutic intervention. Further development and refinement of this model, combined with more mature muscle differentiation approaches, will undoubtedly provide a deeper understanding of the underlying molecular mechanisms contributing to cystinosis myopathy and spur the development of rapidly implementable therapeutic approaches.

## Conflicts of Interest

The authors declare no conflicts of interest.

## Supporting information


**Table S1:** Overview of primer sequences.
**Table S2:** Overview of primary and secondary antibodies.
**Table S3:** Window widths PASEF scans proteomics analysis.
**Table S4:** Assessment of the off‐target activity of Cas9‐VLP targeting CTNS. Quantification of indel events in CTNS−/− myoblasts at off‐target sites in RALBP1, CNTNAP2 and PTTG1IP using guide validation and ICE analysis of Synthego.
**Table S5:** Correlation of loss‐of‐function or depletion in different proteins from the identified downregulated cluster with different myopathies.

**Figure S1:** **Validation of CTNS knock‐out in immortalized human myoblasts at cDNA level.** (A) Quantification of indel events in CRISPR‐edited CTNS^−/−^ myoblasts at cDNA level using DECODR analysis. (B) Modification and predicted stop codon (*) in exon 5 resulting in a 58 AA truncated protein (SnapGene).
**Figure S2:** **Analysis of WT and CTNS** ^**−/−**^ **myotube differentiation.** (A) Individual plots of each myogenic differentiation experiment (each n) corresponding to the plot of Figure 2D. Each plot represents 10 ROIs, error bars represent median with 95% CI, numeric values correspond to median. Statistical testing was performed with an unpaired *t* test. (B)Representation of medians of the five replicates of fusion index, normalized to WT. Statistical testing was performed with one sample t and Wilcoxon signed rank test. (C) Covered area by myotube per region of interest (ROI), number of myotubes per ROI and branching points per myotube between WT and CTNS^−/−^ Day 4 myotubes. Each dot represents an individual image field, data are show the median with 95% CI (*n* = 5). **p < 0.01; ns, nonsignificant, *p* > 0.05. Statistical testing was performed with an unpaired *t* test. (D) Binary mask images and branching points of representative images of WT and CTNS^−/−^ Day 4 myotubes.
**Figure S3:** **Analysis substrates of the mTOR pathway in WT and CTNS** ^**−/−**^ **myoblasts.** (A) Representative western blot analysis of (p)S6 and (p)70S6K1 protein expression in WT and CTNS^−/−^ myoblasts under different feeding conditions. 4‐h incubation with EBSS was used as the starvation condition. Samples normalized for total proteins of vinculin. (B) Quantification of (p)S6 and (p)70S6K1 protein expression in WT and CTNS^−/−^ myoblasts (*n* = 3 independent experiments). Samples normalized for total proteins of vinculin. Statistical testing was performed with a one‐way ANOVA, Sidak's multiple comparison test. ***p < 0.001; **p < 0.01; ns, nonsignificant, *p* > 0.05.
**Figure S4:** **The RyR‐mediated Ca** ^**2+**^ **release remains unaltered in CTNS** ^**−/−**^ **myotubes.** (A–B) Representative intracellular Ca^2+^ measurement of 4‐day differentiated WT (A) and CTNS^−/−^ (B) myotubes after the addition of different (colour coded) concentrations of Ach (M) (*n* = 5). Dotted lines represent error bars. (C) Peak response of Ca^2+^ release after the addition of a concentration of Ach (nM) in WT and CTNS^−/−^ 4‐day differentiated myotubes. Data are represented as mean ± SD (*n* = 5 independent experiments).
**Figure S5:** **Covered area by myotube rescue and proteome changes in WT and CTNS** ^**−/−**^ **cells during differentiation.** (A) Covered area by myotubes of WT, CTNS^−/−^ an and CTNS^−/−^ complemented with either LV_CTNS^WT^‐3HA or LV_Ctrl represented by bar plots with each dot representing an individual image field. The data for WT and CTNS^−/−^ are the same datapoints as in Supplementary Figure 2C. Data are presented as median with 95% CI of *n* = 6 (for WT and CTNS^−/−^) and *n* = 4 (for CTNS^−/−^ + LV addbacks) independent myogenic differentiations (plotted in different shapes). Statistical testing was performed with one‐way ANOVA, Sidak's multiple comparison test. LV, lentiviral vector; WT, wild‐type; **p < 0.01; ***p < 0.001. (B) Representative immunofluorescence images of CTNS−/− + LV_CTNSWT‐3HA/LV_Ctrl myotubes after 4 days of myogenic differentiation. Staining by nucleus (DAPI, blue), MyHC (red) and merged. Scale bar: 100 μm. (C) Volcano plot of altered proteins in WT cells between Day 0 (Mb) and Day 4 (Mt). (D) Volcano plot of altered proteins in CTNS^−/−^ cells between Day 0 (Mb) and Day 4 (Mt). The thresholds correspond to a *q* value < 0.05 with the multiple *t* test using Two‐stage step‐up method of Benjamin, Krienger and Yekutieli false discovery rate (FDR) approach. (E) Heatmap of myoblast (MYF5) or myotube (RYR1, MYH1, MYH3, MYH8, MEF2C, MYOG, TNNT1, MYOD1, DES, MYH2, DMD) specific markers specifically increased or decreased upon myotube differentiation [33–41]. In white, nondetected data; *n* = 3.
**Figure S6:** **Volcano plots of proteomic analysis in myoblasts and myotubes between WT and CTNS** ^**−/−**^ **conditions.** (A) Volcano plot of differential proteins between WT and CNTS^−/−^ cells on Day 0 (myoblasts, Mb) and (B) on Day 4 (myotubes, Mt). The thresholds correspond to a *q* value < 0.05 with the multiple *t* test using Two‐stage step‐up method of Benjamin, Krienger and Yekutieli false discovery rate (FDR) approach, under which all samples are categorized as nonsignificant.
**Figure S7:** **Proteomics analysis corroborates previously acquired western blot data.** (A) Relative quantitative levels on Day 4 between all four conditions of all the RyR and MyHC isoforms identified through DiaPASEF technology. Those isoforms that are part of the cluster affected by CTNS deletion are on top (RyR1, MYH3, MYH7, MYH8) whereas the nonmodified isoforms are on the bottom (RyR3, MYH1,2,9,10,13,14); *n* = 3. Percentages underneath each isoform correspond to the relative abundance of the protein groups in WT cells (counts) in comparison to the total counts of that protein group isoform. * Unadjusted *p* < 0.05. ** unadjusted *p* < 0.01. *** unadjusted *p* < 0.001. ns, nonsignificant, unadjusted *p* > 0.01. (B) Heatmap of protein abundance of all the different MyHC isoforms identified between WT and CTNS^−/−^ conditions in myoblasts and myotubes, *n* = 3. In white, nondetected data (C) Transcripts heatmap of all the different MyHC isoforms identified between WT and CTNS^−/−^ conditions in myoblasts and myotubes, *n* = 4. In white, nondetected data.
**Figure S8:** **Abnormalities associated with alterations in the protein groups from the depleted cluster.** Human phenotype (Monarch) enrichment, obtained from https://string‐db.org/.
**Figure S9:** **Heatmap of modified proteins.** Comparison of protein group levels (log2 fold change from average) between WT and CTNS^−/−^ cells and between Day 0 and Day 4. Nondetected data are displayed in white; *n* = 3.
**Figure S10:** **Transcriptome and metabolome analysis.** (A) Normalized counts of MYF5 mRNA expression during the 4 days of myoblast differentiation for WT and CTNS^−/−^ cells. Data are presented as the mean ± SD (*n* = 4). (B) Volcano plot of all altered gene expression between WT and CTNS^−/−^ myotubes (C) and for selection of 23 differentially represented protein groups at Day 0 and (left) and Day 4 (right). The threshold corresponds to *q* value < 0.05. Reported *p* values underwent adjustment for multiple testing using the Benjamini–Hochberg procedure to control the false discovery rate (FDR), thresholds correspond to a *q* value < 0.05. (D) Normalized counts of myogenesis mRNA expression levels over time on WT and CTNS^−/−^ myoblasts during the 4 days of myotube differentiation for the protein groups identified in the depleted protein network not shown in Figure 8.B and (E) for the identified increased protein groups. Data are presented as the mean ± SD, *n* = 4. (F) Complete unsupervised hierarchical clustering of metabolic levels of Day 4 myotubes (*n* = 3 independent metabolomic extractions).

## Data Availability

The datasets used and/or analyzed during the current study are available from the corresponding author on reasonable request.
