# Supplementary material for: An Isogenic Human Myoblast Cell Model for Cystinosis Myopathy Reveals Alteration of Key Myogenic Regulatory Proteins
Source: J Cachexia Sarcopenia Muscle. 2025 Nov 10;16(6):e70116. doi: 10.1002/jcsm.70116 (PMC12598300; doi:10.1002/jcsm.70116)
Supplement: Supplementary file 1 — Table S1: Overview of primer sequences. Table S2: Overview of primary and secondary antibodies. Table S3: Window widths PASEF scans proteomics analysis. Table S4: Assessment of the off‐target activity of Cas9‐VLP targeting CTNS. Quantification of indel events in CTNS−/− myoblasts at off‐target sites in RALBP1, CNTNAP2 and PTTG1IP using guide validation and ICE analysis of Synthego. Table S5: Correlation of loss‐of‐function or depletion in different proteins from the identified downregulated cluster with different myopathies. [file JCSM-16-e70116-s001.docx]

**An Isogenic Human Myoblast Cell Model for Cystinosis Myopathy Reveals Alteration of Key Myogenic Regulatory Proteins**

Louise Medaer^1^*, Roger Mora^1^*, Zhuoheng Zhou^2^, Nefele Giarratana^3^, Laura Yedigaryan^3^, Rita La Rovere^4^, Elena Levtchenko^5^, Vincent Mouly^6^, Els Verhoeyen^7,8^, Sebastiaan Eeltink^2^, Achim Treumann^9^, Tim Vervliet^4^, Maurilio Sampaolesi^3,10^, Rik Gijsbers^1,11^

*These authors contributed equally to this work.

**Institutional information**

^1^Advanced Disease Modelling, Targeted Drug Discovery and Gene Therapy (ADVANTAGE), Department of Pharmacological and Pharmaceutical Sciences, Faculty of Medicine, KU Leuven, 3000 Leuven, Belgium.

^2^Vrije Universiteit Brussel (VUB), Department of Chemical Engineering, Brussels, Belgium.

^3^Stem Cell and Developmental Biology Unit, Department of Development and Regeneration, Faculty of Medicine, KU Leuven, 3000 Leuven, Belgium.

^4^Laboratory for Molecular and Cellular Signaling, Department of Cellular and Molecular Medicine, Faculty of Medicine, KU Leuven, 3000 Leuven, Belgium.

^5^Emma Children Hospital Amsterdam UMC, 1105 AZ Amsterdam, Netherlands.

^6^Myoline Platform, Sorbonne Université, Inserm, Institut de Myologie, Centre de Recherche en Myologie, F-75013 Paris, France.

^7^Université Côte d’Azur, Institute for health and medical research (INSERM), Mediterranean Centre for Molecular Medicine (C3M), 06204 Nice, France.

^8^International center for infectiology research (CIRI), Université de Lyon; INSERM U1111; Ecole normale supérieure (ENS) de Lyon; University Lyon1; Centre national de la recherche scientifique (CNRS), UMR5308, 69007 Lyon, France.

^9^Cystinosis Ireland, Dublin 2, Ireland.

^10^Histology and Medical Embryology Unit, Department of Anatomy, Histology, Forensic Medicine and Orthopedics, Sapienza University of Rome, 00185 Rome, Italy.

^11^Leuven Viral Vector Core, Group Biomedical Sciences, Faculty of Medicine, KU Leuven, 3000 Leuven, Belgium.

**Supplementary materials and methods**

**sgRNA design**

guideRNAs (gRNA) have been designed targeting exon 4 of CTNS using an online software tool (Deskgen). 20 bp long oligos were ordered from Integrated DNA Technologies (Leuven, Belgium) annealed and cloned into the gRNA-expressing plasmid using the restriction site BsmBI: 5’-TTACGACAGGAGGAACAGTG-3’ [1]. All cloning steps were corroborated by Sanger sequencing.

**Production of nanoblades**

HEK293T cells were seeded in 10-cm culture dishes 24 h before transfection (70-80% confluency). Nanoblades (Nbs) were produced essentially as previously described [2]: plasmids coding for MLVGagPol, GagCas9, BaEVRless, VSV-G, and gRNA expression plasmid were co-transfected with transfection reagent at a 2.6/1 ratio of linear polyethylenimine (PEI, MW 25 000; Polysciences Inc, Hirschberg an der Bergstrasse, Germany). The medium was replaced 24 h after transfection by 6 mL of OptiMEM (Life Technologies, Thermo Scientific) supplemented with 50 µg/ml gentamycin. The next two days, Nb-containing medium was collected and filtered through a 0.45 µm Millex syringe pore-sized filter (Merck) and concentrated using Amicon ultra 15 ml vivaspins (Merck) at 3000 rpm. The supernatant was carefully removed by aspiration to obtain 1 mL of x-fold concentrated Nbs.

**Detection of on-target editing efficiency**

gDNA was extracted using GenElute™ Mammalian Genomic DNA Miniprep Kit (Sigma Aldrich). mRNA was extracted using the AurumTM Total RNA Mini Kit (Bio-Rad, Temse, Belgium) following manufacturers’ instructions. cDNA was synthesized from the extracted mRNA samples using the High-Capacity cDNA Reverse Transcription Kit (Applied Biosystems, Merelbeke, Belgium). PCR amplification was performed using iProofTM high-fidelity DNA polymerase (Bio-Rad) and primers amplifying 280 bp (gDNA) or 467 bp (cDNA) region surrounding the cut site of the sgRNA (Supplementary Table 1). PCR fragments were verified on agarose gel and purified with GeneJet PCR purification kit (Thermo Scientific). Afterwards, the PCR fragments were sent for Sanger sequencing (LGC Genomics GmbH, Berlin, Germany). The sequencing results were analysed using ICE (https://ice.synthego.com/#/) or DECODR (https://decodr.org/).

**Detection of off-target editing**

Off-target sites for the sgRNA targeting CTNS were determined by the Guide Verification Tool of Synthego (https://design.synthego.com/#/validate). Three off-target sites were selected for further analysis. PCR amplification and fragments were purified as described above (*Supplementary Table 1*). The PCR fragments were sent for Sanger Sequencing and analysed using ICE (https://ice.synthego.com/#/) or DECODR (<https://decodr.org/>).

**Determination of growth curve**

Myoblasts (2,000 cells/cm^2^) were seeded in a 6-well plate and counted every day for 5 days using a Z1 Particle Counter (Beckman Coulter). To determine the growth rate and the cell doubling time, a nonlinear regression was performed using GraphPad 8.0. The cell growth rate in hours (G) was calculated using the following equation: y(t) = a * e^Δt^, where y(t) is the number of cells/ml at time point t and ‘a’ is the number of cells/ml at t = 0 h.

**Immunocytochemistry staining**

Myoblasts (15,000 cells/well) were seeded in Poly-D-lysin coated (Thermo Scientific), non-detachable chambers (Ibidi, Beloeil, Belgium) and, after 24 hours, fixed with 4% paraformaldehyde (PFA; Sigma Aldrich) for 20 min at RT. To permeabilize, the myoblasts were incubated with 0.2% triton X-100 (Acros Organics, Geel, Belgium) for 30 min. The cells were washed with 1x PBS and subsequently blocked for 30 min with 1% bovine serum albumin (BSA; Sigma Aldrich) or 10% donkey serum (Biowest, Leuven, VWR Belgium). After washing with PBS, the cells were incubated ON at 4 °C with primary antibodies diluted in PBS with 0.1% BSA (*Supplementary Table 2).* The next day, the cells were washed with PBS and incubated for 30 min with secondary antibodies (Thermo Scientific) diluted in PBS with 0.1% BSA and DAPI (1/2000, Sigma Aldrich) diluted in Mowiol mounting medium (Sigma Aldrich). The differentiation experiments were visualized with Nikon Eclipse Ti-S microscope (Nikon, Tokyo, Japan) and Zeiss LSM 780 (Cell Imaging Cor, KU Leuven).

Quantification of different parameters of myotube differentiation (fusion index, myotube coverage, branching, number of myotubes) was conducted using the Myotube Analyzer [3]. Fusion index was quantified by the percentage of nucleus in MyHC-positive areas using the following parameters: “Adjust levels” 0.4–0.8 and the “Adjust threshold” 0.07-0.10.

The samples for localization of CTNS were visualized with a laser scanning confocal microscope (Zeiss LSM 780; Cell Imaging Core (KU Leuven)) in combination with a Plan-Apochromat 63x/1.4 Oil DIC M27 objective (Zeiss, Brussel, Belgium) and the following lasers: 488 nm, 561 nm, 633 nm, and 405 nm.

**Ca^2+^ measurements**

Myoblasts (16,000 cells/well) were seeded in a collagen I-coated 96-well plate (Greiner; 655090) and differentiated over a 4-day period. On day 4 of differentiation, the myotubes were incubated with 2 µM Cal520 solubilized in differentiation medium for 30 min. Afterwards the cells were washed twice with differentiation medium after which de-esterification was allowed for 45 min at 37 °C 5% CO_2_. Ca^2+^ imaging was performed in Krebs-ringer solution (135 mM NaCl, 6.2 mM KCl, 1.2 mM MgCl_2_, 12 mM HEPES, pH 7.3, 11.5 mM glucose and 2 mM CaCl_2_). For acetylcholine (Ach) stimulus, different concentrations 10 nM - 50 µM (Sigma Aldrich) were prepared in Krebs-Ringer solution. Imaging was performed using a functional drug discovery system (FDSS) from Hamamatsu with excitation 470 nm and emission 520 nm. Analysis was performed with excel.

**Lysosomal pH measurements**

Myoblasts (125,000 cells/chamber) were seeded in collagen I-coated 35 mm 4-chamber glass bottom dishes (Cellvis; D35C4-20-0-N) and differentiated over a 4-day period. At day 3 of differentiation, the cells were incubated with rhodamine-dextran (0.125 mg/ml, Sigma Aldrich) and fluorescein isothiocyanate (FITC)-dextran (0.25 mg/ml, Sigma Aldrich). 24 hours after incubation, cells were washed and replaced with differentiation medium for 3 h at 37°C 5% CO_2_. For the Bafilomycin A1 (BafA1) condition, 100 nM BafA1 (Santa Cruz) was added to the differentiation medium. Before imaging, medium was replaced by Krebs-Ringer solution with Hoechst (concentration stock 1 µM; Thermo Scientific). Imaging was performed using a Nikon eclipse Ti2 inverted fluorescence microscope (Nikon) with a 40x oil objective (Nikon). For the acquisition of fluorescent signal for FITC, Rhodamine, and Hoechst, the following excitation/emission wavelengths were used respectively: 470/520 nm, 455/570 nm, and 365/500 nm. For analysis Image J FIJI software was used to quantify FITC and rhodamine intensities and count the nuclei following background subtraction and thresholding.

**Production of LV vectors and generation of stable cell lines expressing CTNS^WT^**

Lentiviral vectors (LVs) were produced as previously reported [4]. Functional validation of the LV_*CTNS^WT^-3HA* constructs was reported in Veys et al. and Medaer et al. [2, 4]. To ensure a single integrated viral vector copy per cell, viral vector transduction was conducted employing a limiting dilution series. Cells were seeded in a 48-well plate to reach the ideal confluency at 20,000 cells, grown overnight and transduced with the respective LV vector preparations. 48 hrs later, the medium was replaced with puromycin (1 µg/ml, ant-pr-1; Invivogen, Toulouse, France) containing medium to select transduced cells. To ensure a single integrated viral vector copy, we selected the highest dilution that still resulted in surviving cells upon puromycin selection (<20% transduced cells, MOI <0.5) [5].

### **Proteome analysis**

### Sample preparation and quality controls

Myoblasts (10,000 (Day 0) – 50,000 (Day 4) cells/cm^2^) were differentiated over a 4-day period in collagen I-coated 6-well plates. Protein extraction using 1% Sodium dodecyl sulfate (SDS, Sigma Aldrich) was performed on day 0 and day 4 of differentiation followed by heating of the extract for 5 min at 98°C. After DNA shearing with a 0.3 mL insulin needle, the samples were again heated for 5 min at 98 °C. The total protein concentration was determined using the Pierce^TM^ BCA Protein Assay Kit (Thermo Scientific).

A QC pool consisting of 10 µL of each sample was established. This was used to set up the optimized dia-PASEF method and to monitor system performance (Peptide and protein identifications, retention times and peak widths of all QC samples) at the beginning of the sample sequence and after every 8 samples. 100 ng of a HeLa protein digest (Thermo Scientific) and 200 ng of QC-pool were injected. t. The order of the samples was randomized, and a blank run was inserted between every sample injection.

Tryptic digestion of the protein samples and the QC pool was performed using S-Trap with a protein-to-enzyme ratio of 10:1 according to the manufacturer’s instructions [6]. The spin-dried samples were reconstituted with 0.1% formic acid/water solution to a final protein concentration of 100 ng/µL.

### LC-MS instrumentation and separation/MS acquisition methods

Liquid chromatography (LC) separations were performed on a Vanquish Neo™ UHPLC system, with a 110 cm μPAC™ Neo HPLC Column (Thermo Scientific). The column was kept at 60 °C with an external column-heater (Pheonix ST, MS Wil B.V., Aarle-Rixtel, The Netherlands) and mounted in front of the MS ion source with a home-made frame. A binary mobile phase was used for gradient separation with mobile phase A (MPA) comprising of water and 0.1% (v/v) FA and mobile phase B (MPB) containing 20/80 (v/v) water/acetonitrile and 0.1% (v/v) FA. Injection volume was set at 2 µL for each sample (ca. 200 ng protein loaded on column).

A three-stage segmented linear gradient was applied with flow rate ramping. Briefly, MPB increases from 1% to 10% in the first 6 min with a flow rate of 750 µL/min to rapidly transport the sample through the system void volume. From 6 to 33 min, MPB increases from 10% to 22.5% applying a flow rate of 250 µL/min. MPB further increases to 45% from 33 min to 48 min with flow rate unchanged, followed by washing and re-equilibrium steps for 18 min. In total, the gradient program yields a 90 min inject-to-inject cycle time.

Tandem mass spectrometry (MS/MS) data were acquired on a timsTOF Pro 2 mass spectrometer (MS) with a CaptiveSpray ion source (Bruker Daltonics, Kontich, Belgium). The MS was weekly calibrated with positive TOF calibration standard (Agilent, Zaventem, Belgium) for m/z ranging from 100 to 1700 Th and ion mobility (IM) ranging from 0.85 to 1.3 V·s/cm2. A reduced calibrant mixture containing ions of m/z = 622, 922 and 1222 was deposited on the inline filter of the ion source for daily calibration. The LC column outlet was connected to a 10 µm i.d. silica capillary emitter with a 1500 V capillary voltage applied for ionization, drying gas (99.99999% N_2_) heated up to 180 °C was supplied internally flowing at 3 L/min to facilitate ion desolvation. timsControl (version 4.0) and Compass Hystar (version 6.0) were used for MS/MS method editing and acquisition, respectively. All MS/MS data were acquired in a data-independent manner with parallel accumulation–serial fragmentation activated (dia-PASEF). For dia-PASEF scan window optimization, 200 ng of pooled sample was injected on LC and a system default data-dependent (dda)-PASEF method was used for scouting the peptide-like ion distribution on the IM-m/z plane. The open-source python package py_diAID was used for dia-PASEF isolation window width optimization, aiming to achieve an equal number of precursors fragmented per isolation window [7].

In the dda-PASEF scouting method, the IM and m/z range are set the same as for calibration (i.e., 100 to 1700 Th and 0.85 to 1.3 V·s/cm2, respectively). 100% duty cycle was selected for IM separation with 100 ms for both voltage ramping and ion accumulation, yielding a ramping frequency of 9.4 Hz. For each MS/MS cycle, 4 PASEF ramps were executed leading to a total cycle time of 0.53 s. Precursor target intensity was set at 20,000 a.u. with minimum threshold of 2500 a.u. Active exclusion was enabled with a duration of 0.4 min with m/z tolerance of 0.015 Th. Collision induced dissociation (CID) was deployed for fragmentation, the collision energy increased from 27.8 eV to 49.3 eV and linearly synchronized with precursor’s IM value. A spectral library was built based on the dda-PASEF scouting result with PASER Data Viewer (Bruker, ver2023b) and imported to py_diAID, the optimized isolation window scheme generated by py_diAID was imported back to timsControl window editor for dia-PASEF method development. In total, 24 PASEF scans were defined covering m/z range from 433 to 1198 Th and IM range from 0.85 to 1.3 V·s/cm^2^ with varying window widths (*Supplementary Table 3*). The total cycle time of the method was estimated to be 1.38 s. The IM ramping and CID settings remained the same as in the dda-PASEF method.

### Data processing and statistical tests

The DIA MS/MS spectra were searched with DIA-NN (version 1.8.1) in library-free mode [8, 9] against UniProt sequences of homo sapiens (updated on 10-23-2023, organism ID 9606 with 20386 reviewed entries) and a list of common contaminants for cells grown in culture. For in-silico library generation, the search parameters were as follows: Trypsin/P was selected as protease with maximum 1 missed cleavage allowed; protein N-terminal methionine excision was allowed; S-thiomethylation cysteines was set as fixed modification and no variable modification allowed; peptide length was set from 7 to 30 with precursor charge from 1+ to 4+; m/z range for the precursor and fragment were set from 434 to 1200 Th and from 100 to 1700 Th, respectively. The search results were filtered with q value < 0.01 for protein groups at the library level. For the main search, precursor false discovery rate (FDR) was set at 1%; mass accuracy at MS1 and MS2 were both set to 0; isotopologues and match-between-run were activated; protein inference was set at gene level and heuristic protein inference was enabled; quantification strategy was set to Robust LC (high precision) and the neural network classifier was set to double-pass mode; cross-run normalization was turned off.

Subsequent data analysis was carried out with the Perseus 2.0.11 software package. After removal of contaminant hits and assignment to groups, samples were filtered for non-zero values in all conditions and intensity values were log2-transformed.

False Discovery Rate (FRD) adjusted p-value (q-value) volcano plots between WT and *CTNS^-/-^* conditions were drawn using GraphPad 8.0.2 software. Multiple hypothesis testing was executed using the Benjamin-Hochberg procedure for FDR (q-value < 0.05).

Differential protein group analysis between conditions was executed using three consecutive student’s t-test (unadjusted, p-value < 0.01) between pairs of conditions (WT vs *CTNS^-/^*^-^; *CTNS^-/-^* vs LV_CTNS^WT^-3HA; LV_CTNS^WT^-3HA vs LV_Ctrl) in order to discern protein groups affected by CTNS KO and restored upon *CTNS* addback, although not upon control addback. The analysis was performed separately for the increased and depleted protein group clusters. Only those protein groups showing differential abundance (p-value < 0.01) in all 3 above mentioned comparisons were selected.

Raw data deposition

All raw MS data are available via the Proteomexchange data repository ([www.proteomexchange.org](http://www.proteomexchange.org)) with the identifier PXD052308.

**Bulk RNA sequencing and analysis**

Myoblasts were seeded and differentiated over a 4-day period (day 0 – day 4). Samples were acquired in 5 independent experiments. Each day RNA was extracted using AurumTM Total RNA Mini kit (Bio-Rad). The concentration and quality were assessed with Nanodrop and BioAnalyzer, respectively. Samples were prepared and analysed by the Genomics Core (KU Leuven – UZ Leuven, Leuven, Belgium). The QuantSeq 3′ mRNA-Seq Library Prep kit from Illumina (Lexogen, Vienna, Austria) was used according to the manufacturer’s protocol with 500 ng input. Afterwards, the concentration (ng/µL) and fragment length (bp) of the libraries were measured with Qubit and Fragment analyser, respectively to pool the libraries in equimolar amounts. The pool was quantified with RT-qPCR and a final pool was made for single-read sequencing on the HiSeq4000 (Illumina Inc.).

Quality control of raw reads was performed with FastQC v0.11.7 [10]. Adapters were filtered using Trimmomatic v0.39 [11]. Splice-aware alignment was carried out using Hisat2 against the reference genome using the default parameters [12]. Quantification of reads per gene was conducted using FeatureCounts from the Subread package [13]. Count-based differential expression analysis was performed using the R-based Bioconductor package DESeq2 [14]. P values were corrected for multiple testing using the Benjamin-Hochberg method. After analyzing n = 5 biological repeats as shown in PCA plot of *Figure 8.B*, one experiment was discarded after a quality control filter due to insufficient RNA concentration and abnormal profile of differentiation markers in WT condition. Multiple hypothesis testing was executed using the Benjamin-Hochberg procedure for FDR (q-value < 0.05).

**Metabolome analysis and cystine measurements**

The myoblasts were seeded 50,000 cells/cm^2^ in a collagen I-coated 6-well plate and allowed to differentiate at 37 °C for 4 days. Samples were prepared by removing the medium and washing the cells with a 0.9% NaCl solution. The washing solution was removed, and the extraction buffer was added. The extraction buffer with cystine internal standard was prepared as follows: a 20 mM ^15^N_2_-Cystine stock standard was prepared by dissolving 4.8 mg ^15^N_2_-cystine (Cambridge Isotope Laboratories NLM-3818; Apeldoorn, The Netherlands) in 1 mL of a 30/70 (v/v) mixture of 2M HCl (fuming 37%, 1.00317.1000, Merck) in milliQ water and methanol (85800.320; VWR, Leuven, Belgium), respectively. This solution was then diluted to insert a final concentration of 20 µM in a solution of 80/20 (v/v) methanol/milliQ water with 0.1 v% formic acid (85048.001; VWR, Leuven, Belgium). Using a cell scraper, the extract was transferred into an Eppendorf tube. Proteins were pelleted by centrifugation for 15 min at 20,000× *g* at 4 °C and their concentration was determined by a Pierce^TM^ BCA protein assay (Thermo Scientific). The supernatant was transferred to a new Eppendorf tube and subjected to metabolomics analysis using a Vanquish LC System (Thermo Scientific) coupled via heated electrospray ionization to a Q Exactive Orbitrap Focus mass spectrometer (Thermo Scientific). A 10 μL sample was separated (flow rate 0.25 mL/min, 25°C) on a Poroshell 120 HILIC-Z PEEK Column (Agilent InfinityLab, Zaventem, Belgium) using a linear gradient starting with 90% solvent A (acetonitrile with 5 µM medronic acid) and 10% solvent B (10 mM NH_4_-formate in milli-Q water, pH 3.8) and increasing to 60% B in 10 min. The gradient was kept on 60% B for 3 min followed by a decrease to 10% B. The chromatography was stopped at 25 min. The flow was kept constant at and the column was kept at 25 °C throughout the analysis. The mass spectrometer was operated in positive ion mode (spray voltage of 3 kV, capillary temperature of 320 °C, sheath gas at 45, auxiliary gas at 10, heated to 260 °C) in full scan mode (m/z 70.000–1050.000) at a resolution of 70,000 and an AGC target of 3.0E+06. Data collection was performed using the Xcalibur software 4.2.47 (Thermo Scientific). Relative quantification of metabolites (metabolites from glycolysis, the Krebs cycle, amino acids, nucleotides, energy charge and redox molecules) was executed by integrating MS peak areas (El-Maven–Polly–Elucidata), and absolute quantification of cystine was executed by ratio comparison to the MS signal of ^15^N_2_-cystine spiked in the extraction buffer.

The cystine concentration was normalized according to the total protein content (nmol cystine/mg protein). Subsequent data analysis was carried out with the Perseus 2.0.11 software package. Samples were filtered for non-zero values in all conditions and intensity values were log2-transformed. Non-supervised hierarchical clustering was performed using Pearson correlation distance with complete linkage. Q-value (FDR adjusted p-value) volcano plots between WT and *CTNS^-/-^* conditions were drawn using GraphPad 8.0.2 software. Multiple hypothesis testing was executed using the Benjamin-Hochberg procedure for FDR (q-value < 0.05).

**Author contributions**

Conceptualization: L.M., R.G., M.S., S.E., A.T., T.V. ; Methodology: L.M., T.V., Z.Z., N.G., L.Y. R.L.R., E.V., V.M. ; Software, R.M.; L.M.; T.V., Z.Z., A.T. ; Validation, L.M., R.G., T.V., Z.Z., N.G., L.Y., R.L.R., R.M., A.T., S.E., M.S. ; Formal analysis and investigation: L.M., R.M., R.G., V.T., N.G., R.L.R., Z.Z., A.T., M.S. ; Resources: L.M., R.G., M.S., E.V., V.M., S.E.; Data curation: L.M., R.M., R.G., Z.Z., S.E., A.T. ; Writing - draft preparation: R.M., L.M., R.G., A.T., M.S. ; Writing - review and editing, R.M., L.M., R.G., M.S., T.V., E.L., L.Y, S.E., A.T. ; Supervision: S.E., M.S., R.G. ; Project administration: L.M., R.M, R.G. ; Funding acquisition: L.M., R.G., E.L., M.S..

**Supplementary Tables**

***Supplementary Table 1. Overview of primer sequences.***

| TARGET | cDNA/gDNA | FORWARD 5’ 🡪 3’ | REVERSE 5’ 🡪 3’ |
| --- | --- | --- | --- |
| *WPRE* | gDNA | CAATTCCGTGGTGTTGTCGG | GAAGGTCCGCTGGATTGAGG |
| Hs *CTNS* | cDNA - qPCR | CCACAGGCCTACATGAACTT | TCCACTGGTCGTTGTTGTAG |
|  | cDNA - PCR | TGCTTTGGAGACGCTGAGAG | AGGAGGCACCACAACTTCAT |
|  | gDNA | GTTTCGGCCCAGTTTTC | TACCTGTAAGACAAGCCCTA |
| Hs *RALBP1* | gDNA | TGGCATTTTGCTGCTGGTAT | ATTTGAGTTCCTGTGGCTTCTG |
| Hs *CNTNAP2* | gDNA | TGGCAAAGATGAGGATAGGGA | GTGCCAGAATCATCCCTTCAA |
| Hs *PTTG1IP* | gDNA | TCCTGTGTCCTGAGGTTGTT | CCCTCGAAGTAACCTTCCCA |
| Hs *ACTB* | gDNA | TCACCCACACTGTGCCCATCTACGA | CAGCGGAACCGCTCATTGCCAATGG |
| Hs *ACTG* | cDNA | CACTGAGCGAGGCTACAGCTT | CACTGAGCGAGGCTACAGCTT |
| Hs *PAX3* | cDNA | GACAACGCCTGACGTGGA | GCTGATGGAACTCACTGACG |
| Hs *MYOG* | cDNA | AAATGGCACCCAGCAGTTG | TAGAAGCGGGGTTCCTGGTA |
| Hs *MYOD1* | cDNA | AGAAGCTAGGGGTGAGGAAG | ATAGCAAAGTGCTGGCAGTC |
| Hs *IGF1* | cDNA | CATGTCCTCCTCGCATCTCT | TACCCTGTGGGCTTGTTGAA |
| Hs *FBXO32* | cDNA | TGAGCGACCTCAGCAGTTAC | CTCTTCTTGGCTGCAACATCA |
| Hs *TRIM63* | cDNA | CCACCTTCCTCTTGACTGCC | CTCTCAGGGCGTCTGCTATG |
| Hs *MSTN* | cDNA | TACCATGCCTACAGAGTCTGATT | TGTTGTAGGAGTCTCGACGG |

***Supplementary Table 2. Overview of primary and secondary antibodies.***

| ANTIBODY | PROVIDER | SPECIES | DILUTION | APPLICATION |
| --- | --- | --- | --- | --- |
| Primary antibodies |  |  |  |  |
| HA.11 | BioLegend 901515 | Mouse | 1/1000 | ICC |
| LA1 | Cell Signaling 9091 | Rabbit | 1/200 | ICC |
| HA.11 | BioLegend 901515 | Mouse | 1/10 000 | WB |
| HA (2-2.2.14) | Invitrogen 26183 | Mouse | 1/2000 | WB |
| vinculin | Sigma V9131 | Mouse | 1/100 000 | WB |
| MF20 (MyHC) | In-house, hybridoma | Mouse | 1/8 | ICC |
| P62 | Abcam Ab155686 | Rabbit | 1/1000 | WB |
| LC3 | Novus Biologicals, #NB600-1384SS | Rabbit | 1/1000 | WB |
| GAPDH | Abcam AB9485 | Rabbit | 1/2000 | WB |
| Dystrophin | Leica Novocastra  NCL-DYS1+2+3 | Mouse | 1/50 | WB |
| RyR | Hybridomabank 34C | Mouse | 1/1000 | WB |
|  | Thermo Fisher MA3-925 | Mouse | 1/1000 | WB |
| MF 20 (MyHC) | In-house, hybridoma | Mouse | 1/3 | WB |
| Secondary antibodies | |  |  |  |
| Anti-mouse Alexa 594 | Invitrogen AB2556543 | donkey | 1/500 | ICC |
| Anti-mouse Alexa 488 | Invitrogen A11001 | Goat | 1/500 | ICC |
| Anti rabbit Alexa 555 | Invitrogen A21429 | Goat | 1/500 | ICC |
| Phalloidin Alexa 633 | ThermoFisher Scientific 21840 | NA | 1/1000 | ICC |
| anti-mouse polyclonal immunoglobulins/HRP | Agilent Dako P0260/P0447 | Rabbit/goat | 1/10 000 | WB |
| Anti-rabbit polyclonal immunoglobulins/HRP | Agilent Dako  P0448 | Goat | 1/10 000 |  |
| Others |  |  |  |  |
| DAPI | Life Technologies D1306 | NA | 1/1000 | ICC |
| Hoechst | Thermo Scientific  33342 | NA | 1/10 000 | ICC |

***Supplementary Table 3. Window widths PASEF Scans Proteomics analysis.***

| **#MS Type** | **Cycle Id** | **Start IM [1/K0]** | **End IM [1/K0]** | **Start Mass [m/z]** | **End Mass [m/z]** | **CE [eV]** |
| --- | --- | --- | --- | --- | --- | --- |
| MS1 | 0 | - | - | - | - | - |
| PASEF | 1 | 0.95 | 1.30 | 699.34 | 716.37 | - |
| PASEF | 1 | 0.85 | 0.95 | 433.76 | 520.77 | - |
| PASEF | 2 | 0.97 | 1.30 | 716.37 | 735.36 | - |
| PASEF | 2 | 0.85 | 0.97 | 520.77 | 541.78 | - |
| PASEF | 3 | 0.98 | 1.30 | 735.36 | 754.71 | - |
| PASEF | 3 | 0.85 | 0.98 | 541.78 | 558.80 | - |
| PASEF | 4 | 0.99 | 1.30 | 754.71 | 774.91 | - |
| PASEF | 4 | 0.85 | 0.99 | 558.80 | 574.78 | - |
| PASEF | 5 | 1.00 | 1.30 | 774.91 | 796.89 | - |
| PASEF | 5 | 0.85 | 1.00 | 574.78 | 590.29 | - |
| PASEF | 6 | 1.01 | 1.30 | 796.89 | 821.91 | - |
| PASEF | 6 | 0.85 | 1.01 | 590.29 | 605.32 | - |
| PASEF | 7 | 1.02 | 1.30 | 821.91 | 848.76 | - |
| PASEF | 7 | 0.85 | 1.02 | 605.32 | 620.33 | - |
| PASEF | 8 | 1.03 | 1.30 | 848.76 | 878.94 | - |
| PASEF | 8 | 0.85 | 1.03 | 620.33 | 635.55 | - |
| PASEF | 9 | 1.04 | 1.30 | 878.94 | 917.45 | - |
| PASEF | 9 | 0.85 | 1.04 | 635.55 | 650.85 | - |
| PASEF | 10 | 1.05 | 1.30 | 917.45 | 966.98 | - |
| PASEF | 10 | 0.85 | 1.05 | 650.85 | 666.34 | - |
| PASEF | 11 | 1.07 | 1.30 | 966.98 | 1033.99 | - |
| PASEF | 11 | 0.85 | 1.07 | 666.34 | 682.82 | - |
| PASEF | 12 | 1.11 | 1.30 | 1033.99 | 1198.59 | - |
| PASEF | 12 | 0.85 | 1.11 | 682.82 | 699.34 | - |

*PASEF, parallel accumulation serial fragmentation; IM, ion mobility ; CE, collision energy*

***Supplementary Table 4. Assessment of the off-target activity of Cas9-VLP targeting CTNS.*** *Quantification of indel events in CTNS^-/-^ myoblasts at off-target sites in RALBP1, CNTNAP2, and PTTG1IP using guide validation and ICE analysis of Synthego*

| **OFF TARGET SITE** | **CHROMOSOME** | **SEQUENCE** | **MISMATCHES** | **INDEL %** |
| --- | --- | --- | --- | --- |
| *RALBP1* | chr18 | TTA**T**G**C**CAGGAGGAACAGTG | 2 | 0 |
| *PTTG1IP* | chr21 | **A**TACG**G**CAGGAGGAAC**G**GTG | 3 | 0 |
| *CNTNAP2* | chr7 | TTAC**A**ACA**T**GAGGA**G**CAGTG | 3 | 0 |

*.*

***Supplementary Table 5. Correlation of loss-of-function or depletion in different proteins from the identified downregulated cluster with different myopathies.***

| **Protein** | **Model** | **Phenotype** |
| --- | --- | --- |
| RyR1 | - Muscle samples of patients - C2C12 myoblasts - Mouse models | - Depletion leads to RyR1-related myopathies [15] - RyR1 depletion leads to molecular alterations observed in myopathies [15] - 50% protein reduction is sufficient to induce muscle disease [16] |
| TNNT3 | Human patient | Deficiency is associated with nemaline myopathy [17] |
| SYNPO2 | Immortalized mice myoblasts | Deficiency leads to decreased myofibrillar stability and deregulated autophagy under mechanical stress [18] |
| NEB | Nebulin-deficient mice | Early death, muscle weakness, shorter skeletal muscle thin filament length, reduction in force production, sarcomere defects [19] |
| LMDO2 | - Human patient - *Lmod2*-null mice | - Cardiomyopathy caused by a biallelic mutation in *LMOD2 [20]* - Early onset of familial dilated cardiomyopathy *[20]* |
| LDB3 | - Human patients - Human patients | - Heterozygous missense variants are associated with cardiomyopathy and myofibrillar myopathy [21, 22] - Biallelic loss leads to a lethal pediatric dilated cardiomyopathy [23] |
| CACNB1 | Zebrafish model | Loss of CACNB1 function leads to paralysis [24] |
| UNC-45B | Human patients | Pathogenic variants cause muscle weakness and progressive congenital myopathy [25] |
| SNC5A | Human patients | Loss-of-function mutations lead to Brugada syndrome and may cause dilated cardiomyopathy [26] |
| MYH3 | - Human patients - MYH3-KO Mice | - Early Downregulation leads to distal arthrogryposis and myosin myopathy [27] - Reduced body weight, muscle weight, myofiber size, and grip strength [28] |
| MYH7 | Human patients | Mutations can lead to hypertrophic cardiomyopathy, skeletal myopathies and congenital or early infantile weakness [29] |
| MYH8 | Human patients | Missense mutation causes distal arthrogryposis and cardiac myxomas [30] |
| MYBPC1 | - Mybpc1 knockout mice - Human patients | - Early postnatal lethality, impaired skeletal muscle formation and structure, skeletal deformity, and respiratory failure [31] - Recessive loss-of-function mutation leads to lethal congenital contractual syndrome [32] |

**Supplementary Figures**


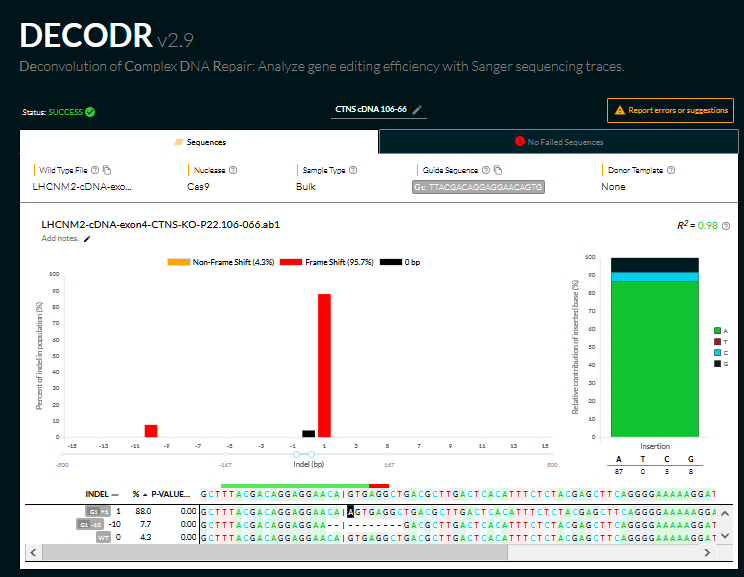

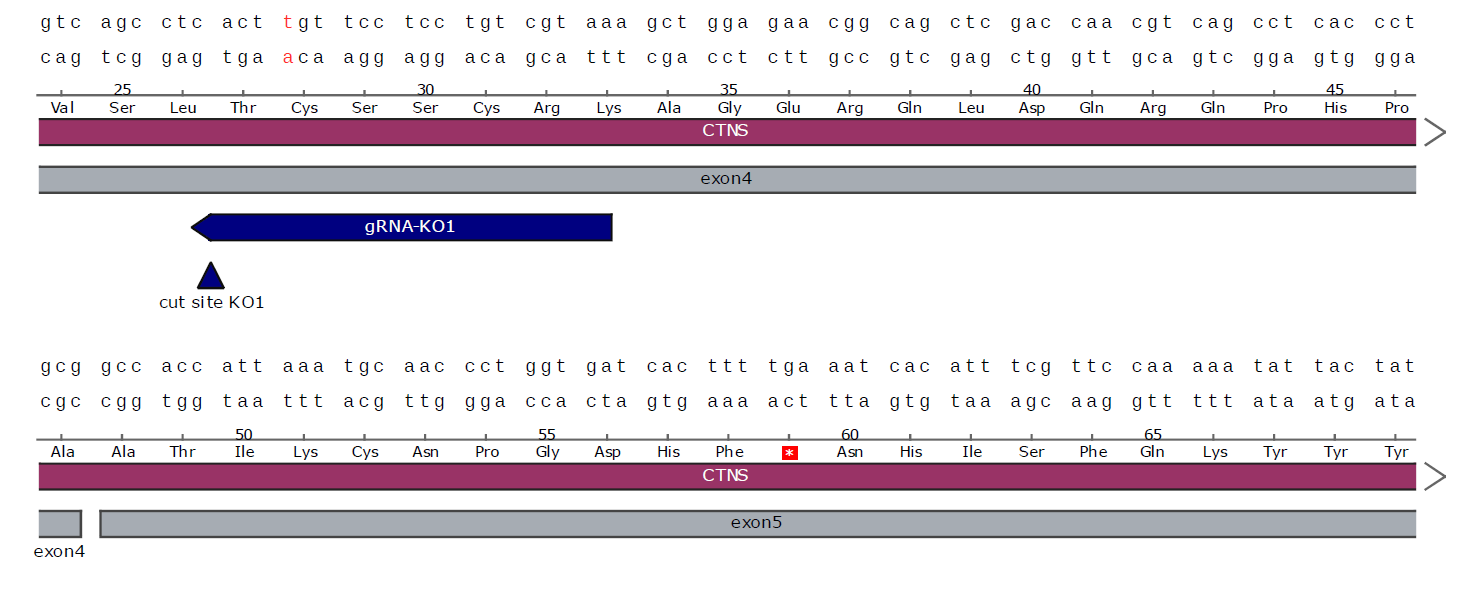


**(A)**

**(B)**

***Supplementary Figure 1. Validation of CTNS knock-out in immortalized human myoblasts at cDNA level****. (****A****)* *Quantification of indel events in CRISPR-edited CTNS^-/-^ myoblasts at cDNA level using DECODR analysis. (****B****) Modification and predicted stop codon (*) in exon 5 resulting in a 58 AA truncated protein (SnapGene).*

***
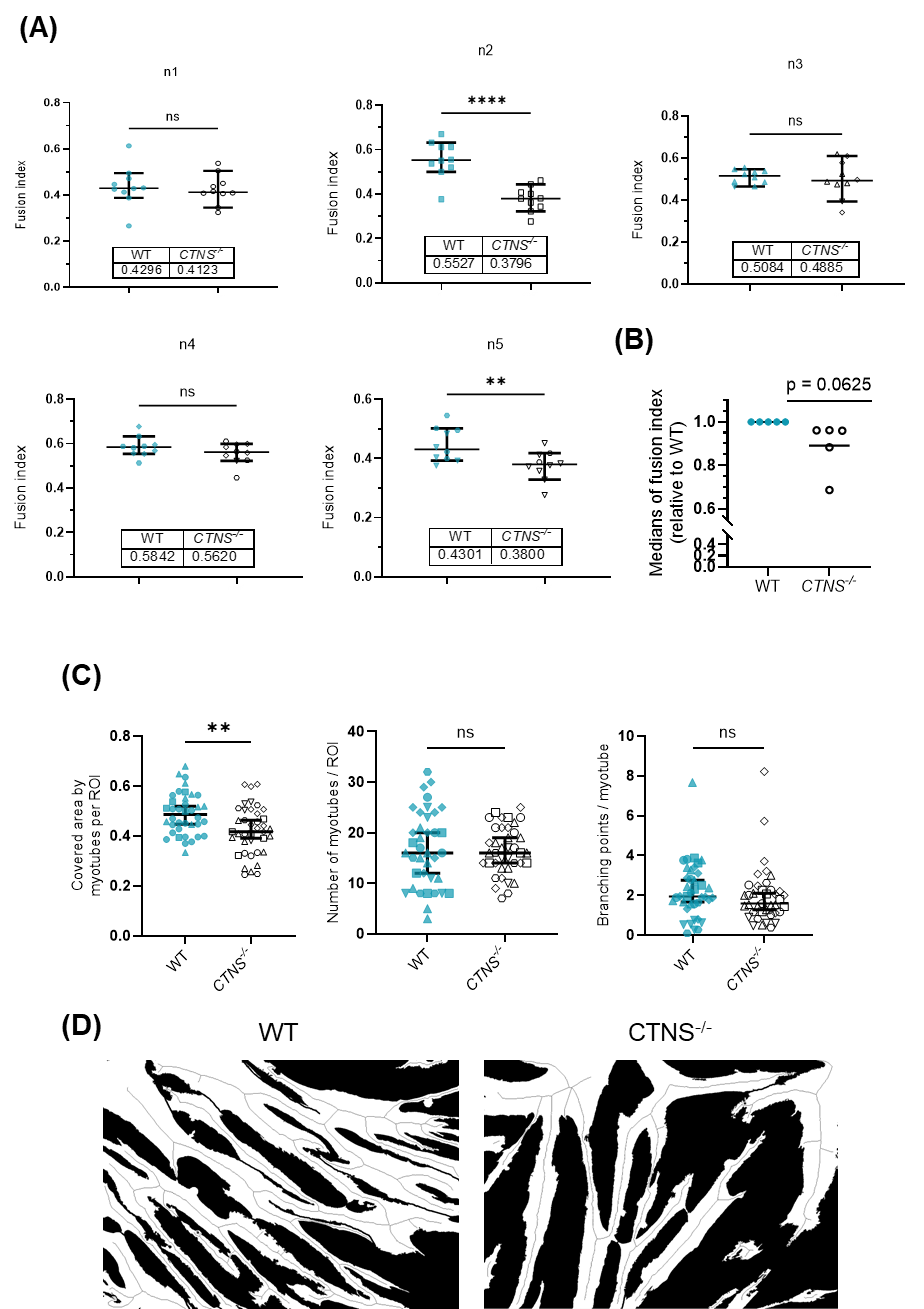
***

***Supplementary Figure 2. Analysis of WT and CTNS^-/-^ myotube differentiation. (A****) Individual plots of each myogenic differentiation experiment (each n) corresponding to the plot of* ***Figure 2D****. Each plot represents 10 ROIs, error bars represent median with 95% CI, numeric values correspond to median. Statistical testing was performed with an unpaired t-test. (****B****)Representation of medians of the 5 replicates of fusion index, normalized to WT. Statistical testing was performed with one sample t and Wilcoxon Signed Rank Test. (****C****) Covered area by myotube per region of interest (ROI), number of myotubes per ROI and branching points per myotube between WT and CTNS^-/-^ day-4 myotubes. Each dot represents an individual image field, data are show the median with 95% CI (n=5). **, p-value<0.01; ns, non-significant, p-value>0.05. Statistical testing was performed with an unpaired t-test. (D) Binary mask images and branching points of representative images of WT and CTNS^-/-^ day-4 myotubes.*

***
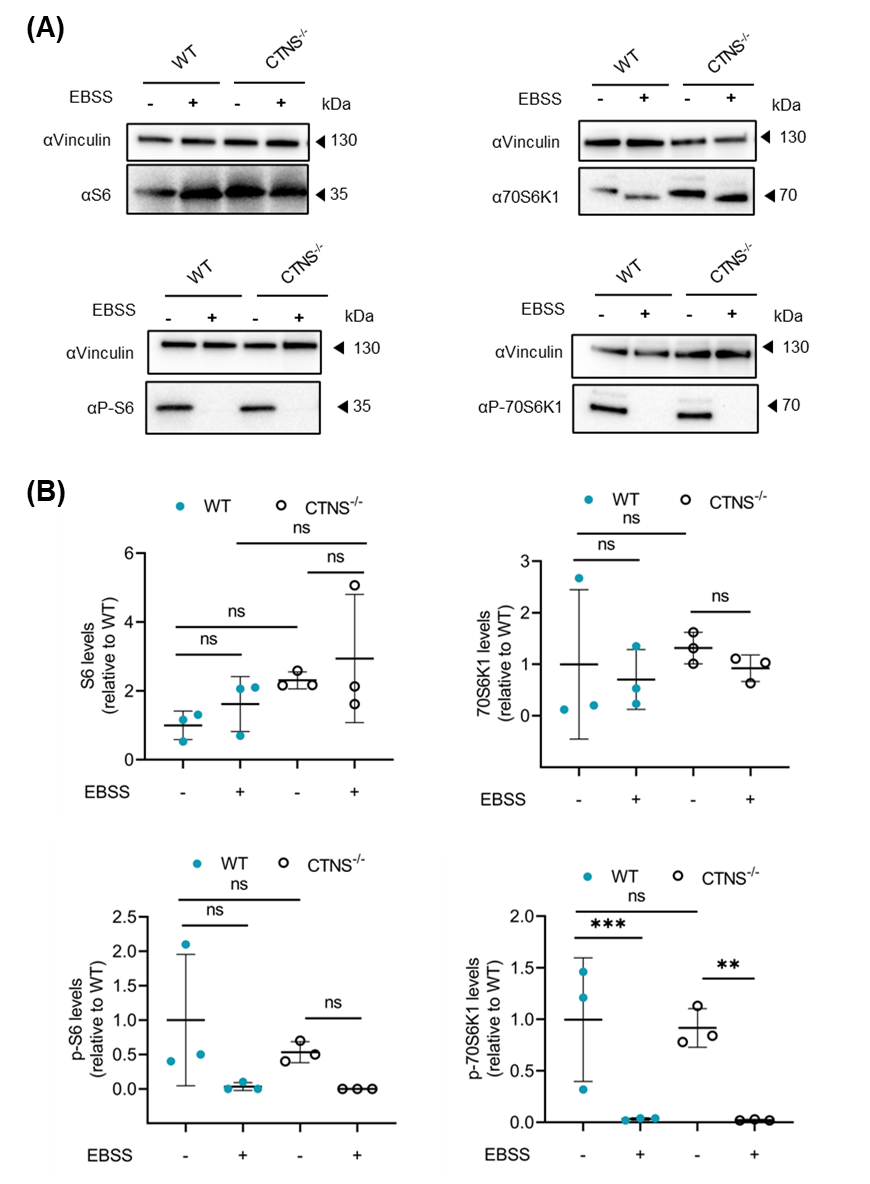
***

***Supplementary Figure 3. Analysis substrates of the mTOR pathway in WT and CTNS^-/-^ myoblasts.*** *(****A****) Representative western blot analysis of (p)S6 and (p)70S6K1 protein expression in WT and CTNS^-/-^ myoblasts under different feeding conditions. 4h incubation with EBSS was used as the starvation condition. Samples normalized for total proteins of vinculin. (****B****) Quantification of (p)S6 and (p)70S6K1 protein expression in WT and CTNS^-/-^ myoblasts (n=3 independent experiments). Samples normalized for total proteins of vinculin. Statistical testing was performed with a one-way Anova, Sidak’s multiple comparison test. ***, p-value<0.001; **, p-value<0.01; ns, non-significant, p>0.05.*


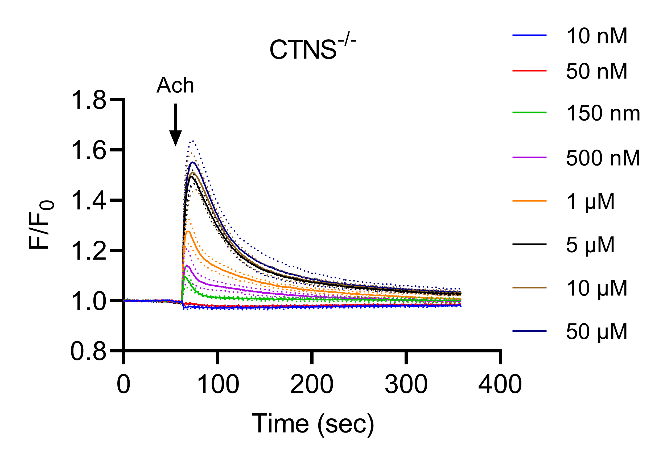

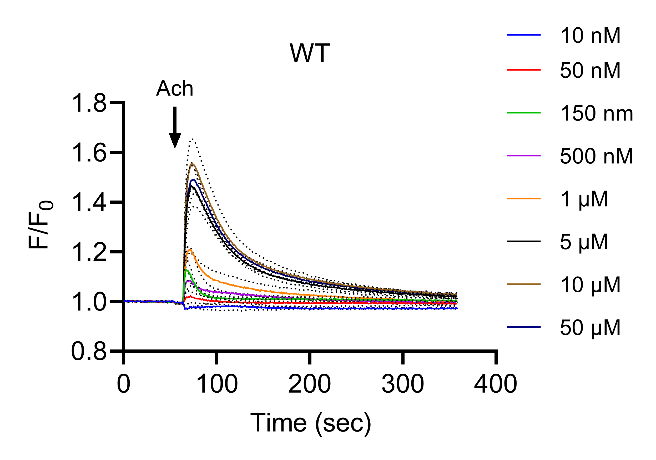

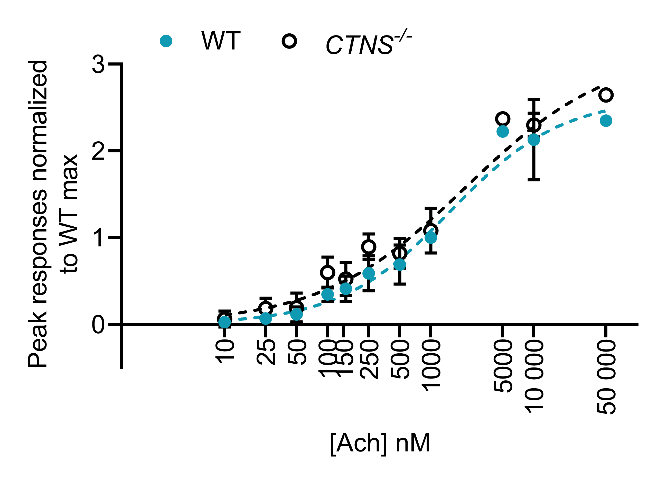


**(A)**

**(B)**

**(C)**

***Supplementary Figure 4. The RyR-mediated Ca^2+^ release remains unaltered in CTNS^-/-^ myotubes. (A-B)*** *Representative intracellular Ca^2+^ measurement of 4-day differentiated WT* ***(A)*** *and CTNS^-/-^* ***(B)*** *myotubes after the addition of different (color coded) concentrations of Ach (M) (n=5). Dotted lines represent error bars.* ***(C)*** *Peak response of Ca^2+^ release after the addition of a concentration of Ach (nM) in WT and CTNS^-/-^ 4-day differentiated myotubes. Data is represented as mean ± SD (n=5 independent experiments).*


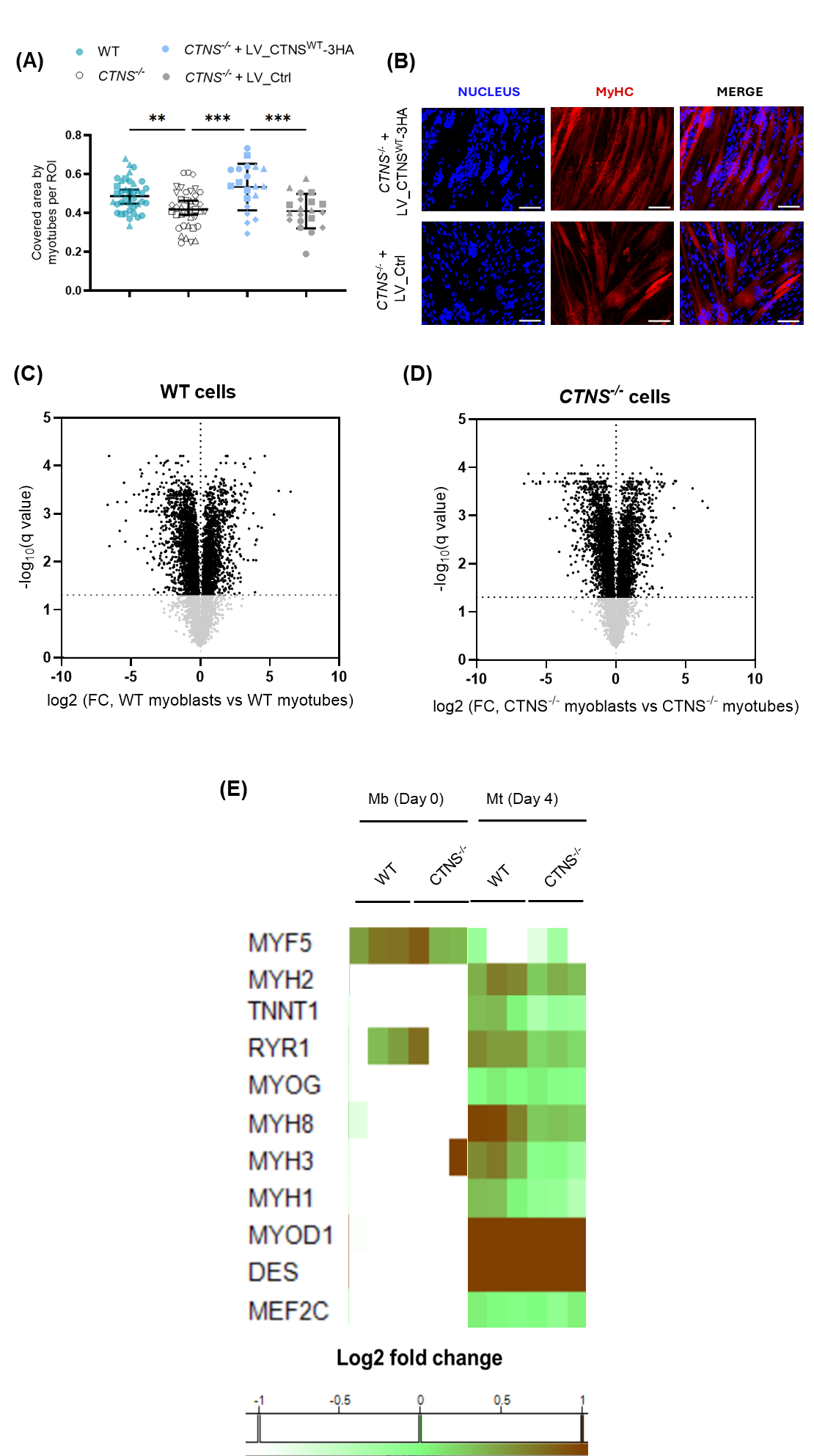


***Supplementary Figure 5****.* ***Covered area by myotube rescue and proteome changes in WT and CTNS^-/-^ cells during differentiation. (A)*** *Covered area by myotubes* *of WT, CTNS^-/-^, and CTNS^-/-^ complemented with either LV_CTNS^WT^-3HA or LV_Ctrl represented by bar plots with each dot representing an individual image field. The data for WT and CTNS^-/-^ are the same datapoints as in* ***Supplementary Figure 2C****. Data are presented as median with 95% CI of n=6 (for WT and CTNS^-/-^) and n=4 (for CTNS^-/-^ + LV addbacks) independent myogenic differentiations (plotted in different shapes). Statistical testing was performed with one-way ANOVA, Sidak’s multiple comparison test. LV, lentiviral vector; WT, wild-type; **, p-value<0.01; ***, p-value<0.001.* ***(B)*** *Representative immunofluorescence images of CTNS-/- + LV_CTNSWT-3HA/LV_Ctrl myotubes after 4 days of myogenic differentiation. Staining by nucleus (DAPI, blue), MyHC (red) and merged. Scale bar: 100 µm.* ***(C)*** *Volcano plot of altered proteins in WT cells between day 0 (Mb) and day 4 (Mt).* ***(D)*** *Volcano plot of altered proteins in CTNS^-/-^ cells between day 0 (Mb) and day 4 (Mt). The thresholds correspond to a q-value<0.05 with the multiple t test using Two-stage step-up method of Benjamin, Krienger, and Yekutieli False Discovery Rate (FDR) approach.* ***(E****)* *Heatmap of myoblast (MYF5) or myotube (RYR1, MYH1, MYH3, MYH8, MEF2C, MYOG, TNNT1, MYOD1, DES, MYH2, DMD) specific markers specifically increased or decreased upon myotube differentiation [33-41]. In white, non-detected data; n = 3.*


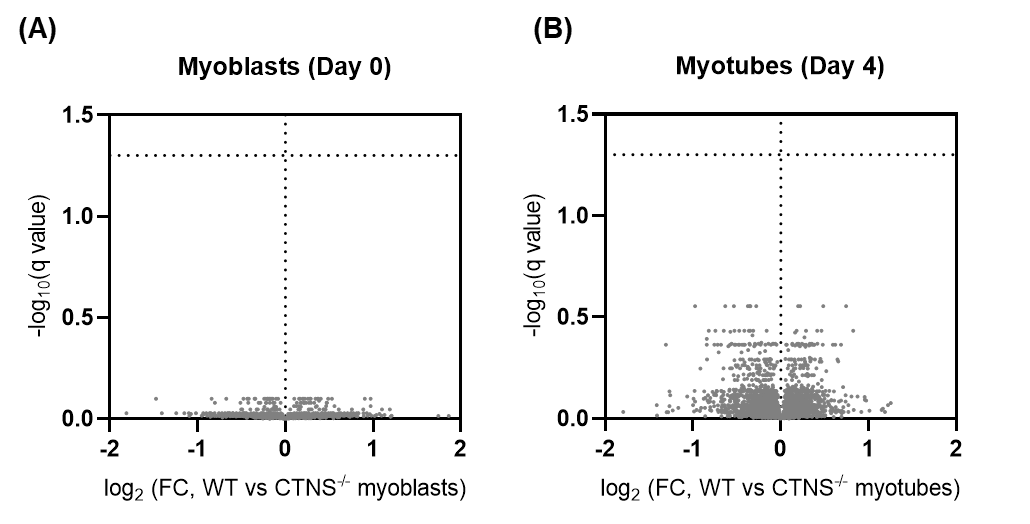
***Supplementary Figure 6. Volcano plots of proteomic analysis in myoblasts and myotubes between WT and CTNS^-/-^ conditions (A)*** *Volcano plot of differential proteins between WT and CNTS^-/-^ cells on day 0 (myoblasts, Mb) and* ***(B)*** *on day 4 (myotubes, Mt). The thresholds correspond to a q-value<0.05 with the multiple t test using Two-stage step-up method of Benjamin, Krienger, and Yekutieli False Discovery Rate (FDR) approach, under which all samples are categorized as non-significant.*

***
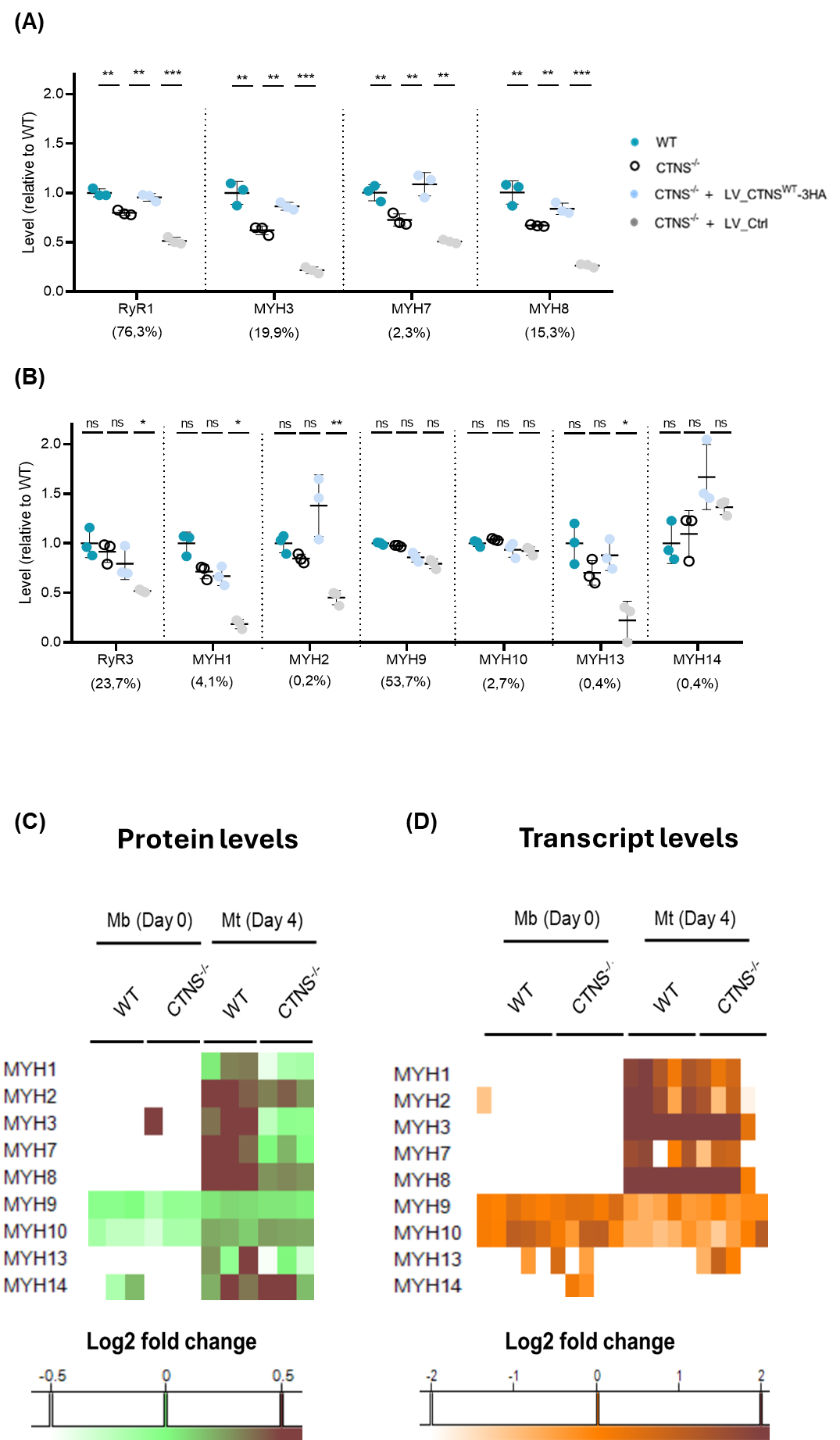
***

***Supplementary Figure 7. Proteomics analysis corroborates previously acquired western blot data*** *(****A****)* *Relative quantitative levels on day 4 between all four conditions of all the RyR and MyHC isoforms identified through DiaPASEF technology. Those isoforms that are part of the cluster affected by CTNS deletion are on top (RyR1, MYH3, MYH7, MYH8) whereas the non-modified isoforms are on the bottom (RyR3, MYH1,2,9,10,13,14); n = 3. Percentages underneath each isoform correspond to the relative abundance of the protein groups in WT cells (counts) in comparison to the total counts of that protein group isoform. * Unadjusted p-value <0.05. ** unadjusted p-value <0.01. *** unadjusted p-value <0.001. ns, non-significant, unadjusted p-value >0.01. (****B****) Heatmap of protein abundance of all the different MyHC isoforms identified between WT and CTNS^-/-^ conditions in myoblasts and myotubes, n = 3. In white, non-detected data (****C****) Transcripts heatmap of all the different MyHC isoforms identified between WT and CTNS^-/-^ conditions in myoblasts and myotubes, n = 4. In white, non-detected data.*

***
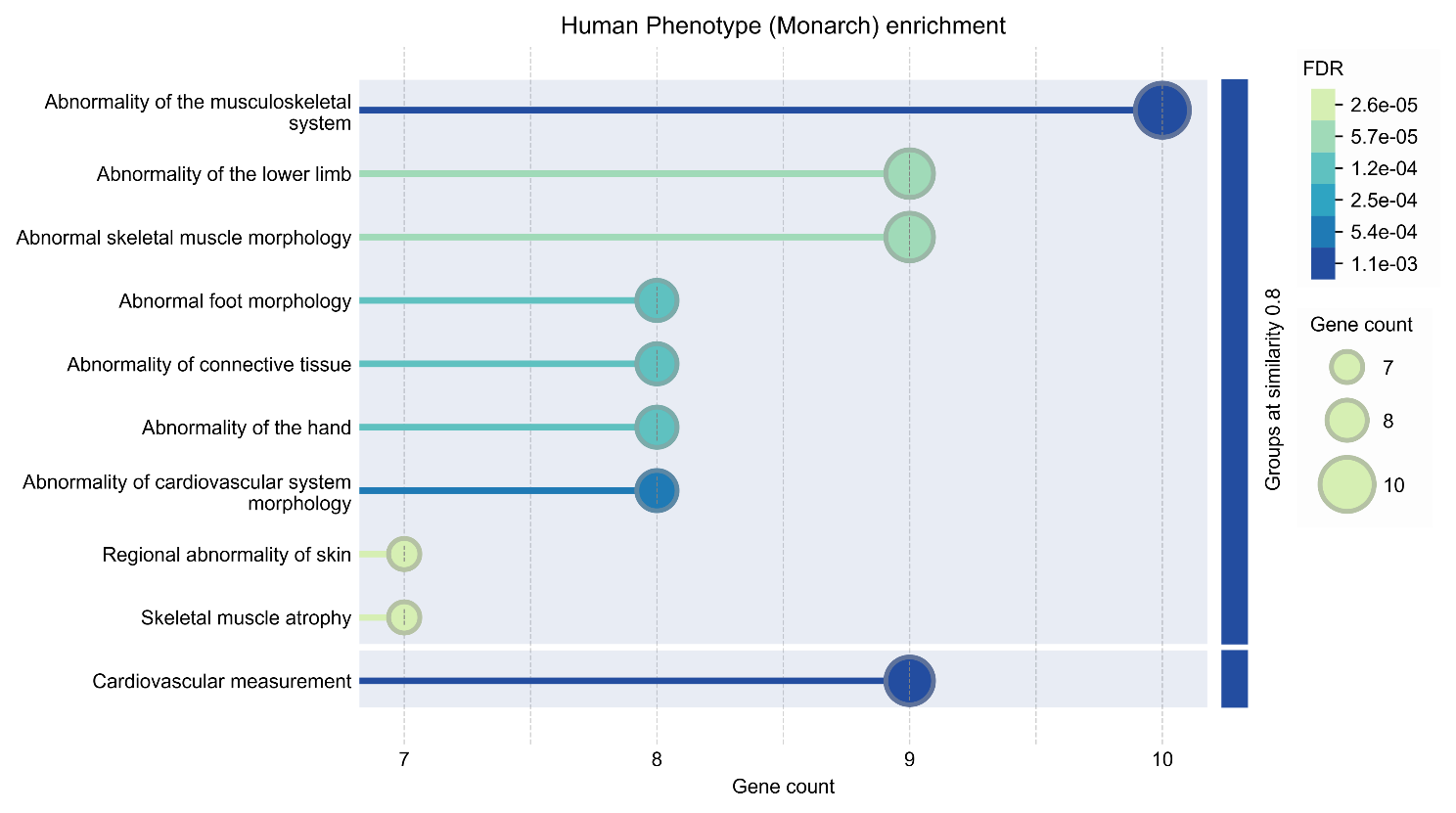
***

***Supplementary Figure 8. Abnormalities associated with alterations in the protein groups from the depleted cluster.*** Human Phenotype (Monarch) enrichment, *obtained from https://string-db.org/.*


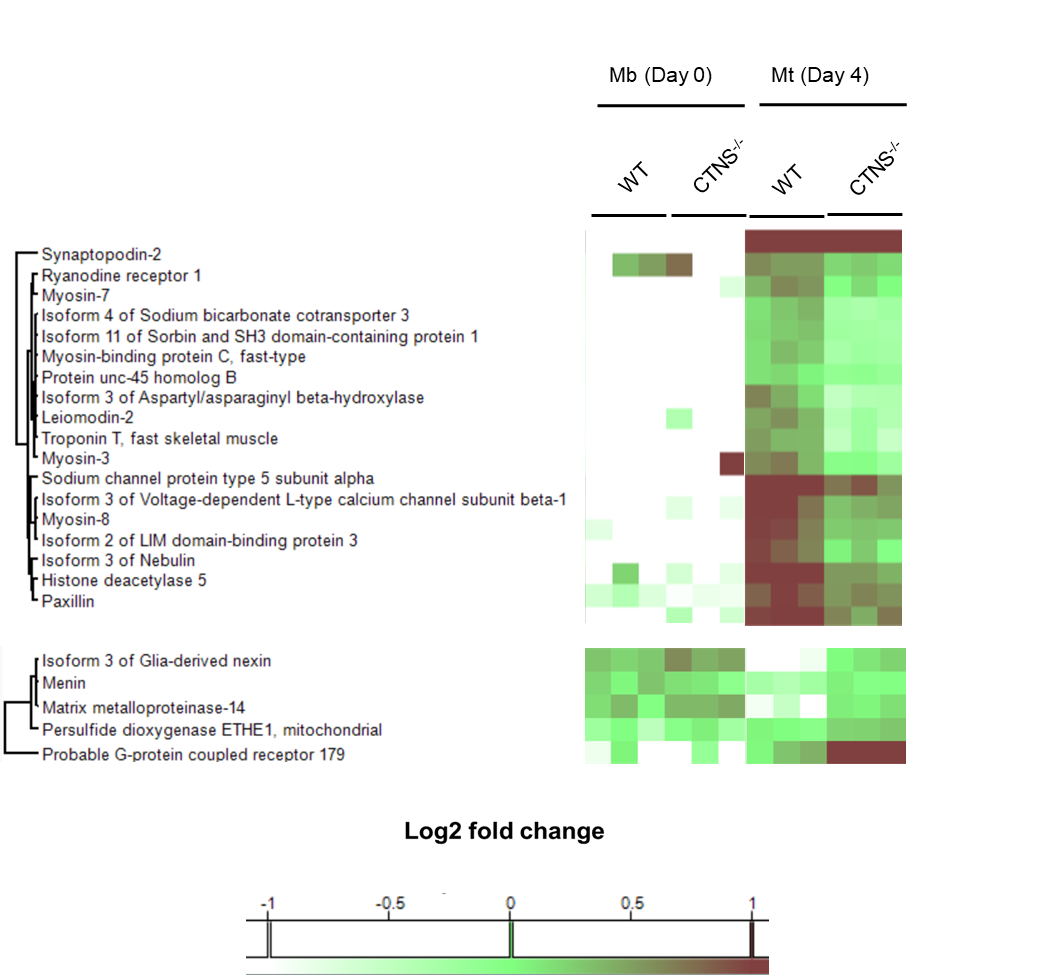


***Supplementary Figure 9.*** *Heatmap of modified proteins. Comparison of protein group levels (log2 fold change from average) between WT and CTNS^-/-^ cells and between day 0 and day 4. Non-detected data is displayed in white; n = 3.*

***
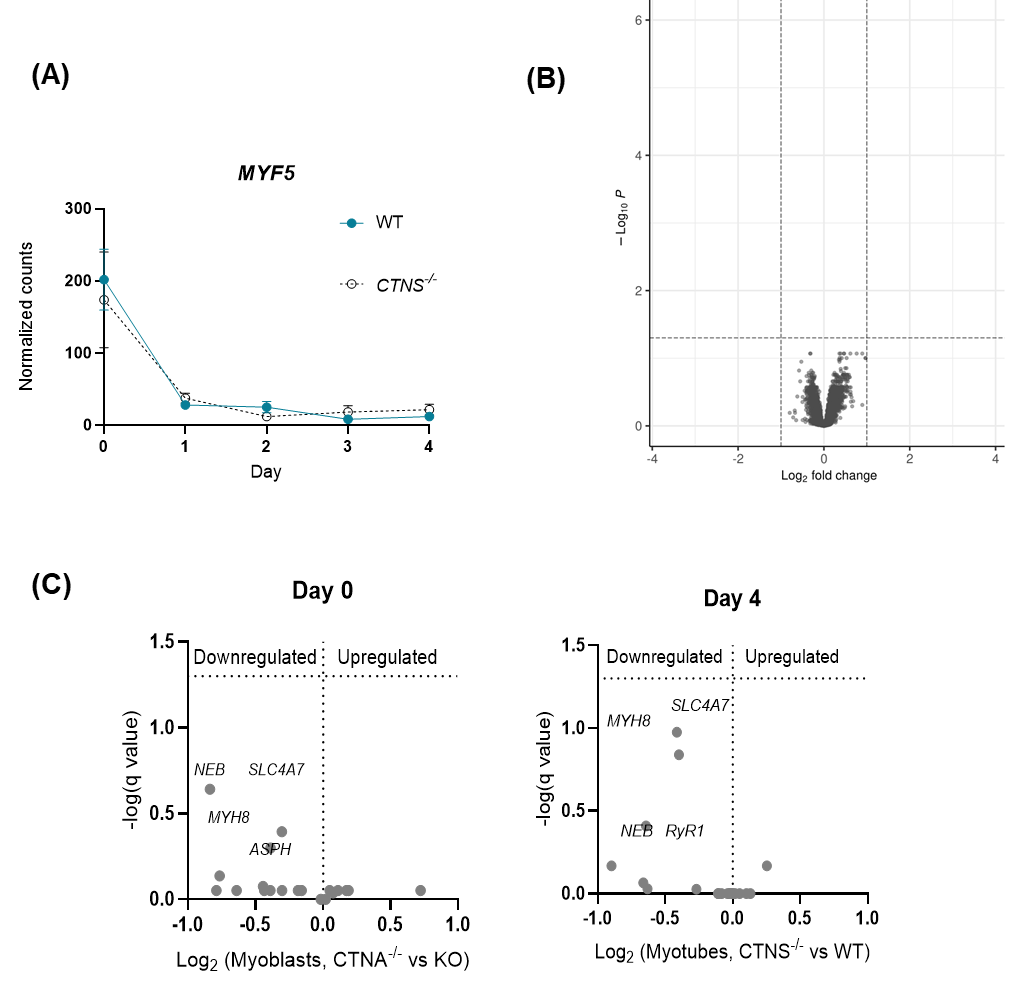
***

***
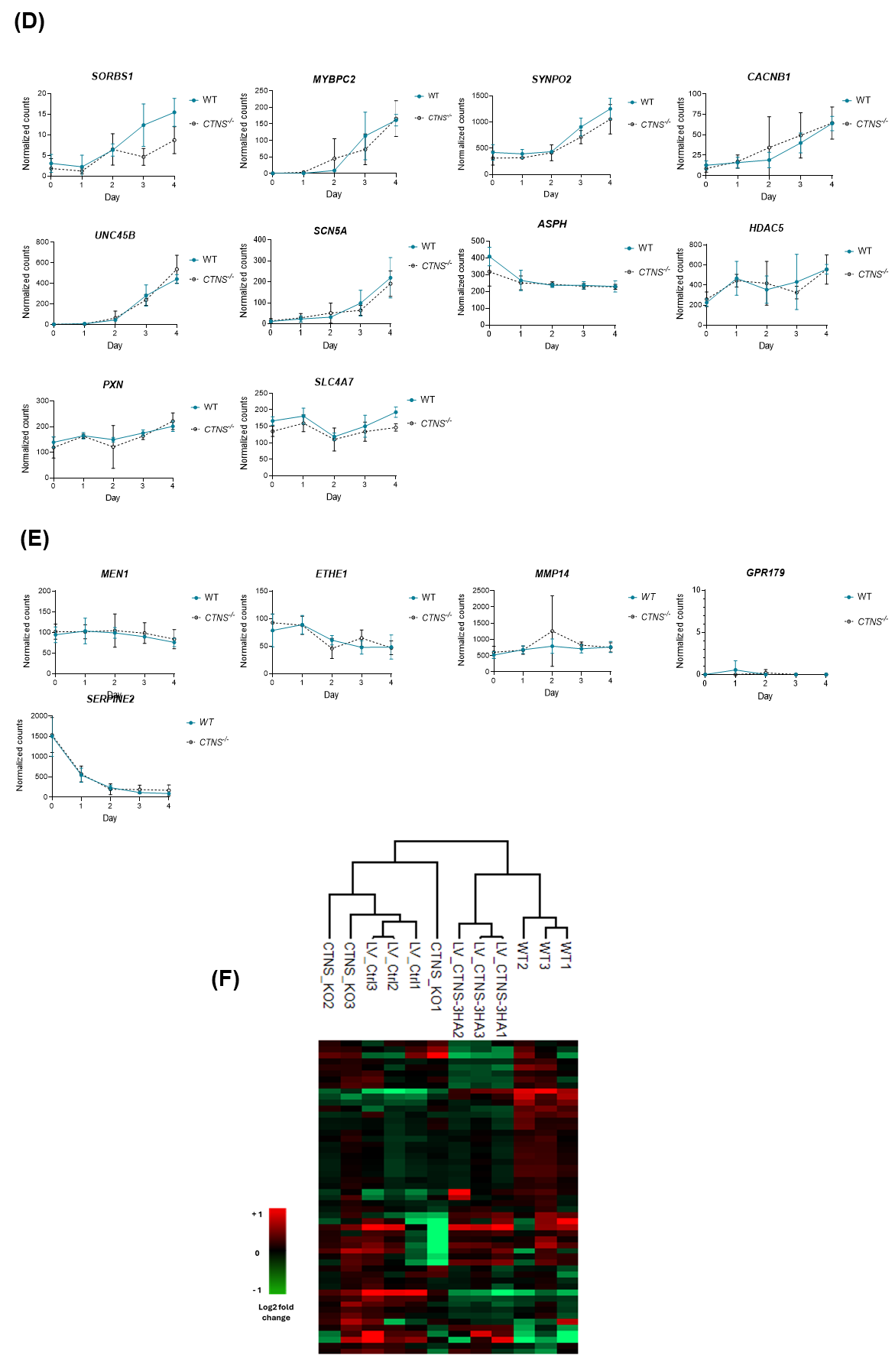
***

***Supplementary Figure 10. Transcriptome and metabolome analysis. (A)*** *Normalized counts of MYF5 mRNA expression during the 4 days of myoblast differentiation for WT and CTNS^-/-^ cells. Data are presented as the mean ± SD (n = 4).* ***(B)*** *Volcano plot of all altered gene expression between WT and CTNS^-/-^ myotubes* ***(C)*** *and for selection of 23 differentially represented protein groups at day 0 and (left) and day 4 (right). The threshold corresponds to q-value<0.05. Reported p-values underwent adjustment for multiple testing using the Benjamini-Hochberg procedure to control the false discovery rate (FDR), thresholds correspond to a q-value < 0.05. (****D****) Normalized counts of myogenesis mRNA expression levels over time on WT and CTNS^-/-^ myoblasts during the 4 days of myotube differentiation for the protein groups identified in the depleted protein network not shown in* ***Figure 8.B*** *and (****E****) for the identified increased protein groups. Data are presented as the mean ± SD, n = 4.* ***(F)*** *Complete unsupervised hierarchical clustering of metabolic levels of day 4 myotubes (n = 3 independent metabolomic extractions).*

**Supplementary references**

1. Mangeot, P.E., et al., *Genome editing in primary cells and in vivo using viral-derived Nanoblades loaded with Cas9-sgRNA ribonucleoproteins.* Nat Commun, 2019. **10**(1): p. 45.

2. Medaer, L., et al., *Residual Cystine Transport Activity for Specific Infantile and Juvenile CTNS Mutations in a PTEC-Based Addback Model.* Cells, 2024. **13**(7).

3. Noe, S., et al., *The Myotube Analyzer: how to assess myogenic features in muscle stem cells.* Skelet Muscle, 2022. **12**(1): p. 12.

4. Ibrahimi, A., et al., *Highly efficient multicistronic lentiviral vectors with peptide 2A sequences.* Hum Gene Ther, 2009. **20**(8): p. 845-60.

5. Fehse, B., et al., *Pois(s)on--it's a question of dose.* Gene Ther, 2004. **11**(11): p. 879-81.

6. HaileMariam, M., et al., *S-Trap, an Ultrafast Sample-Preparation Approach for Shotgun Proteomics.* J Proteome Res, 2018. **17**(9): p. 2917-2924.

7. Skowronek, P., et al., *Rapid and In-Depth Coverage of the (Phospho-)Proteome With Deep Libraries and Optimal Window Design for dia-PASEF.* Mol Cell Proteomics, 2022. **21**(9): p. 100279.

8. Frankenfield, A.M., et al., *Protein Contaminants Matter: Building Universal Protein Contaminant Libraries for DDA and DIA Proteomics.* J Proteome Res, 2022. **21**(9): p. 2104-2113.

9. Demichev, V., et al., *DIA-NN: neural networks and interference correction enable deep proteome coverage in high throughput.* Nat Methods, 2020. **17**(1): p. 41-44.

10. Andrews, S.K., F.; Segonds-Pichon, A.; Biggins, L.; Krueger, C.; Wingett, S. Babraham, *Bioinformatics - FastQC a Quality Control Tool for High Throughput Sequence Data Available online:* [*https://www.bioinformatics.babraham.ac.uk/projects/fastqc/*](https://www.bioinformatics.babraham.ac.uk/projects/fastqc/) *(accessed on 24 March 2024).*

11. Bolger, A.M., M. Lohse, and B. Usadel, *Trimmomatic: a flexible trimmer for Illumina sequence data.* Bioinformatics, 2014. **30**(15): p. 2114-20.

12. Kim, D., et al., *Graph-based genome alignment and genotyping with HISAT2 and HISAT-genotype.* Nat Biotechnol, 2019. **37**(8): p. 907-915.

13. Liao, Y., G.K. Smyth, and W. Shi, *The Subread aligner: fast, accurate and scalable read mapping by seed-and-vote.* Nucleic Acids Res, 2013. **41**(10): p. e108.

14. Love, M.I., W. Huber, and S. Anders, *Moderated estimation of fold change and dispersion for RNA-seq data with DESeq2.* Genome Biol, 2014. **15**(12): p. 550.

15. Vidal, J., et al., *Ryanodine receptor type 1 content decrease-induced endoplasmic reticulum stress is a hallmark of myopathies.* J Cachexia Sarcopenia Muscle, 2023. **14**(6): p. 2882-2897.

16. Pelletier, L., et al., *In vivo RyR1 reduction in muscle triggers a core-like myopathy.* Acta Neuropathol Commun, 2020. **8**(1): p. 192.

17. Sandaradura, S.A., et al., *Nemaline myopathy and distal arthrogryposis associated with an autosomal recessive TNNT3 splice variant.* Hum Mutat, 2018. **39**(3): p. 383-388.

18. Lohanadan, K., et al., *Isoform-specific functions of synaptopodin-2 variants in cytoskeleton stabilization and autophagy regulation in muscle under mechanical stress.* Exp Cell Res, 2021. **408**(2): p. 112865.

19. Bang, M.L., et al., *Nebulin-deficient mice exhibit shorter thin filament lengths and reduced contractile function in skeletal muscle.* J Cell Biol, 2006. **173**(6): p. 905-16.

20. Ahrens-Nicklas, R.C., et al., *Disruption of cardiac thin filament assembly arising from a mutation in LMOD2: A novel mechanism of neonatal dilated cardiomyopathy.* Sci Adv, 2019. **5**(9): p. eaax2066.

21. Vatta, M., et al., *Mutations in Cypher/ZASP in patients with dilated cardiomyopathy and left ventricular non-compaction.* J Am Coll Cardiol, 2003. **42**(11): p. 2014-27.

22. Selcen, D. and A.G. Engel, *Mutations in ZASP define a novel form of muscular dystrophy in humans.* Ann Neurol, 2005. **57**(2): p. 269-76.

23. Koopmann, T.T., et al., *Biallelic loss of LDB3 leads to a lethal pediatric dilated cardiomyopathy.* Eur J Hum Genet, 2023. **31**(1): p. 97-104.

24. Zhou, W., et al., *Non-sense mutations in the dihydropyridine receptor beta1 gene, CACNB1, paralyze zebrafish relaxed mutants.* Cell Calcium, 2006. **39**(3): p. 227-36.

25. Donkervoort, S., et al., *Pathogenic Variants in the Myosin Chaperone UNC-45B Cause Progressive Myopathy with Eccentric Cores.* Am J Hum Genet, 2020. **107**(6): p. 1078-1095.

26. Wilde, A.A.M. and A.S. Amin, *Clinical Spectrum of SCN5A Mutations: Long QT Syndrome, Brugada Syndrome, and Cardiomyopathy.* JACC Clin Electrophysiol, 2018. **4**(5): p. 569-579.

27. Tajsharghi, H., et al., *Embryonic myosin heavy-chain mutations cause distal arthrogryposis and developmental myosin myopathy that persists postnatally.* Arch Neurol, 2008. **65**(8): p. 1083-90.

28. Bharadwaj, A., et al., *Musculoskeletal defects associated with myosin heavy chain-embryonic loss of function are mediated by the YAP signaling pathway.* EMBO Mol Med, 2023. **15**(9): p. e17187.

29. Fiorillo, C., et al., *MYH7-related myopathies: clinical, histopathological and imaging findings in a cohort of Italian patients.* Orphanet J Rare Dis, 2016. **11**(1): p. 91.

30. Veugelers, M., et al., *Mutation of perinatal myosin heavy chain associated with a Carney complex variant.* N Engl J Med, 2004. **351**(5): p. 460-9.

31. Song, T., et al., *Unlocking the Role of sMyBP-C: A Key Player in Skeletal Muscle Development and Growth.* bioRxiv, 2023.

32. Markus, B., et al., *Autosomal recessive lethal congenital contractural syndrome type 4 (LCCS4) caused by a mutation in MYBPC1.* Hum Mutat, 2012. **33**(10): p. 1435-8.

33. Stern-Straeter, J., et al., *Characterization of human myoblast differentiation for tissue-engineering purposes by quantitative gene expression analysis.* J Tissue Eng Regen Med, 2011. **5**(8): p. e197-206.

34. Qiu, K., et al., *Ryanodine receptor RyR1-mediated elevation of Ca(2+) concentration is required for the late stage of myogenic differentiation and fusion.* J Anim Sci Biotechnol, 2022. **13**(1): p. 9.

35. Hindi, L., et al., *Isolation, Culturing, and Differentiation of Primary Myoblasts from Skeletal Muscle of Adult Mice.* Bio Protoc, 2017. **7**(9).

36. Mournetas, V., et al., *Myogenesis modelled by human pluripotent stem cells: a multi-omic study of Duchenne myopathy early onset.* J Cachexia Sarcopenia Muscle, 2021. **12**(1): p. 209-232.

37. Doynova, M.D., et al., *Linkages between changes in the 3D organization of the genome and transcription during myotube differentiation in vitro.* Skelet Muscle, 2017. **7**(1): p. 5.

38. Benavente-Diaz, M., et al., *Dynamics of myogenic differentiation using a novel Myogenin knock-in reporter mouse.* Skelet Muscle, 2021. **11**(1): p. 5.

39. McMahon, D.K., et al., *C2C12 cells: biophysical, biochemical, and immunocytochemical properties.* Am J Physiol, 1994. **266**(6 Pt 1): p. C1795-802.

40. Capetanaki, Y., D.J. Milner, and G. Weitzer, *Desmin in muscle formation and maintenance: knockouts and consequences.* Cell Struct Funct, 1997. **22**(1): p. 103-16.

41. Piasecka, A., et al., *MEF2C shapes the microtranscriptome during differentiation of skeletal muscles.* Sci Rep, 2021. **11**(1): p. 3476.
