# Supplementary figures and images for: An Isogenic Human Myoblast Cell Model for Cystinosis Myopathy Reveals Alteration of Key Myogenic Regulatory Proteins
Source: J Cachexia Sarcopenia Muscle. 2025 Nov 10;16(6):e70116. doi: 10.1002/jcsm.70116 (PMC12598300; doi:10.1002/jcsm.70116)

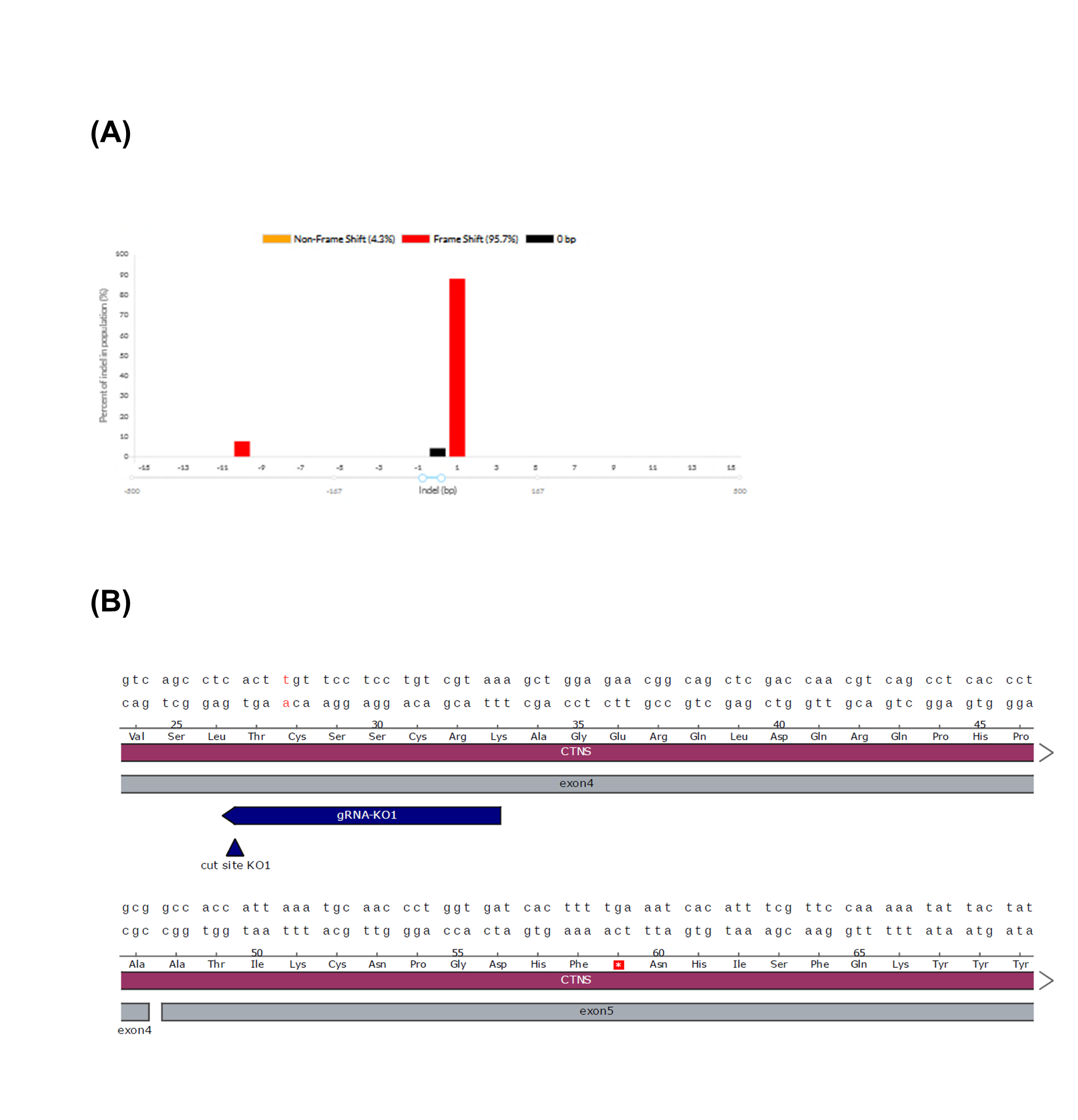

Supplement: Supplementary file 2 — Figure S1: Validation of CTNS knock‐out in immortalized human myoblasts at cDNA level. (A) Quantification of indel events in CRISPR‐edited CTNS−/− myoblasts at cDNA level using DECODR analysis. (B) Modification and predicted stop codon (*) in exon 5 resulting in a 58 AA truncated protein (SnapGene). Figure S2: Analysis of WT and CTNS −/− myotube differentiation. (A) Individual plots of each myogenic differentiation experiment (each n) corresponding to the plot of Figure 2D. Each plot represents 10 ROIs, error bars represent median with 95% CI, numeric values correspond to median. Statistical testing was performed with an unpaired t test. (B)Representation of medians of the five replicates of fusion index, normalized to WT. Statistical testing was performed with one sample t and Wilcoxon signed rank test. (C) Covered area by myotube per region of interest (ROI), number of myotubes per ROI and branching points per myotube between WT and CTNS−/− Day 4 myotubes. Each dot represents an individual image field, data are show the median with 95% CI (n = 5). **p < 0.01; ns, nonsignificant, p > 0.05. Statistical testing was performed with an unpaired t test. (D) Binary mask images and branching points of representative images of WT and CTNS−/− Day 4 myotubes. Figure S3: Analysis substrates of the mTOR pathway in WT and CTNS −/− myoblasts. (A) Representative western blot analysis of (p)S6 and (p)70S6K1 protein expression in WT and CTNS−/− myoblasts under different feeding conditions. 4‐h incubation with EBSS was used as the starvation condition. Samples normalized for total proteins of vinculin. (B) Quantification of (p)S6 and (p)70S6K1 protein expression in WT and CTNS−/− myoblasts (n = 3 independent experiments). Samples normalized for total proteins of vinculin. Statistical testing was performed with a one‐way ANOVA, Sidak's multiple comparison test. ***p < 0.001; **p < 0.01; ns, nonsignificant, p > 0.05. Figure S4: The RyR‐mediated Ca 2+ release remains unaltered in CTNS − [file JCSM-16-e70116-s002.zip › jcsm70116-sup-0002_Supplementary_Figures/SupFig1.png]

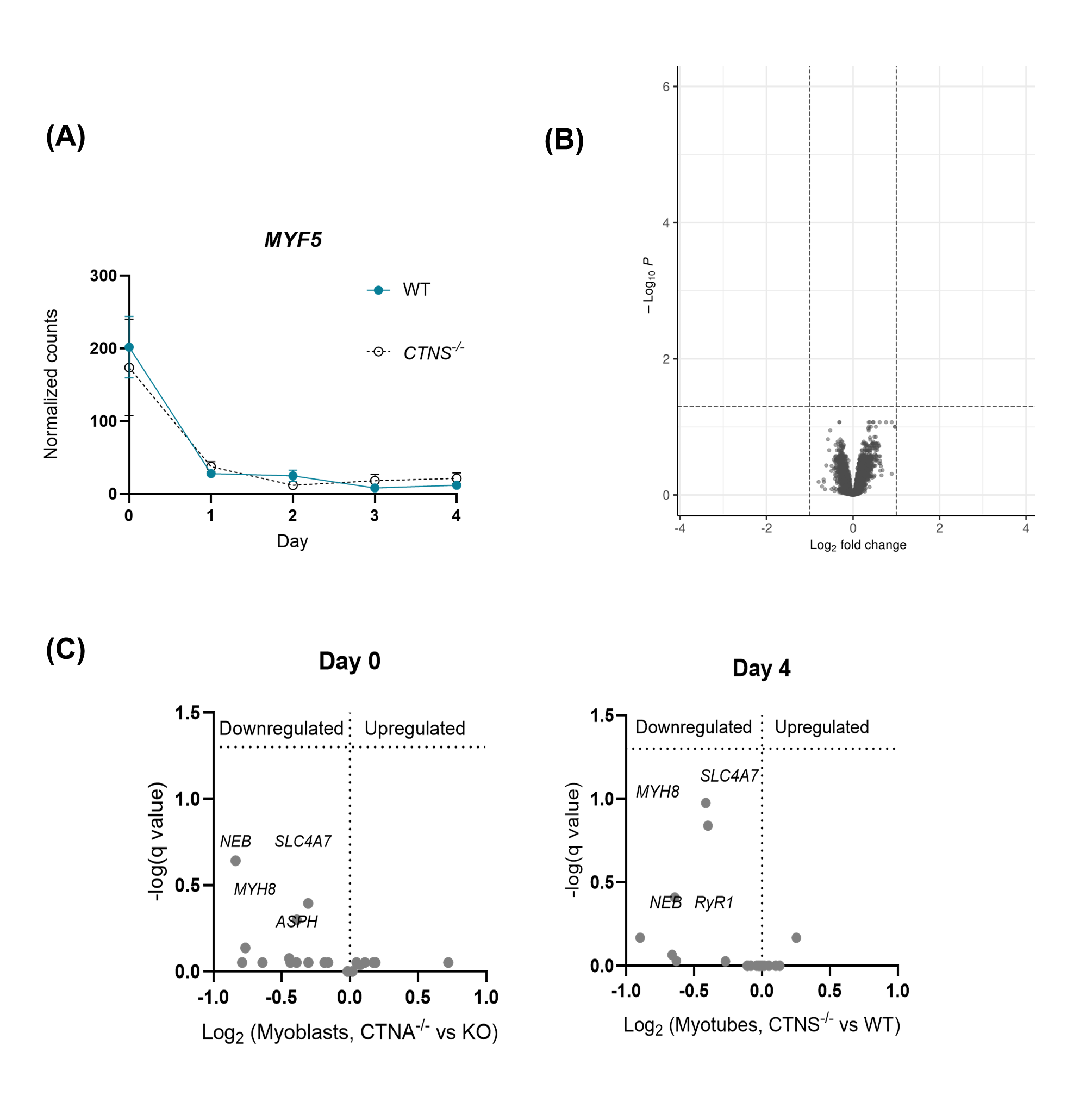

Supplement: Supplementary file 2 — Figure S1: Validation of CTNS knock‐out in immortalized human myoblasts at cDNA level. (A) Quantification of indel events in CRISPR‐edited CTNS−/− myoblasts at cDNA level using DECODR analysis. (B) Modification and predicted stop codon (*) in exon 5 resulting in a 58 AA truncated protein (SnapGene). Figure S2: Analysis of WT and CTNS −/− myotube differentiation. (A) Individual plots of each myogenic differentiation experiment (each n) corresponding to the plot of Figure 2D. Each plot represents 10 ROIs, error bars represent median with 95% CI, numeric values correspond to median. Statistical testing was performed with an unpaired t test. (B)Representation of medians of the five replicates of fusion index, normalized to WT. Statistical testing was performed with one sample t and Wilcoxon signed rank test. (C) Covered area by myotube per region of interest (ROI), number of myotubes per ROI and branching points per myotube between WT and CTNS−/− Day 4 myotubes. Each dot represents an individual image field, data are show the median with 95% CI (n = 5). **p < 0.01; ns, nonsignificant, p > 0.05. Statistical testing was performed with an unpaired t test. (D) Binary mask images and branching points of representative images of WT and CTNS−/− Day 4 myotubes. Figure S3: Analysis substrates of the mTOR pathway in WT and CTNS −/− myoblasts. (A) Representative western blot analysis of (p)S6 and (p)70S6K1 protein expression in WT and CTNS−/− myoblasts under different feeding conditions. 4‐h incubation with EBSS was used as the starvation condition. Samples normalized for total proteins of vinculin. (B) Quantification of (p)S6 and (p)70S6K1 protein expression in WT and CTNS−/− myoblasts (n = 3 independent experiments). Samples normalized for total proteins of vinculin. Statistical testing was performed with a one‐way ANOVA, Sidak's multiple comparison test. ***p < 0.001; **p < 0.01; ns, nonsignificant, p > 0.05. Figure S4: The RyR‐mediated Ca 2+ release remains unaltered in CTNS − [file JCSM-16-e70116-s002.zip › jcsm70116-sup-0002_Supplementary_Figures/SupFig10.1.PNG]

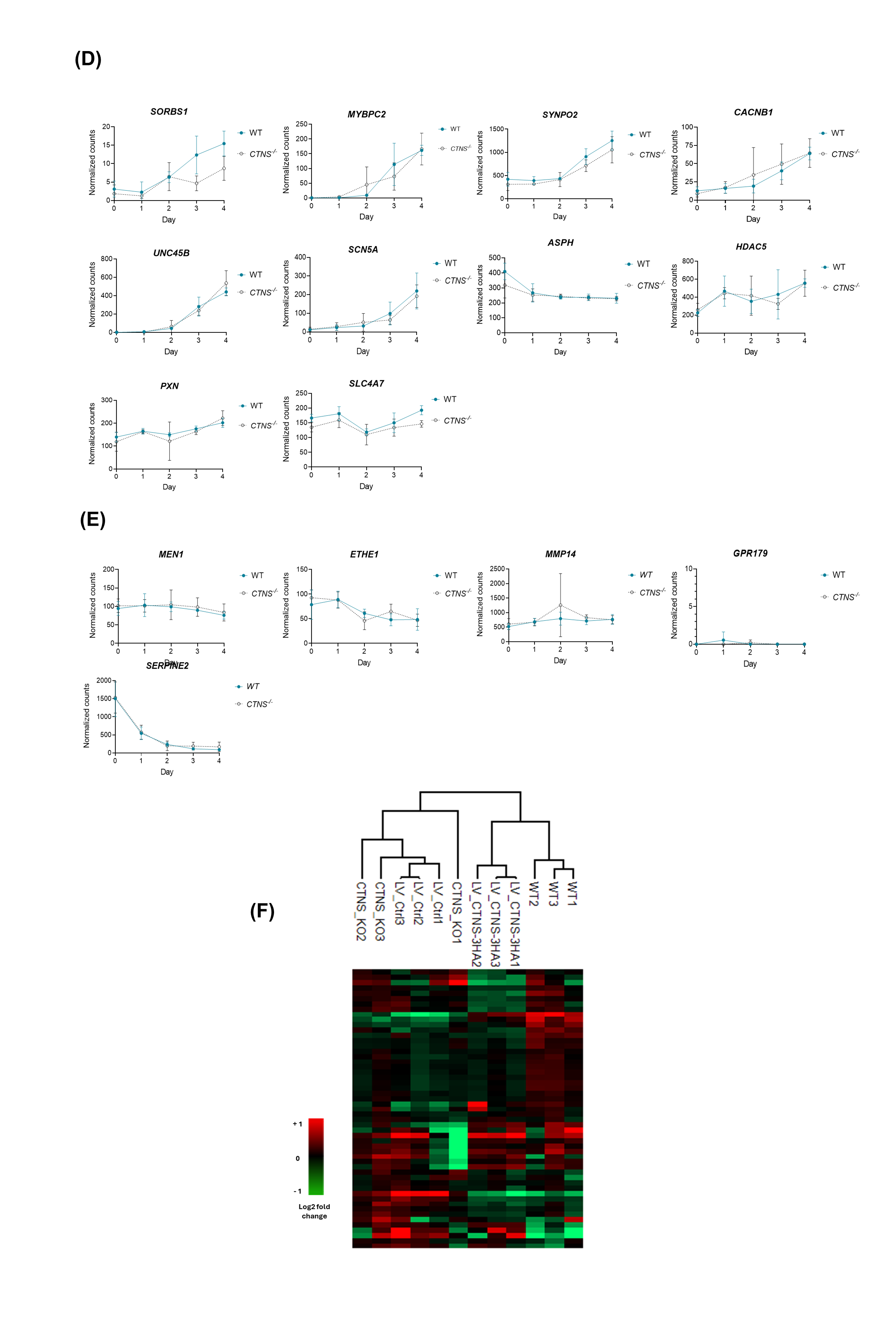

Supplement: Supplementary file 2 — Figure S1: Validation of CTNS knock‐out in immortalized human myoblasts at cDNA level. (A) Quantification of indel events in CRISPR‐edited CTNS−/− myoblasts at cDNA level using DECODR analysis. (B) Modification and predicted stop codon (*) in exon 5 resulting in a 58 AA truncated protein (SnapGene). Figure S2: Analysis of WT and CTNS −/− myotube differentiation. (A) Individual plots of each myogenic differentiation experiment (each n) corresponding to the plot of Figure 2D. Each plot represents 10 ROIs, error bars represent median with 95% CI, numeric values correspond to median. Statistical testing was performed with an unpaired t test. (B)Representation of medians of the five replicates of fusion index, normalized to WT. Statistical testing was performed with one sample t and Wilcoxon signed rank test. (C) Covered area by myotube per region of interest (ROI), number of myotubes per ROI and branching points per myotube between WT and CTNS−/− Day 4 myotubes. Each dot represents an individual image field, data are show the median with 95% CI (n = 5). **p < 0.01; ns, nonsignificant, p > 0.05. Statistical testing was performed with an unpaired t test. (D) Binary mask images and branching points of representative images of WT and CTNS−/− Day 4 myotubes. Figure S3: Analysis substrates of the mTOR pathway in WT and CTNS −/− myoblasts. (A) Representative western blot analysis of (p)S6 and (p)70S6K1 protein expression in WT and CTNS−/− myoblasts under different feeding conditions. 4‐h incubation with EBSS was used as the starvation condition. Samples normalized for total proteins of vinculin. (B) Quantification of (p)S6 and (p)70S6K1 protein expression in WT and CTNS−/− myoblasts (n = 3 independent experiments). Samples normalized for total proteins of vinculin. Statistical testing was performed with a one‐way ANOVA, Sidak's multiple comparison test. ***p < 0.001; **p < 0.01; ns, nonsignificant, p > 0.05. Figure S4: The RyR‐mediated Ca 2+ release remains unaltered in CTNS − [file JCSM-16-e70116-s002.zip › jcsm70116-sup-0002_Supplementary_Figures/SupFig10.2.PNG]

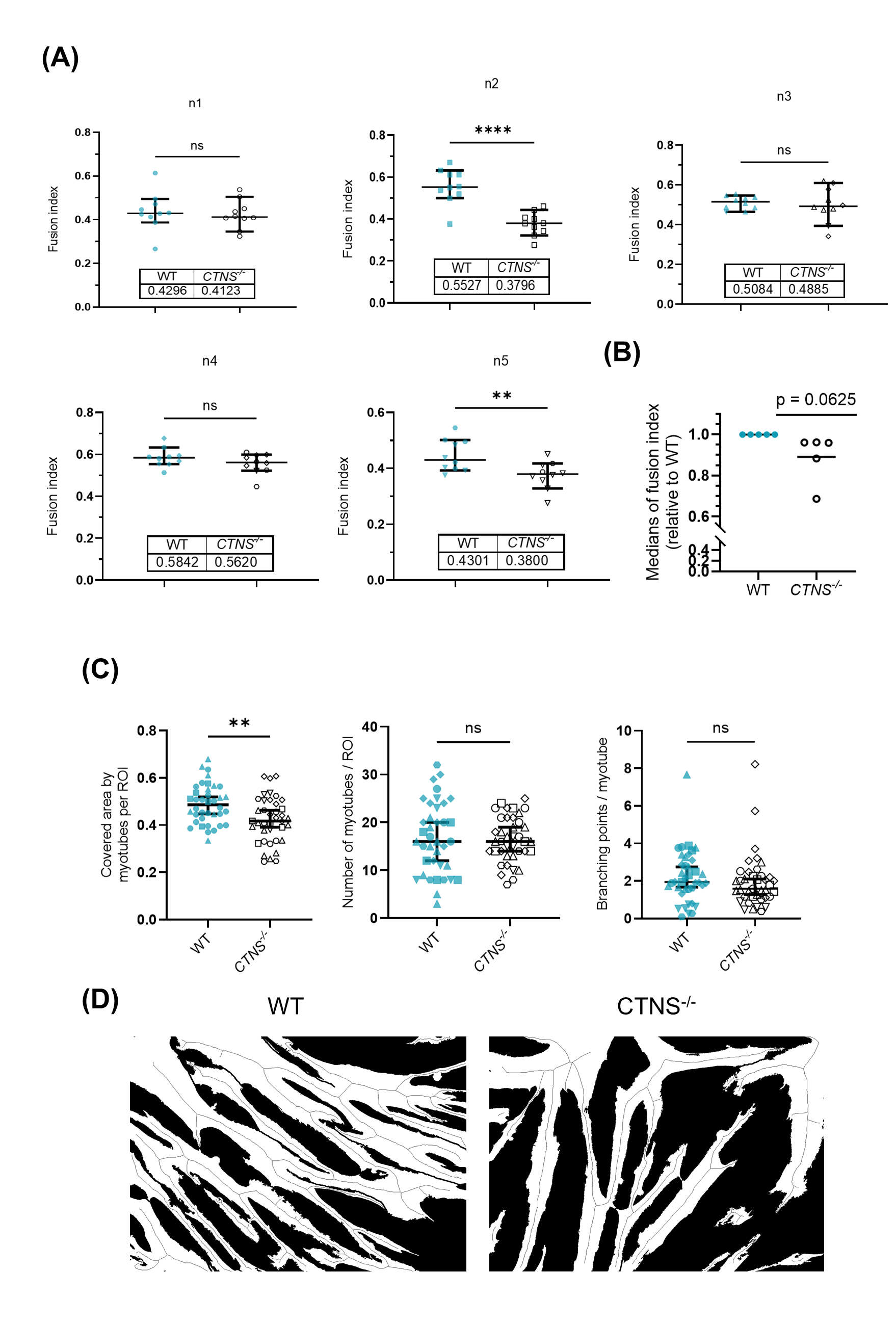

Supplement: Supplementary file 2 — Figure S1: Validation of CTNS knock‐out in immortalized human myoblasts at cDNA level. (A) Quantification of indel events in CRISPR‐edited CTNS−/− myoblasts at cDNA level using DECODR analysis. (B) Modification and predicted stop codon (*) in exon 5 resulting in a 58 AA truncated protein (SnapGene). Figure S2: Analysis of WT and CTNS −/− myotube differentiation. (A) Individual plots of each myogenic differentiation experiment (each n) corresponding to the plot of Figure 2D. Each plot represents 10 ROIs, error bars represent median with 95% CI, numeric values correspond to median. Statistical testing was performed with an unpaired t test. (B)Representation of medians of the five replicates of fusion index, normalized to WT. Statistical testing was performed with one sample t and Wilcoxon signed rank test. (C) Covered area by myotube per region of interest (ROI), number of myotubes per ROI and branching points per myotube between WT and CTNS−/− Day 4 myotubes. Each dot represents an individual image field, data are show the median with 95% CI (n = 5). **p < 0.01; ns, nonsignificant, p > 0.05. Statistical testing was performed with an unpaired t test. (D) Binary mask images and branching points of representative images of WT and CTNS−/− Day 4 myotubes. Figure S3: Analysis substrates of the mTOR pathway in WT and CTNS −/− myoblasts. (A) Representative western blot analysis of (p)S6 and (p)70S6K1 protein expression in WT and CTNS−/− myoblasts under different feeding conditions. 4‐h incubation with EBSS was used as the starvation condition. Samples normalized for total proteins of vinculin. (B) Quantification of (p)S6 and (p)70S6K1 protein expression in WT and CTNS−/− myoblasts (n = 3 independent experiments). Samples normalized for total proteins of vinculin. Statistical testing was performed with a one‐way ANOVA, Sidak's multiple comparison test. ***p < 0.001; **p < 0.01; ns, nonsignificant, p > 0.05. Figure S4: The RyR‐mediated Ca 2+ release remains unaltered in CTNS − [file JCSM-16-e70116-s002.zip › jcsm70116-sup-0002_Supplementary_Figures/SupFig2.PNG]

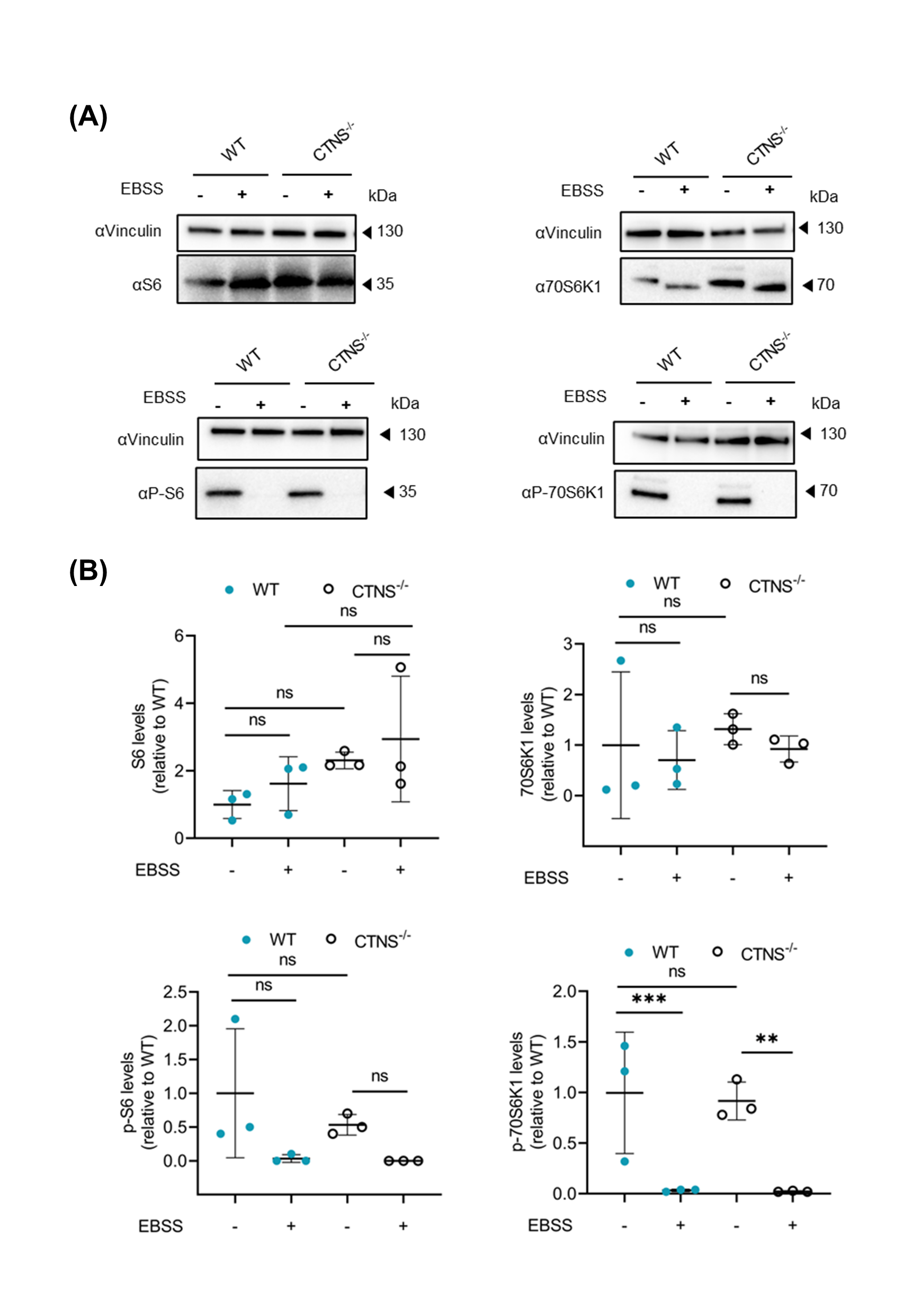

Supplement: Supplementary file 2 — Figure S1: Validation of CTNS knock‐out in immortalized human myoblasts at cDNA level. (A) Quantification of indel events in CRISPR‐edited CTNS−/− myoblasts at cDNA level using DECODR analysis. (B) Modification and predicted stop codon (*) in exon 5 resulting in a 58 AA truncated protein (SnapGene). Figure S2: Analysis of WT and CTNS −/− myotube differentiation. (A) Individual plots of each myogenic differentiation experiment (each n) corresponding to the plot of Figure 2D. Each plot represents 10 ROIs, error bars represent median with 95% CI, numeric values correspond to median. Statistical testing was performed with an unpaired t test. (B)Representation of medians of the five replicates of fusion index, normalized to WT. Statistical testing was performed with one sample t and Wilcoxon signed rank test. (C) Covered area by myotube per region of interest (ROI), number of myotubes per ROI and branching points per myotube between WT and CTNS−/− Day 4 myotubes. Each dot represents an individual image field, data are show the median with 95% CI (n = 5). **p < 0.01; ns, nonsignificant, p > 0.05. Statistical testing was performed with an unpaired t test. (D) Binary mask images and branching points of representative images of WT and CTNS−/− Day 4 myotubes. Figure S3: Analysis substrates of the mTOR pathway in WT and CTNS −/− myoblasts. (A) Representative western blot analysis of (p)S6 and (p)70S6K1 protein expression in WT and CTNS−/− myoblasts under different feeding conditions. 4‐h incubation with EBSS was used as the starvation condition. Samples normalized for total proteins of vinculin. (B) Quantification of (p)S6 and (p)70S6K1 protein expression in WT and CTNS−/− myoblasts (n = 3 independent experiments). Samples normalized for total proteins of vinculin. Statistical testing was performed with a one‐way ANOVA, Sidak's multiple comparison test. ***p < 0.001; **p < 0.01; ns, nonsignificant, p > 0.05. Figure S4: The RyR‐mediated Ca 2+ release remains unaltered in CTNS − [file JCSM-16-e70116-s002.zip › jcsm70116-sup-0002_Supplementary_Figures/SupFig3.PNG]

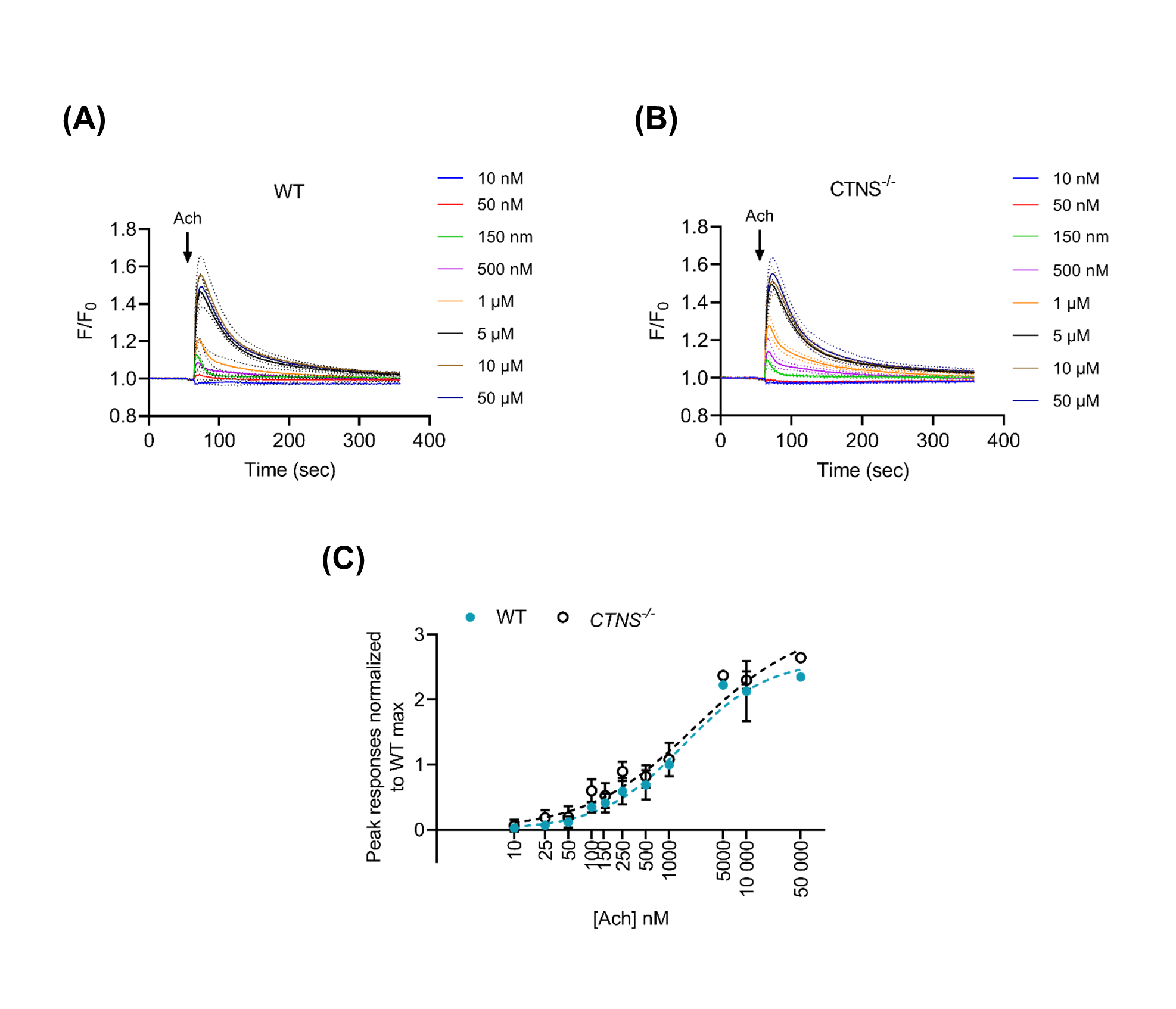

Supplement: Supplementary file 2 — Figure S1: Validation of CTNS knock‐out in immortalized human myoblasts at cDNA level. (A) Quantification of indel events in CRISPR‐edited CTNS−/− myoblasts at cDNA level using DECODR analysis. (B) Modification and predicted stop codon (*) in exon 5 resulting in a 58 AA truncated protein (SnapGene). Figure S2: Analysis of WT and CTNS −/− myotube differentiation. (A) Individual plots of each myogenic differentiation experiment (each n) corresponding to the plot of Figure 2D. Each plot represents 10 ROIs, error bars represent median with 95% CI, numeric values correspond to median. Statistical testing was performed with an unpaired t test. (B)Representation of medians of the five replicates of fusion index, normalized to WT. Statistical testing was performed with one sample t and Wilcoxon signed rank test. (C) Covered area by myotube per region of interest (ROI), number of myotubes per ROI and branching points per myotube between WT and CTNS−/− Day 4 myotubes. Each dot represents an individual image field, data are show the median with 95% CI (n = 5). **p < 0.01; ns, nonsignificant, p > 0.05. Statistical testing was performed with an unpaired t test. (D) Binary mask images and branching points of representative images of WT and CTNS−/− Day 4 myotubes. Figure S3: Analysis substrates of the mTOR pathway in WT and CTNS −/− myoblasts. (A) Representative western blot analysis of (p)S6 and (p)70S6K1 protein expression in WT and CTNS−/− myoblasts under different feeding conditions. 4‐h incubation with EBSS was used as the starvation condition. Samples normalized for total proteins of vinculin. (B) Quantification of (p)S6 and (p)70S6K1 protein expression in WT and CTNS−/− myoblasts (n = 3 independent experiments). Samples normalized for total proteins of vinculin. Statistical testing was performed with a one‐way ANOVA, Sidak's multiple comparison test. ***p < 0.001; **p < 0.01; ns, nonsignificant, p > 0.05. Figure S4: The RyR‐mediated Ca 2+ release remains unaltered in CTNS − [file JCSM-16-e70116-s002.zip › jcsm70116-sup-0002_Supplementary_Figures/SupFig4.PNG]

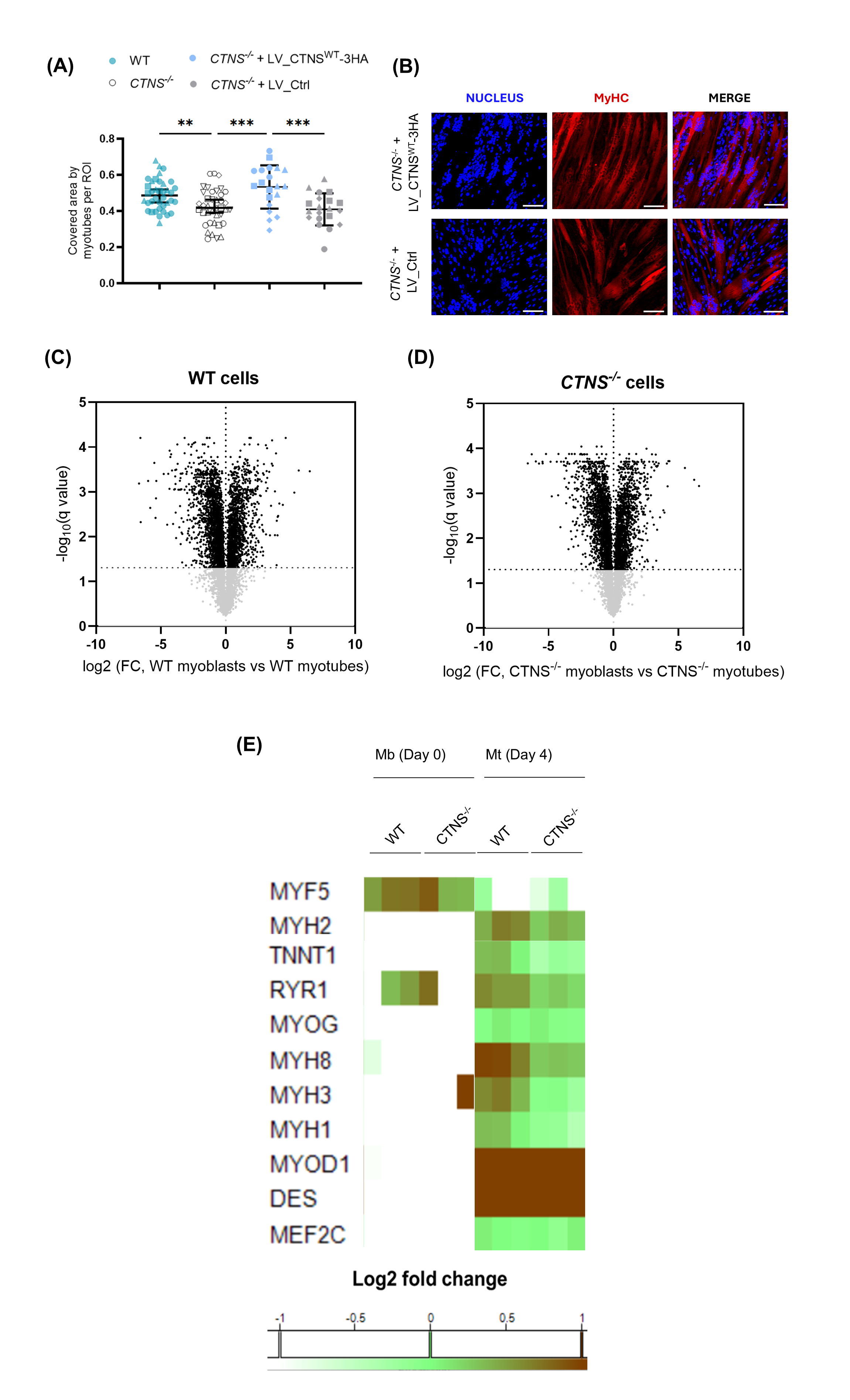

Supplement: Supplementary file 2 — Figure S1: Validation of CTNS knock‐out in immortalized human myoblasts at cDNA level. (A) Quantification of indel events in CRISPR‐edited CTNS−/− myoblasts at cDNA level using DECODR analysis. (B) Modification and predicted stop codon (*) in exon 5 resulting in a 58 AA truncated protein (SnapGene). Figure S2: Analysis of WT and CTNS −/− myotube differentiation. (A) Individual plots of each myogenic differentiation experiment (each n) corresponding to the plot of Figure 2D. Each plot represents 10 ROIs, error bars represent median with 95% CI, numeric values correspond to median. Statistical testing was performed with an unpaired t test. (B)Representation of medians of the five replicates of fusion index, normalized to WT. Statistical testing was performed with one sample t and Wilcoxon signed rank test. (C) Covered area by myotube per region of interest (ROI), number of myotubes per ROI and branching points per myotube between WT and CTNS−/− Day 4 myotubes. Each dot represents an individual image field, data are show the median with 95% CI (n = 5). **p < 0.01; ns, nonsignificant, p > 0.05. Statistical testing was performed with an unpaired t test. (D) Binary mask images and branching points of representative images of WT and CTNS−/− Day 4 myotubes. Figure S3: Analysis substrates of the mTOR pathway in WT and CTNS −/− myoblasts. (A) Representative western blot analysis of (p)S6 and (p)70S6K1 protein expression in WT and CTNS−/− myoblasts under different feeding conditions. 4‐h incubation with EBSS was used as the starvation condition. Samples normalized for total proteins of vinculin. (B) Quantification of (p)S6 and (p)70S6K1 protein expression in WT and CTNS−/− myoblasts (n = 3 independent experiments). Samples normalized for total proteins of vinculin. Statistical testing was performed with a one‐way ANOVA, Sidak's multiple comparison test. ***p < 0.001; **p < 0.01; ns, nonsignificant, p > 0.05. Figure S4: The RyR‐mediated Ca 2+ release remains unaltered in CTNS − [file JCSM-16-e70116-s002.zip › jcsm70116-sup-0002_Supplementary_Figures/SupFig5.PNG]

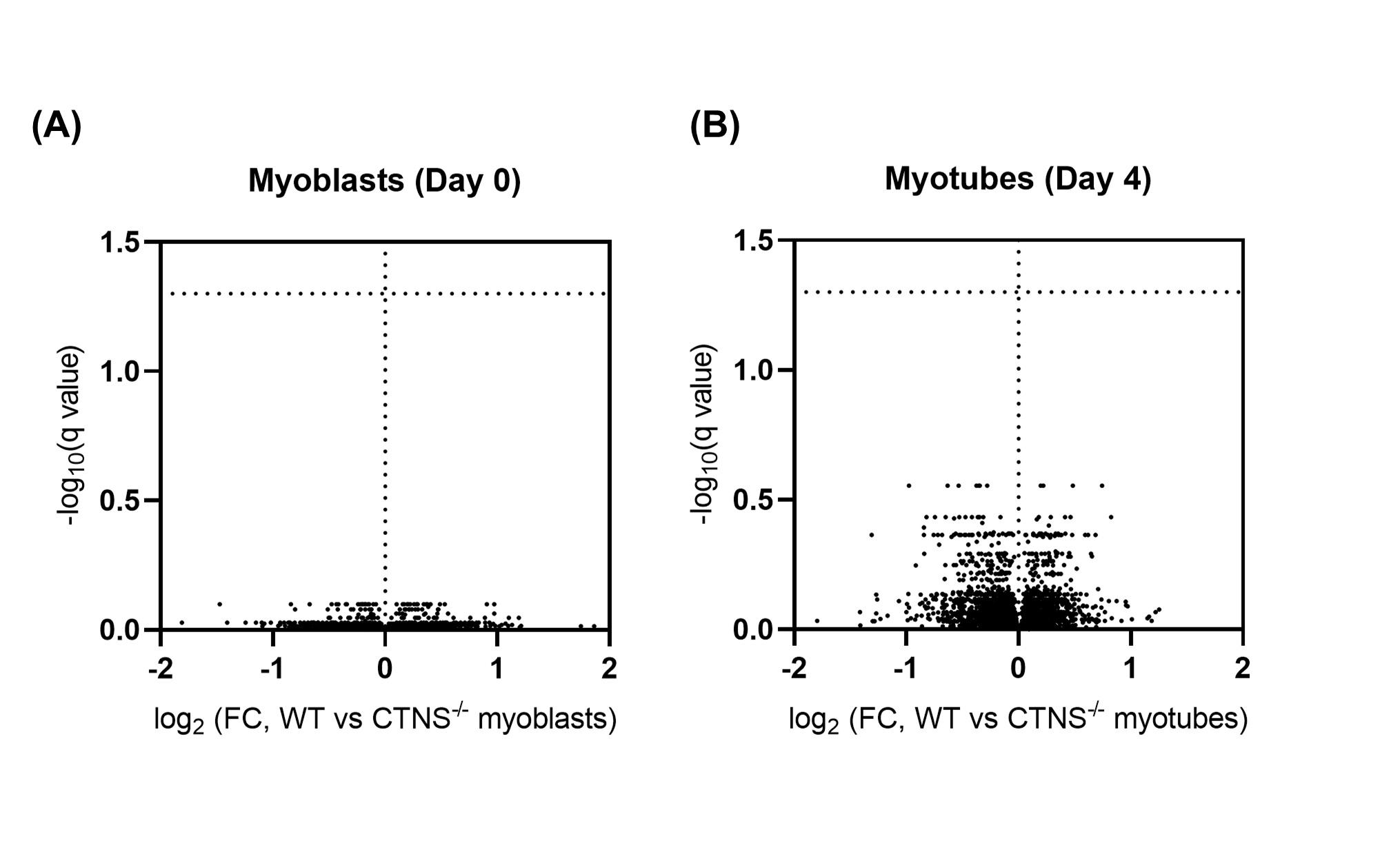

Supplement: Supplementary file 2 — Figure S1: Validation of CTNS knock‐out in immortalized human myoblasts at cDNA level. (A) Quantification of indel events in CRISPR‐edited CTNS−/− myoblasts at cDNA level using DECODR analysis. (B) Modification and predicted stop codon (*) in exon 5 resulting in a 58 AA truncated protein (SnapGene). Figure S2: Analysis of WT and CTNS −/− myotube differentiation. (A) Individual plots of each myogenic differentiation experiment (each n) corresponding to the plot of Figure 2D. Each plot represents 10 ROIs, error bars represent median with 95% CI, numeric values correspond to median. Statistical testing was performed with an unpaired t test. (B)Representation of medians of the five replicates of fusion index, normalized to WT. Statistical testing was performed with one sample t and Wilcoxon signed rank test. (C) Covered area by myotube per region of interest (ROI), number of myotubes per ROI and branching points per myotube between WT and CTNS−/− Day 4 myotubes. Each dot represents an individual image field, data are show the median with 95% CI (n = 5). **p < 0.01; ns, nonsignificant, p > 0.05. Statistical testing was performed with an unpaired t test. (D) Binary mask images and branching points of representative images of WT and CTNS−/− Day 4 myotubes. Figure S3: Analysis substrates of the mTOR pathway in WT and CTNS −/− myoblasts. (A) Representative western blot analysis of (p)S6 and (p)70S6K1 protein expression in WT and CTNS−/− myoblasts under different feeding conditions. 4‐h incubation with EBSS was used as the starvation condition. Samples normalized for total proteins of vinculin. (B) Quantification of (p)S6 and (p)70S6K1 protein expression in WT and CTNS−/− myoblasts (n = 3 independent experiments). Samples normalized for total proteins of vinculin. Statistical testing was performed with a one‐way ANOVA, Sidak's multiple comparison test. ***p < 0.001; **p < 0.01; ns, nonsignificant, p > 0.05. Figure S4: The RyR‐mediated Ca 2+ release remains unaltered in CTNS − [file JCSM-16-e70116-s002.zip › jcsm70116-sup-0002_Supplementary_Figures/SupFig6.PNG]

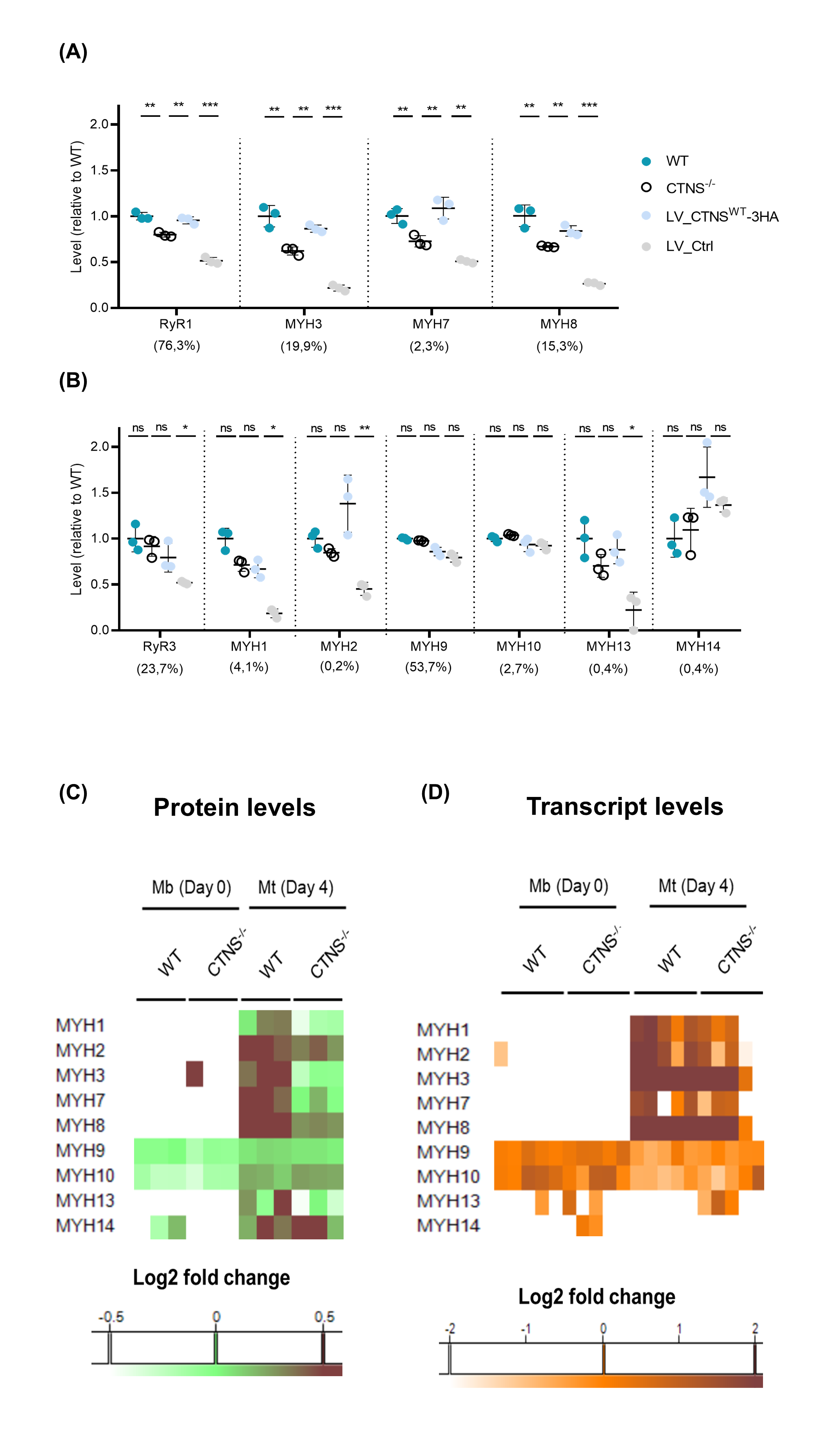

Supplement: Supplementary file 2 — Figure S1: Validation of CTNS knock‐out in immortalized human myoblasts at cDNA level. (A) Quantification of indel events in CRISPR‐edited CTNS−/− myoblasts at cDNA level using DECODR analysis. (B) Modification and predicted stop codon (*) in exon 5 resulting in a 58 AA truncated protein (SnapGene). Figure S2: Analysis of WT and CTNS −/− myotube differentiation. (A) Individual plots of each myogenic differentiation experiment (each n) corresponding to the plot of Figure 2D. Each plot represents 10 ROIs, error bars represent median with 95% CI, numeric values correspond to median. Statistical testing was performed with an unpaired t test. (B)Representation of medians of the five replicates of fusion index, normalized to WT. Statistical testing was performed with one sample t and Wilcoxon signed rank test. (C) Covered area by myotube per region of interest (ROI), number of myotubes per ROI and branching points per myotube between WT and CTNS−/− Day 4 myotubes. Each dot represents an individual image field, data are show the median with 95% CI (n = 5). **p < 0.01; ns, nonsignificant, p > 0.05. Statistical testing was performed with an unpaired t test. (D) Binary mask images and branching points of representative images of WT and CTNS−/− Day 4 myotubes. Figure S3: Analysis substrates of the mTOR pathway in WT and CTNS −/− myoblasts. (A) Representative western blot analysis of (p)S6 and (p)70S6K1 protein expression in WT and CTNS−/− myoblasts under different feeding conditions. 4‐h incubation with EBSS was used as the starvation condition. Samples normalized for total proteins of vinculin. (B) Quantification of (p)S6 and (p)70S6K1 protein expression in WT and CTNS−/− myoblasts (n = 3 independent experiments). Samples normalized for total proteins of vinculin. Statistical testing was performed with a one‐way ANOVA, Sidak's multiple comparison test. ***p < 0.001; **p < 0.01; ns, nonsignificant, p > 0.05. Figure S4: The RyR‐mediated Ca 2+ release remains unaltered in CTNS − [file JCSM-16-e70116-s002.zip › jcsm70116-sup-0002_Supplementary_Figures/SupFig7.PNG]

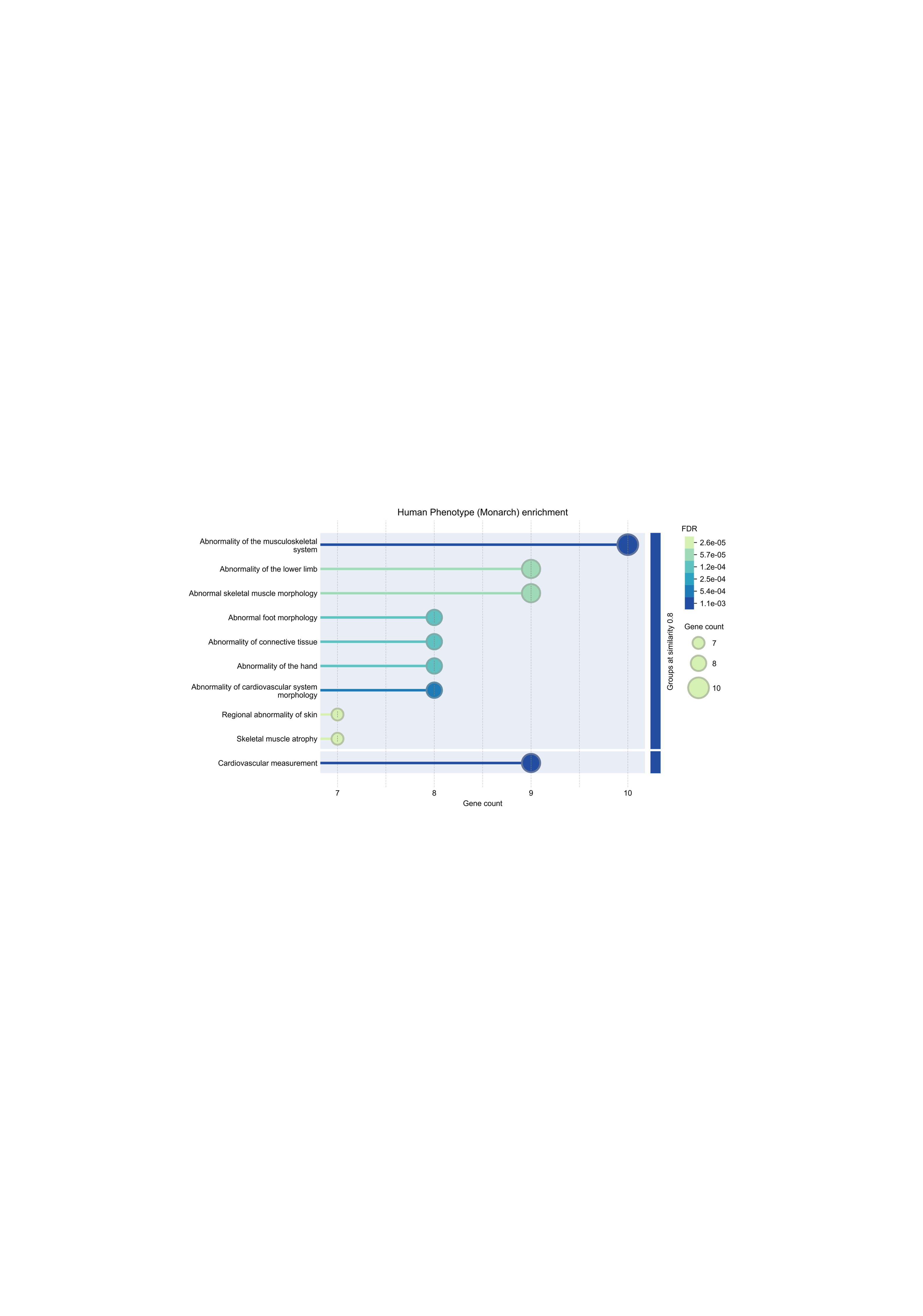

Supplement: Supplementary file 2 — Figure S1: Validation of CTNS knock‐out in immortalized human myoblasts at cDNA level. (A) Quantification of indel events in CRISPR‐edited CTNS−/− myoblasts at cDNA level using DECODR analysis. (B) Modification and predicted stop codon (*) in exon 5 resulting in a 58 AA truncated protein (SnapGene). Figure S2: Analysis of WT and CTNS −/− myotube differentiation. (A) Individual plots of each myogenic differentiation experiment (each n) corresponding to the plot of Figure 2D. Each plot represents 10 ROIs, error bars represent median with 95% CI, numeric values correspond to median. Statistical testing was performed with an unpaired t test. (B)Representation of medians of the five replicates of fusion index, normalized to WT. Statistical testing was performed with one sample t and Wilcoxon signed rank test. (C) Covered area by myotube per region of interest (ROI), number of myotubes per ROI and branching points per myotube between WT and CTNS−/− Day 4 myotubes. Each dot represents an individual image field, data are show the median with 95% CI (n = 5). **p < 0.01; ns, nonsignificant, p > 0.05. Statistical testing was performed with an unpaired t test. (D) Binary mask images and branching points of representative images of WT and CTNS−/− Day 4 myotubes. Figure S3: Analysis substrates of the mTOR pathway in WT and CTNS −/− myoblasts. (A) Representative western blot analysis of (p)S6 and (p)70S6K1 protein expression in WT and CTNS−/− myoblasts under different feeding conditions. 4‐h incubation with EBSS was used as the starvation condition. Samples normalized for total proteins of vinculin. (B) Quantification of (p)S6 and (p)70S6K1 protein expression in WT and CTNS−/− myoblasts (n = 3 independent experiments). Samples normalized for total proteins of vinculin. Statistical testing was performed with a one‐way ANOVA, Sidak's multiple comparison test. ***p < 0.001; **p < 0.01; ns, nonsignificant, p > 0.05. Figure S4: The RyR‐mediated Ca 2+ release remains unaltered in CTNS − [file JCSM-16-e70116-s002.zip › jcsm70116-sup-0002_Supplementary_Figures/SupFig8.PNG]

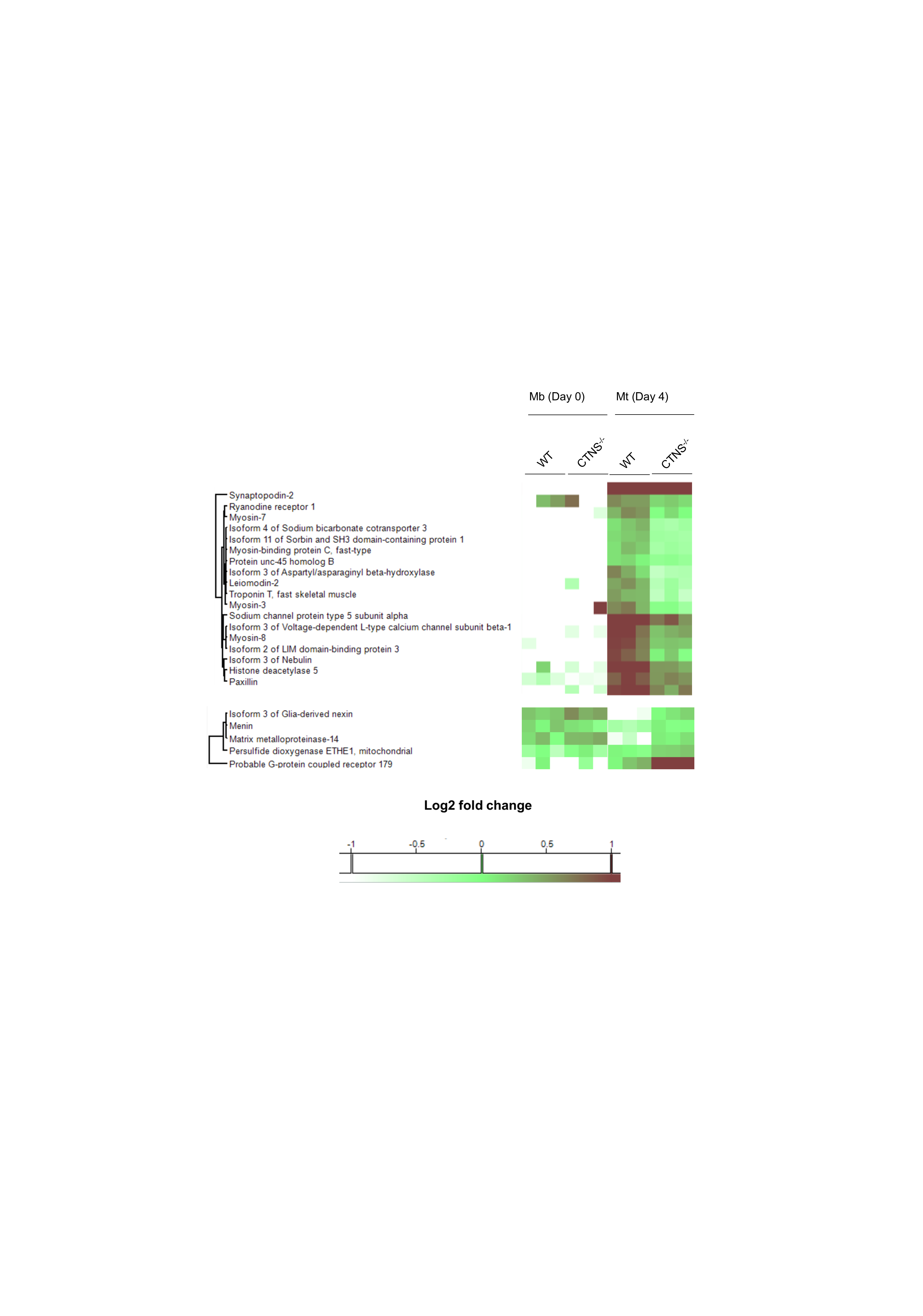

Supplement: Supplementary file 2 — Figure S1: Validation of CTNS knock‐out in immortalized human myoblasts at cDNA level. (A) Quantification of indel events in CRISPR‐edited CTNS−/− myoblasts at cDNA level using DECODR analysis. (B) Modification and predicted stop codon (*) in exon 5 resulting in a 58 AA truncated protein (SnapGene). Figure S2: Analysis of WT and CTNS −/− myotube differentiation. (A) Individual plots of each myogenic differentiation experiment (each n) corresponding to the plot of Figure 2D. Each plot represents 10 ROIs, error bars represent median with 95% CI, numeric values correspond to median. Statistical testing was performed with an unpaired t test. (B)Representation of medians of the five replicates of fusion index, normalized to WT. Statistical testing was performed with one sample t and Wilcoxon signed rank test. (C) Covered area by myotube per region of interest (ROI), number of myotubes per ROI and branching points per myotube between WT and CTNS−/− Day 4 myotubes. Each dot represents an individual image field, data are show the median with 95% CI (n = 5). **p < 0.01; ns, nonsignificant, p > 0.05. Statistical testing was performed with an unpaired t test. (D) Binary mask images and branching points of representative images of WT and CTNS−/− Day 4 myotubes. Figure S3: Analysis substrates of the mTOR pathway in WT and CTNS −/− myoblasts. (A) Representative western blot analysis of (p)S6 and (p)70S6K1 protein expression in WT and CTNS−/− myoblasts under different feeding conditions. 4‐h incubation with EBSS was used as the starvation condition. Samples normalized for total proteins of vinculin. (B) Quantification of (p)S6 and (p)70S6K1 protein expression in WT and CTNS−/− myoblasts (n = 3 independent experiments). Samples normalized for total proteins of vinculin. Statistical testing was performed with a one‐way ANOVA, Sidak's multiple comparison test. ***p < 0.001; **p < 0.01; ns, nonsignificant, p > 0.05. Figure S4: The RyR‐mediated Ca 2+ release remains unaltered in CTNS − [file JCSM-16-e70116-s002.zip › jcsm70116-sup-0002_Supplementary_Figures/SupFig9.PNG]
